# Comparative effectiveness of traditional Chinese medicine injections combined with ACEI/ARB for diabetic nephropathy: A systematic review and network meta-analysis

**DOI:** 10.3389/fphar.2025.1543275

**Published:** 2025-07-18

**Authors:** Aijing Li, Maoying Wei, Chan Wu, Dan Yin, Yiting Tang, Yijia Jiang, Churan Wang, Jingyi Guo, Anning Sun, Xin Gu, Yanbing Gong

**Affiliations:** ^1^ Dongzhimen Hospital, Beijing University of Chinese Medicine, Beijing, China; ^2^ Beijing University of Chinese Medicine, Beijing, China

**Keywords:** traditional Chinese medicine injections, diabetic nephropathy, angiotensin-converting enzyme inhibitors, network meta-analysis, systematic review, angiotensin II receptor blockers

## Abstract

**Aim of the study:**

This systematic review and network meta-analysis aimed to evaluate the comparative effectiveness of traditional Chinese medicine injections (TCMIs) combined with angiotensin-converting enzyme inhibitors or angiotensin Ⅱ receptor blockers for diabetic nephropathy (DN).

**Methods:**

Ten databases were searched. Primary endpoint indicators were urinary albumin excretion rate (UAER) and serum creatinine (Scr). Secondary endpoint indicators were blood urea nitrogen (BUN), urinary β_2_-microglobulin, total cholesterol, triglyceride, systolic blood pressure, and total effective rate. Cochrane risk of bias tool (version 2.0) was used to evaluate the quality of the studies. The GRADE method was used to assess the whole network. Finally, Stata 16.0 software was used to perform network meta-analysis.

**Results:**

A total of 99 randomised controlled trials and ten TCMIs were included for analysis. Based on the surface under the cumulative ranking curve values, it was observed that the efficacy of the combination group was better than that of the control group. For the primary endpoints, the Shuxuetong and Shenkang injections were excellent in reducing UAER and Scr, respectively. The Danshen injection was the most effective for the total effective rate and BUN; the Shuxuetong, Yinxingdamo, Danshen-Chuanxiongqin, and Shuxuening injections were the most effective for total cholesterol, β_2_-microglobulin, triglyceride, and systolic blood pressure, respectively. In terms of dual indicators, for UAER and Scr, the Danshen injection may be the most effective treatment. In addition, no significant adverse reactions were reported in the relevant studies on the Huangqi and Gegensu injections, whereas the Yinxingdamo, Danshen-Chuanxiongqin, Shenkang, Shuxuetong, and Kudiezi injections demonstrated varying degrees of adverse reactions.

**Conclusion:**

In this study, it is indicated that when combined with ACEI/ARB, the Shuxuetong, Shenkang, Danshen, Danshen-Chuanxiongqin, Yinxingdamo, and Shuxuening injections may confer advantages in improving DN indicators. However, due to limitations in the methodological quality of the included studies (especially deficiencies in randomisation and blinding) and the critical lack of reporting on key information regarding TCMI components, the reliability of these findings is compromised.

## 1 Introduction

Diabetic nephropathy (DN) is a disease in which persistent hyperglycaemia induces haemodynamic abnormalities, metabolic disturbances, and inflammatory responses, ultimately leading to renal dysfunction. Clinically, the disease manifests as persistent proteinuria with a progressive decrease in the glomerular filtration rate. DN is one of the most serious complications of diabetes and is the major cause of end-stage renal disease (Tuttle et al., 2014; Qi et al., 2020; Jung and Yoo, 2022). It is predicted that by the middle of the century, diabetes will affect approximately 700 million people worldwide and that approximately 40% of these will develop DN (Saeedi et al., 2019). DN affects more than 50% of patients entering the dialysis or kidney transplant programmes (DeFronzo et al., 2021). The incidence of the disease continues to increase, which not only exerts significant pressure on the global economy but also has a profound impact on the quality of life of patients. Therefore, early detection of the condition and optimisation of existing treatment regimens may delay the onset and progression of DN and reduce the number of deaths from the development of end-stage renal disease. Currently, treatment options for DN primarily focus on lifestyle modifications, glycaemic control, blood pressure management, and blockade of the renin–angiotensin system (Thomas et al., 2016).

Research has demonstrated that angiotensin-converting enzyme inhibitor (ACEI) or angiotensin Ⅱ receptor blocker (ARB) (collectively ACEI/ARB) possesses renoprotective properties and improves glomerular hyperfiltration to some extent (Wada and Makino, 2013; Deng et al., 2022). However, the use of renin–angiotensin system blockers may cause patients to experience adverse effects such as cough, laryngeal oedema, hyperkalaemia, and hypovolaemia (Bilen et al., 2023). The combination of ACEI and ARB may even worsen the development of DN (Fried et al., 2013). Therefore, although current therapies show some efficacy, their limitations have encouraged research workers to explore complementary treatment strategies. Traditional Chinese medicine (TCM) has provided a novel direction for research that may offer some degree of synergistic treatment. TCM injections (TCMIs) are sterile preparations extracted and purified from herbal medicines, enhancing the bioavailability and efficacy of herbal therapies (Xie et al., 2021; Long et al., 2023). Several meta‐analyses have been conducted to show that a variety of injections are effective in lowering the urinary albumin excretion rate (UAER), blood urea nitrogen (BUN), or serum creatinine (Scr) in patients with DN, such as the Huangqi (HQ), Shenkang (SK), and Shuxuetong (ST) injections, among others (Wang R. H. et al., 2019; Wang and Xu, 2020; Zong et al., 2022). However, the comparison of the efficacy of different herbal injections for the treatment of DN is unclear. In this study, we used a network meta-analysis to synthesise evidence from relevant randomised controlled trials (RCTs) to investigate the optimal regimen of TCMIs by comparing the efficacy of different TCMIs in combination with ACEI/ARB in the treatment of DN.

## 2 Materials and methods

The network meta-analysis was registered in the International Platform of Registered Systematic Review and Meta-analysis Protocols (INPLASY) in September 2024 under the registration number INPLASY202490069 (refer to Supplementary Appendix Figure A1). In addition, in this study, we followed the Preferred Reporting Items for Systematic Reviews and Meta-Analyses (PRISMA) and the extension statement for network meta-analyses (PRISMA-NMA) (Page et al., 2021). Details are provided in Supplementary Appendix Table A.1.

### 2.1 Standard evaluation of TCM

To enhance the accuracy, the TCMIs in this study were reported in accordance with the Consensus statement on the Phytochemical Characterisation of Medicinal Plant extracts (ConPhyMP). Concurrently, on 18 October 2024, we standardised the scientific names of the botanical drug components with reference to “A working list of all plants list” (http://www.theplantlist.org). In addition, plants name have been checked with “The World Flora Online” (WFO, http://www.worldfloraonline.org/). Summary tables describing the composition of the ingredients and how they were reported in the original study were prepared based on principles outlined in the four pillars of ethnopharmacology. The composition and standardised name for each injection are presented in Table 1. In addition, the TCMIs have been approved by the China Food and Drug Administration (CFDA) and have been widely used in clinical practice. Further information on the injections included in this study was obtained from the China Medical Information Platform (CMIP), details of which are given in Supplementary Appendix Tables A.2, A.3.

**TABLE 1:** Composition of TCMIs.

Injection names (Chinese)	Composition	Source species (family)	Level of reporting in the original study
Huangqi (黄芪注射液)	*Astragalus mongholicus* Bunge	Fabaceae	Inadequate
Yinxingdamo (银杏达莫注射液)	*Ginkgo biloba* total flavonoids Dipyridamole	Ginkgoaceae	Inadequate
Shuxuening (舒血宁注射液)	*Ginkgo biloba* L. Vitamin C Propylene Glycol Sodium Metabisulphite Disodium Edetate Sodium Citrate	Ginkgoaceae	Inadequate
Danshen-Chuanxiongqin (丹参川芎嗪注射液)	*Salvia miltiorrhiza* Bunge Tetramethylpyrazine Hydrochloride	Lamiaceae	Inadequate
		Apiaceae	
Danshen (丹参注射液)	*Salvia miltiorrhiza* Bunge	Lamiaceae	Inadequate
Danhong (丹红注射液)	*Salvia miltiorrhiza* Bunge *Carthamus tinctorius* L. Sodium Hydroxide	Lamiaceae	Inadequate
		Asteraceae	
Shenkang (肾康注射液)	*Rheum palmatum L.* *Astragalus mongholicus* Bunge *Salvia miltiorrhiza* Bunge *Carthamus tinctorius* L.	Polygonaceae	Inadequate
		Fabaceae	
		Lamiaceae	
		Asteraceae	
Kudiezi (苦碟子注射液)	*Ixeris sonchifolia* (Maxim.) Hance	Asteraceae	Inadequate
Shuxuetong (疏血通注射液)	*Leech (Hirudo)* *Pheretima*	Haemopidae	Inadequate
		Megascolecidae	
Gegensu (葛根素注射液)	*Puerarin*	Fabaceae	Inadequate

### 2.2 Search strategy

We conducted a comprehensive search across ten databases, including China National Knowledge Infrastructure (CNKI), the Chinese Scientific Journal database (VIP), Wanfang database, SinoMed, PubMed, Web of Science, Embase, Cochrane Library, Scopus, and Chinese Clinical Trial Registry (ChiCTR), from their inception to September 2024. Search terms included but were not limited to “diabetic nephropathy,” “diabetic kidney disease,” “glomerulosclerosis diabetic,” “injection,” “angiotensin-converting enzyme inhibitors,” “angiotensin receptor antagonists,” “ACEI,” “ARB,” “randomised controlled trial,” and “RCT”; further details are given in Supplementary Appendix Tables A.4–A.13.

### 2.3 Inclusion and exclusion criteria

#### 2.3.1 Inclusion criteria

(i) Population: the study population met the diagnostic criteria of the Mogensen classification of DN (Mogensen, 1999), with no gender or age restrictions. (ii) Intervention and control: the control group underwent routine treatment plus ACEI/ARB, and the combination group was the control group plus TCMI. (iii) Outcomes: primary endpoints were UAER and Scr. Secondary endpoints were BUN, β_2_-microglobulin (β_2_-MG), total cholesterol (TC), triglyceride (TG), systolic blood pressure (SBP), and total effective rate (TER) (TER in this study was defined as an improvement in the patient’s symptoms and a decrease in UAER, BUN, or Scr). (iv) Study design: RCTs with or without blinding.

#### 2.3.2 Exclusion criteria

The exclusion criteria included the following: non-randomised controlled trials; interventions involving other Chinese medical treatments or injections of other non-Chinese medicines; inability to extract valid primary data from the study; combination of other diseases affecting renal function in the included patients; duplication and inaccessibility of full text; and meta-analyses, reviews, case reports, animal experiments, conference papers, *etc*., and the number of included studies was less than two.

### 2.4 Study selection process and data extraction

All of the retrieved studies were imported into EndNote 20 (Clarivate Analytics, Philadelphia, PA, United States), followed by the removal of duplicate articles. The screening process comprised four steps: first, two reviewers independently screened titles and abstracts using inclusion and exclusion criteria. Second, the full text of the literature screened in the first step was downloaded and thoroughly reviewed to determine whether it met the inclusion criteria. Disagreements were resolved by discussion between the reviewers, and a third reviewer was consulted if necessary. Third, two reviewers performed data extraction for the included studies. The extracted data included the following: (i) basic information: first author and year of publication; (ii) patient aspects: sample size of each group, gender, mean age, duration of treatment, interventions (ACEI/ARB), and duration of disease; and (iii) outcome aspects: available outcome indicators included in each study. Fourth, in cases of missing data or incomplete reporting, we prioritized contacting the corresponding authors via email with standardized requests, allowing a 14-day response window and a maximum of two contact attempts. If no response was received, we used data imputation strategies according to the Cochrane Handbook for Systematic Reviews of Interventions (Higgins et al., 2024).

### 2.5 Risk of bias assessment

The quality of the included studies was individually assessed by two reviewers using the Cochrane risk of bias tool (version 2.0) (Sterne et al., 2019), including the randomisation process, deviations from intended interventions, missing outcome data, measurement of the outcome, and selection of reported result. Risk of bias was categorised as “low risk,” “high risk,” and “some concerns.” In addition, the entire network was individually assessed by two reviewers using the GRADE approach (Puhan et al., 2014), which provided a framework for rating the certainty of each pairwise comparison evidence as “high,” “moderate,” “low,” or “very low.” Disagreements arising from the above assessments were resolved through discussion between the reviewers.

### 2.6 Statistical analysis

A random-effects model was used in this study, and we used Stata 16.0 software (Stata Corp, College Station, Texas, United States) to perform network meta-analyses. For dichotomous variables, based on the overall efficacy in patients with DN, the odds ratio (OR) with 95% confidence interval (95% CI) was used as the effect indicator. For continuous variables, such as TC, TG and SBP, the mean difference (MD) and 95% CI were calculated, but when data units were inconsistent, such as UAER, BUN, Scr, and β_2_-MG, the standardised mean difference (SMD) and 95% CI were calculated. We calculated the surface under the cumulative ranking curve (SUCRA) values to rank multiple interventions. SUCRA values of 100% and 0% were the outcomes of treatments with the best efficacy and the worst safety, respectively. We conducted a two-dimensional cluster analysis to further explore the rankings. We performed Egger’s regression test and created a funnel plot to evaluate potential publication bias in the intervention network. Finally, we tested the heterogeneity of effects across populations through subgroup analyses and used sensitivity analyses to verify the robustness of the results.

## 3 Results

### 3.1 Literature selection and study characteristics

**FIGURE 1:**
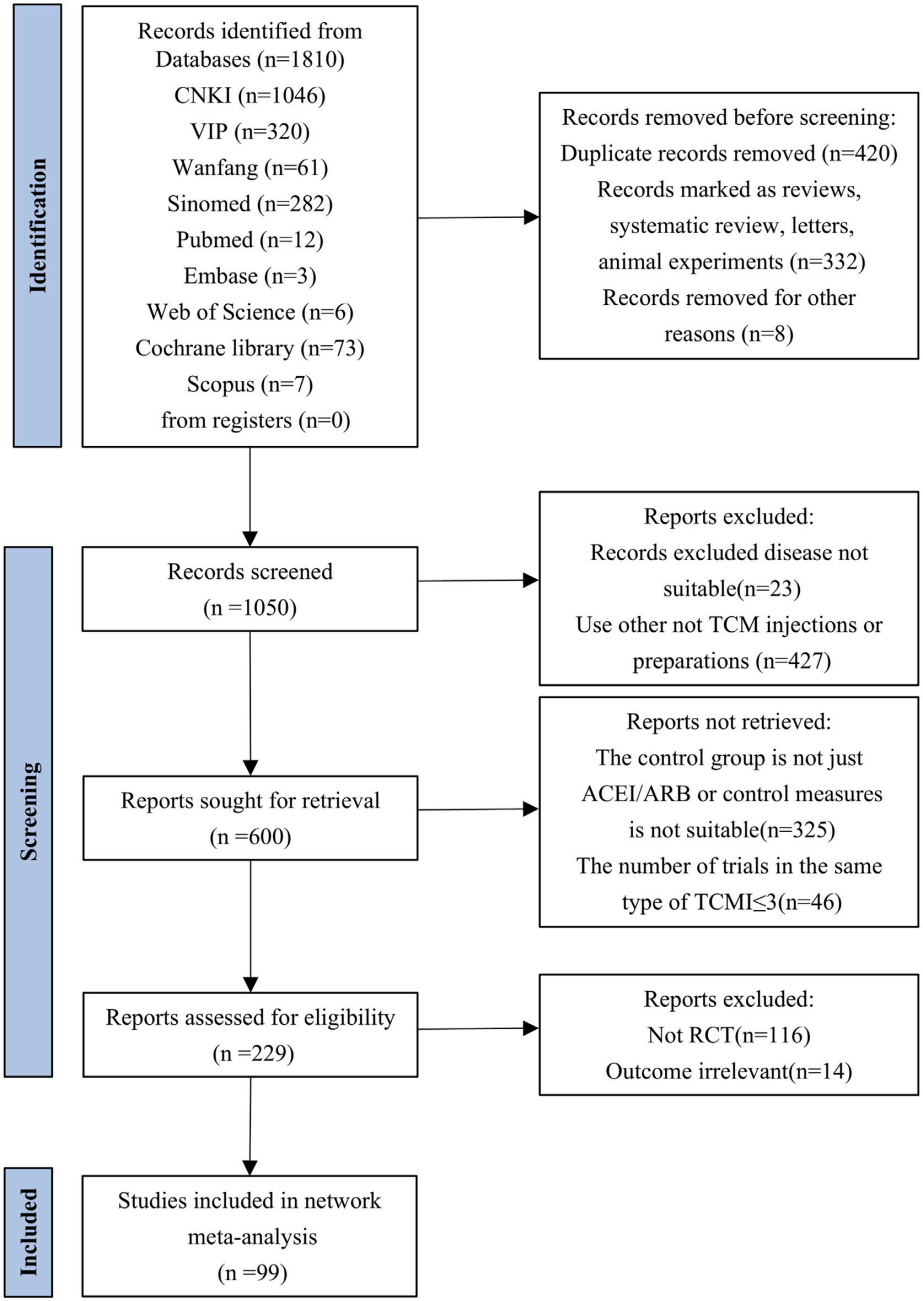
Flowchart of searching and screening steps for the studies.

We retrieved 1,810 citations from the database. After excluding duplicates or ineligible records, 99 studies with 9,888 participants in 10 TCMIs met the eligibility criteria for inclusion in the network meta-analysis. The specific screening process is illustrated in Figure 1. Two updates were made during the screening process. All the included studies compared the effect of a single injection combined with ACEI/ARB versus ACEI/ARB alone. The specific interventions of the original study are given in Supplementary Appendix Table A.14. All the trials included in the analysis were RCTs, each with between 40 and 358 participants. In addition, all the trials were published between 2003 and 2024. The duration of treatment varied from 2 to 36 weeks. Baseline characteristics are given in Supplementary Appendix Table A.15. Among all the TCMIs, HQ was the treatment with the highest number of studies (33 RCTs), followed by the Yinxingdamo injection (YX) (17 RCTs), the SK injection (12 RCTs), the Danshen-Chuanxiongqin injection (DC) (nine RCTs), the Danhong injection (DH) (seven RCTs), the Danshen injection (DS) (five RCTs), the Shuxuening injection (SX) (four RCTs), the Kudiezi injection (KD) (four RCTs), the ST injection (four RCTs), and the Gegensu injection (GG) (four RCTs).

### 3.2 Quality assessment of evidence

**FIGURE 2:**
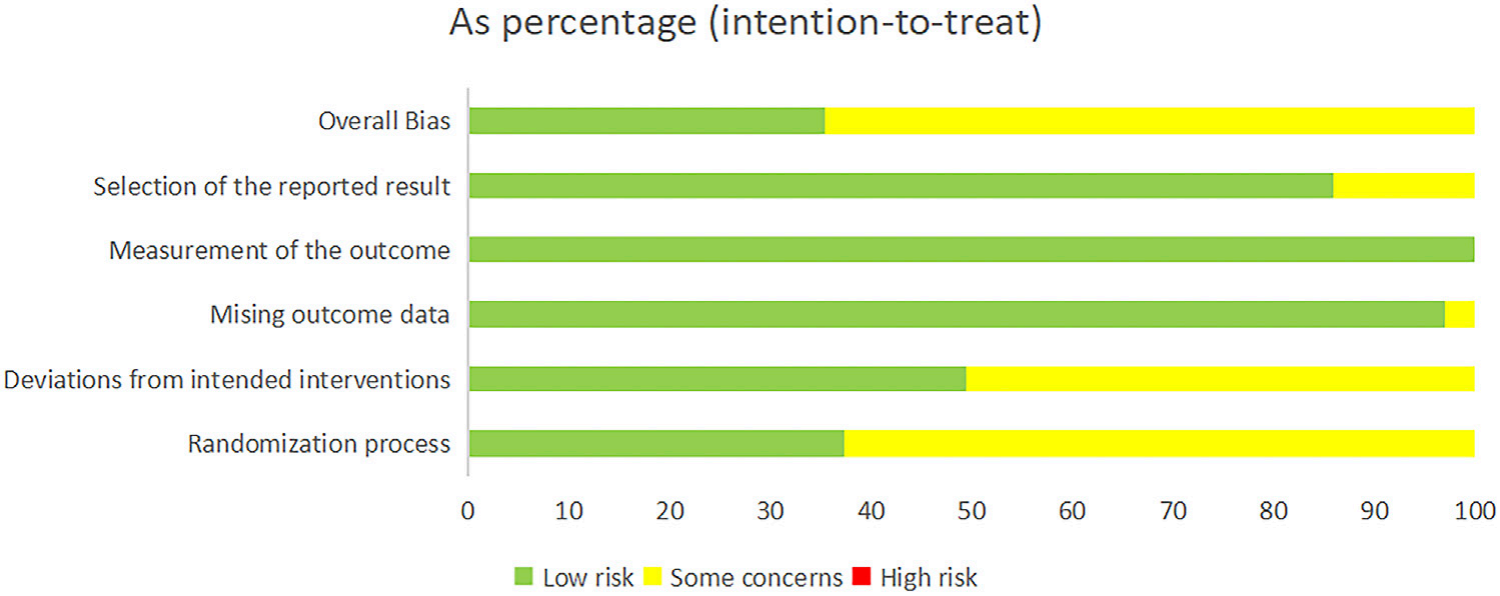
Risk of bias graph.

Among the 99 studies included, 37 studies provided detailed information on the method of randomisation. In contrast, the remaining studies only mentioned randomisation and did not provide specific information on the details of randomisation. Five studies (Xu, 2008; Rao et al., 2013; Zheng, 2013; Fan, 2017; Hao et al., 2022) reported programme concealment, allocation concealment, and measurement blinding. Three studies had incomplete data results, but this did not affect the completeness of the main outcome indicators (Wang, 2012; Zhao, 2014; Li and Zhang, 2015). In all the studies, the data were analysed using sound statistical methods. In addition, it was noted that the literature included was all from China, but none of the studies described a pre-established research plan or analysis protocol. As we all know, ChiCTR was established in 2005, and the requirements for selective reporting have become more standardised in the recent years. Consequently, the 14 older studies (Zhu et al., 2003; Huang et al., 2004; Jiang et al., 2005; Ma, 2006; Li, 2007; Yang et al., 2007; Cui, 2009; Li et al., 2009; Li, 2009; Liu et al., 2009; Tian et al., 2009; Xu, 2009; Yuan, 2009; Zhou et al., 2009) were identified as being at risk of selective reporting, whereas other trials exhibited a lower risk of such bias, as illustrated in Figure 2. The results of the risk of bias assessment for the included studies are given in Supplementary Appendix Table A.16. The presence of only indirect comparisons resulted in lower quality ratings for the vast majority of two-by-two comparisons. Details of the GRADE method of evidence assessment are given in Supplementary Appendix Tables A.17–A.24.

### 3.3 Network meta-analysis

#### 3.3.1 UAER

**FIGURE 3:**
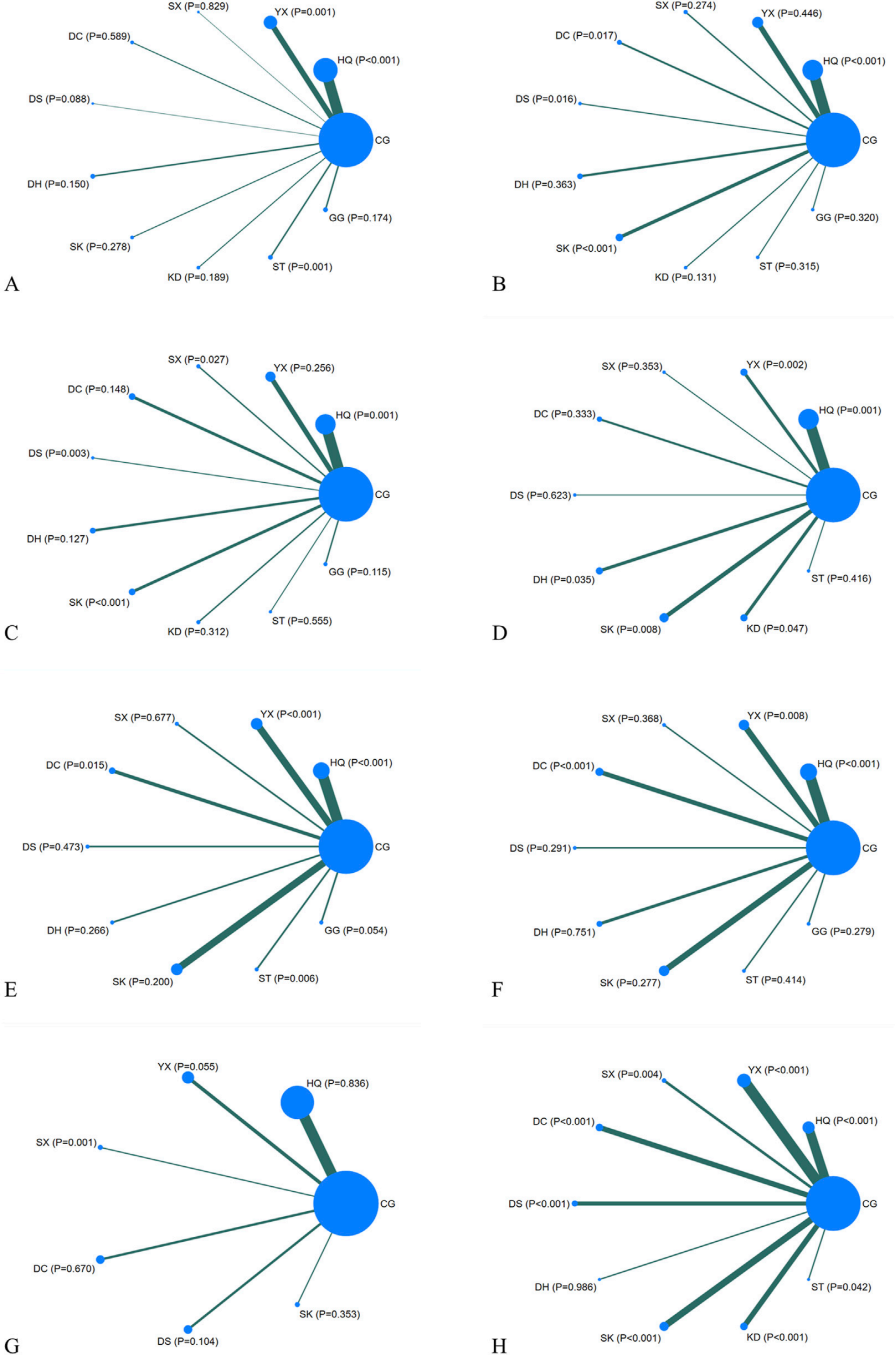
Network diagrams. CG, control group; ST, Shuxuetong injection; SK, Shenkang injection; YX, Yinxingdamo injection; DC, Danshen-Chuanxiongqin injection; DS, Danshen injection; HQ, Huangqi injection; GG, Gegensu injection; DH, Danhong injection; SX, Shuxuening injection; KD, Kudiezi injection. **(A)** Urinary albumin excretion rate; **(B)** serum creatinine; **(C)** blood urea nitrogen; **(D)** β2‐ microglobulin; **(E)** total cholesterol; **(F)** triglyceride; **(G)** systolic blood pressure; **(H)** total effective rate.

**FIGURE 4:**
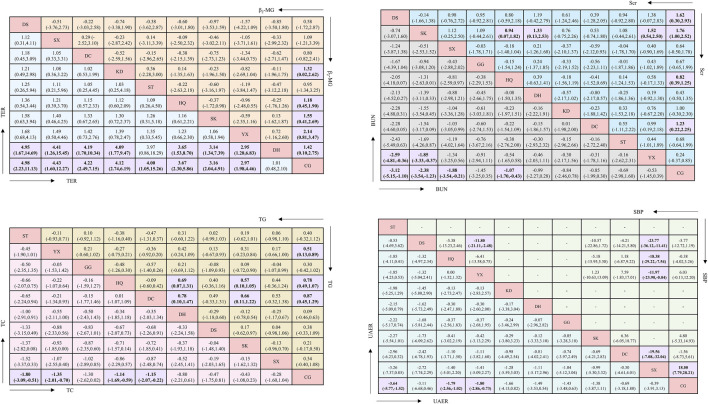
League table. Significant effects are printed in bold and highlighted in purple.

**FIGURE 5:**
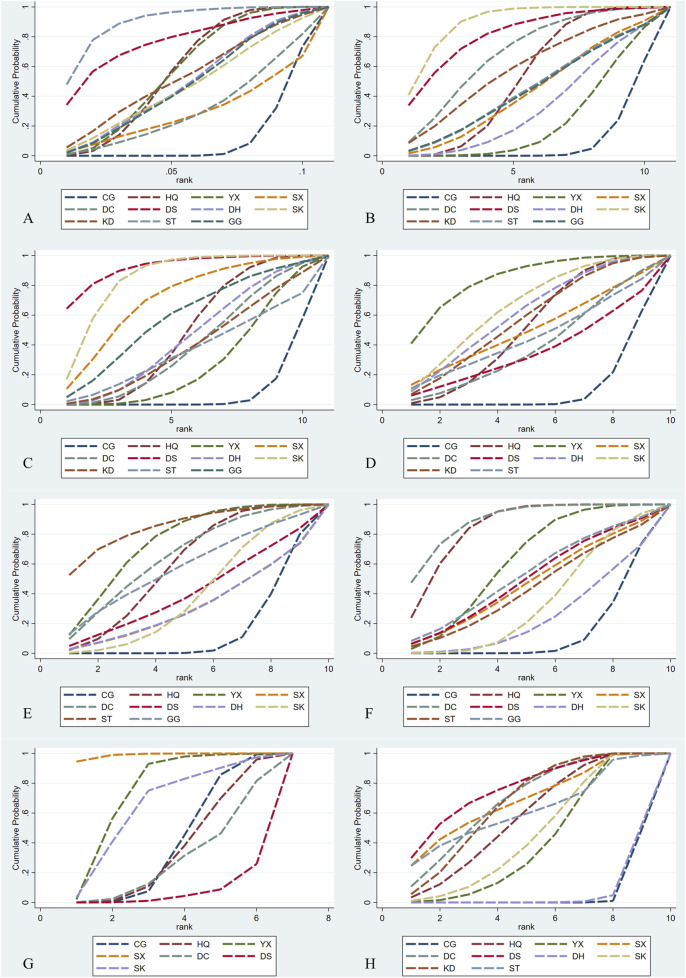
SCURA diagrams. CG, control group; ST, Shuxuetong injection; SK, Shenkang injection; YX, Yinxingdamo injection; DC, Danshen-Chuanxiongqin injection; DS, Danshen injection; HQ, Huangqi injection; GG, Gegensu injection; DH, Danhong injection; SX, Shuxuening injection; KD, Kudiezi injection. **(A)** Urinary albumin excretionrate; **(B)**serum creatinine; **(C)** blood urea nitrogen; **(D)**β2‐ microglobulin; **(E)** total cholesterol; **(F)** triglyceride; **(G)** systolic blood pressure; **(H)** total effective rate.

Regarding UAER, 49 studies with a total of 4,121 patients were included, evaluating eleven interventions with ten TCMIs, as illustrated in Figure 3A. The following combination groups did not demonstrate statistically significant difference in efficacy compared with the control group, including DS, KD, DH, GG, SK, DC, and SX. Three TCMIs were superior to ACEI/ARB alone in combination with ACEI/ARB: ST (SMD: −3.64, 95% CI: −5.77, −1.52) (p = 0.001), HQ (SMD: −1.79, 95% CI: −2.56, −1.02) (p < 0.001), and YX (SMD: −1.80, 95% CI: −2.86, −0.73) (p = 0.001), as illustrated in Figure 4. According to the SUCRA area, in combination with ACEI/ARB, ST (SUCRA = 90.2%) demonstrated the best therapeutic effect, followed by DS (SUCRA = 77.1%), HQ (SUCRA = 57.3%), YX (SUCRA = 57.2%), KD (SUCRA = 52.8%), DH (SUCRA = 49.1%), GG (SUCRA = 47.8%), SK (SUCRA = 46.8%), DC (SUCRA = 31.2%), SX (SUCRA = 29.0%), and ACEI/ARB (SUCRA = 11.5%), as illustrated in Figure 5A. Details are given in Supplementary Appendix Table A.25.

#### 3.3.2 Scr

Regarding Scr, 78 studies with a total of 7,872 patients were included, evaluating eleven interventions in ten TCMIs, as illustrated in Figure 3B. The following combination groups demonstrated no statistically significant difference in efficacy compared to the control group: KD, ST, GG, SX, DH, and YX. Four TCMIs were superior to ACEI/ARB alone in combination with ACEI/ARB: SK (SMD: −1.76, 95% CI: −2.52, −1.00) (p < 0.001), DS (SMD: −1.62, 95% CI: −2.93, −0.30) (p = 0.016), DC (SMD: −1.23, 95% CI: −2.25, −0.22) (p = 0.017), and HQ (SMD: −0.82, 95% CI: −1.25, −0.39) (p < 0.001), as illustrated in Figure 4. According to the SUCRA area, in combination with ACEI/ARB, SK (SUCRA = 89.7%) demonstrated the best therapeutic effect, followed by DS (SUCRA = 81.2%), DC (SUCRA = 69.4%), KD (SUCRA = 58.3%), HQ (SUCRA = 52.7%), ST (SUCRA = 45.0%), GG (SUCRA = 44.7%), SX (SUCRA = 43.7%), DH (SUCRA = 32.9%), YX (SUCRA = 23.0%), and ACEI/ARB (SUCRA = 9.4%), as illustrated in Figure 5B. Details are given in Supplementary Appendix Table A.25.

#### 3.3.3 BUN

Regarding BUN, 62 studies with a total of 6,115 patients were included, evaluating eleven interventions in ten TCMIs, as illustrated in Figure 3C. The following combination groups demonstrated no statistically significant difference in efficacy compared to the control group: GG, DH, KD, DC, ST, and YX. Four TCMIs were superior to ACEI/ARB alone in combination with ACEI/ARB: DS (MD: −3.12, 95% CI: −5.51, −1.10) (p = 0.003), SK (MD: −2.38, 95% CI: −3.54, −1.23) (p < 0.001), SX (MD: −1.88, 95% CI: −3.54, −0.21) (p = 0.027), and HQ (MD: −1.07, 95% CI: −1.70, −0.43) (p = 0.001), as illustrated in Figure 4. According to the SUCRA area, in combination with ACEI/ARB, DS (SUCRA = 92.3%) demonstrated the best therapeutic effect, followed by SK (SUCRA = 84.7%), SX (SUCRA = 71.2%), GG (SUCRA = 58.3%), HQ (SUCRA = 48.1%), DH (SUCRA = 45.0%), DC (SUCRA = 39.7%), KD (SUCRA = 39.0%), ST (SUCRA = 36.1%), YX (SUCRA = 27.8%), and ACEI/ARB (SUCRA = 7.8%), as illustrated in Figure 5C. Details are given in Supplementary Appendix Table A.25.

#### 3.3.4 β_2_-MG

Regarding β_2_-MG, 28 studies with a total of 3,067 patients were included, evaluating ten interventions in nine TCMIs, as illustrated in Figure 3D. The following combination groups demonstrated no statistically significant difference in efficacy compared to the control group: SX, ST, DC, and DS. Five TCMIs were superior to ACEI/ARB alone in combination with ACEI/ARB: YX (SMD: −2.14, 95% CI: −3.47,-0.81) (p = 0.002), SK (SMD: −1.55, 95% CI: −2.69, −0.14) (p = 0.008), DH (SMD: −1.42, 95% CI: −2.75, −0.10) (p = 0.035), KD (SMD: −1.32, 95% CI: −2.62, −0.02) (p = 0.047), and HQ (SMD: −1.18, 95% CI: −1.90, −0.45) (p = 0.001), as illustrated in Figure 4. According to the SUCRA area, in combination with ACEI/ARB, YX (SUCRA = 84.4%) demonstrated the best therapeutic effect, followed by SK (SUCRA = 66.1%), DH (SUCRA = 61.1%), KD (SUCRA = 57.3%), HQ (SUCRA = 51.7%), SX (SUCRA = 49.9%), ST (SUCRA = 44.9%), DC (SUCRA = 39.2%), DS (SUCRA = 35.5%), and ACEI/ARB (SUCRA = 9.8%), as illustrated in Figure 5D. Details are given in Supplementary Appendix Table A.25.

#### 3.3.5 TC

Regarding TC, 21 studies with a total of 2,270 patients were included, evaluating ten interventions in nine TCMIs, as illustrated in Figure 3E. The following combination groups demonstrated no statistically significant difference in efficacy compared to the control group: GG, DH, DS, SK, and SX. Four TCMIs were superior to ACEI/ARB alone in combination with ACEI/ARB: ST (MD: −1.80, 95% CI: −3.09, −0.51) (p = 0.006), YX (MD: −1.35, 95% CI: −2.01, −0.70) (p < 0.001), HQ (MD: −1.14, 95% CI: −1.69, −0.59) (p < 0.001), and DC (MD: −1.15, 95% CI: −2.07, −0.22) (p = 0.015), as illustrated in Figure 4. According to the SUCRA area, in combination with ACEI/ARB, ST (SUCRA = 85.3%) demonstrated the best therapeutic effect, followed by YX (SUCRA = 74.5%), DC (SUCRA = 65.2%), HQ (SUCRA = 59.4%), GG (SUCRA = 57.6%), DS (SUCRA = 40.7%), SK (SUCRA = 39.4%), DH (SUCRA = 31.6%), SX (SUCRA = 31.5%), and ACEI/ARB (SUCRA = 14.8%), as illustrated in Figure 5E. Details are given in Supplementary Appendix Table A.25.

#### 3.3.6 TG

Regarding TG, 24 studies with a total of 2,388 patients were included, evaluating ten interventions in nine TCMIs, as illustrated in Figure 3F. The following combination groups demonstrated no statistically significant difference in efficacy compared to the control group: ST, DS, SX, GG, SK, and DH. Three TCMIs were superior to ACEI/ARB alone in combination with ACEI/ARB: DC (MD: −0.87, 95% CI: −1.29, −0.45) (p < 0.001), HQ (MD: −0.78, 95% CI: −1.07, −0.49) (p < 0.001), and YX (MD: −0.51, 95% CI: −0.89, −0.13) (p = 0.008), as illustrated in Figure 4. According to the SUCRA area, in combination with ACEI/ARB, DC (SUCRA = 89.1%) demonstrated the best therapeutic effect, followed by HQ (SUCRA = 84.7%), YX (SUCRA = 62.1%), GG (SUCRA = 52.3%), DS (SUCRA = 49.5%), SX (SUCRA = 47.1%), ST (SUCRA = 43.3%), SK (SUCRA = 34.3%), DH (SUCRA = 24.4%), and ACEI/ARB (SUCRA = 13.2%), as illustrated in Figure 5F. Details are given in Supplementary Appendix Table A.25.

#### 3.3.7 SBP

Regarding SBP, 18 studies with a total of 1,746 patients were included, evaluating seven interventions in six TCMIs, as illustrated in Figure 3G. Three TCMIs in combination with ACEI/ARB were superior to ACEI/ARB alone: SX, YX, and SK, as illustrated in Figure 4. Unfortunately, only SX (MD: −18.00, 95% CI: −28.21, −7.79) (p = 0.001) demonstrated statistically significant difference. DS, DC, and HQ did not show a statistically significant therapeutic effect on lowering SBP compared with the control group. According to the SUCRA area, in combination with ACEI/ARB, SX (SUCRA = 98.9%) demonstrated the best therapeutic effect, followed by YX (SUCRA = 74.5%), SK (SUCRA = 65.4%), ACEI/ARB (SUCRA = 40.1%), HQ (SUCRA = 35.9%), DC (SUCRA = 28.6%), and DS (SUCRA = 6.5%), as illustrated in Figure 5G. Details are given in Supplementary Appendix Table A.25.

#### 3.3.8 TER

Regarding TER, 35 studies with a total of 4,382 patients were included, evaluating ten interventions in nine TCMIs, as illustrated in Figure 3H. The comparison of the difference in TER between DH and ACEI/ARB alone was not statistically significant. The following combination groups demonstrated superior TER than the control group: DS (OR: 4.98, 95% CI: 2.23, 11.13) (p < 0.001), SX (OR: 4.43, 95% CI: 1.60, 12.27) (p = 0.004), DC (OR: 4.22, 95% CI: 2.49, 7.15) (p < 0.001), KD (OR: 4.12, 95% CI: 2.74, 6.19) (p < 0.001), ST (OR: 4.00, 95% CI: 1.05, 15.26) (p = 0.042), HQ (OR: 3.67, 95% CI: 2.30, 5.86) (p < 0.001), SK (OR: 3.16, 95% CI: 2.04, 4.91) (p < 0.001), and YX (OR: 2.97, 95% CI: 1.98, 4.46) (p < 0.001), as illustrated in Figure 4. This suggests a benefit in improving the main indicators and clinical symptoms of DN. According to the SUCRA area, in combination with ACEI/ARB, DS (SUCRA = 77%) had the best treatment outcome, followed by DC (SUCRA = 68.8%), SX (SUCRA = 68.6%), KD (SUCRA = 67.1%), ST (SUCRA = 61.9%), HQ (SUCRA = 57.7%), SK (SUCRA = 46.1%), YX (SUCRA = 40.6%), DH (SUCRA = 6.4%), and ACEI/ARB (SUCRA = 5.6%), as illustrated in Figure 5H. Details are given in Supplementary Appendix Table A.25.

### 3.4 Safety

A total of 30 studies involving adverse reactions (ARs) were reported. ARs included dizziness, cough, headache, rash, nausea, tinnitus, hypotension, tachycardia, itchiness, fatigue, shortness of breath, gastrointestinal discomfort, and elevated potassium in blood. ARs occurred in 59 of 1,803 patients in the combination group and 85 of 1,783 patients in the control group. The incidence of ARs in the combination group was 3.3%, which was lower than that observed in the control group (4.8%) (OR: 0.69, 95% CI: 0.50, 0.95) (p = 0.02). There were seven TCMIs in the study: HQ, YX, DC, DS, SK, KD, and ST. It is worth mentioning that no ARs were reported in the studies of HQ and GG. Fortunately, the symptoms observed were relatively mild and showed improvement with symptomatic treatment. Details of the reported safety issues are shown in Supplementary Appendix Table A.26.

### 3.5 Cluster analysis

**FIGURE 6:**
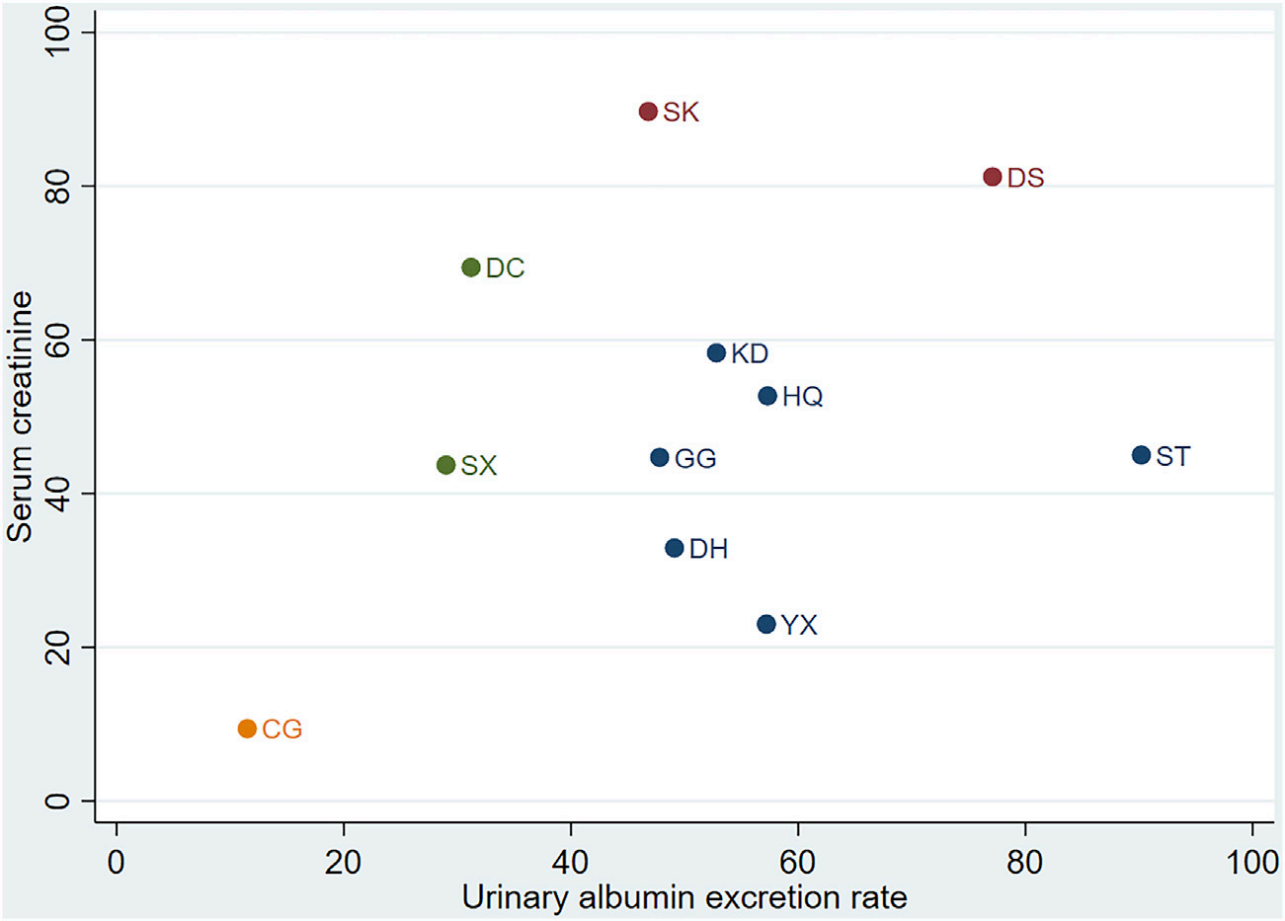
Cluster analysis. CG, control group; ST, Shuxuetong injection; SK, Shenkang injection; YX, Yinxingdamo injection; DC, Danshen-Chuanxiongqin injection; DS, Danshen injection; HQ, Huangqi injection; GG, Gegensu injection; DH, Danhong injection; SX, Shuxuening injection; KD, Kudiezi injection.

According to KDIGO and ADA guidelines (de Boer et al., 2022), the diagnosis and staging of DN relies on the decreased glomerular filtration rate (based on Scr) and UAER. We employed cluster analysis to analyse interventions with multidimensional outcomes. In the combination group, for UAER and Scr, DS may represent the optimal treatment option, as illustrated in Figure 6.

### 3.6 Publication bias

To assess potential publication bias, funnel plots were constructed for all outcome indicators, supplemented by Egger’s regression tests. The results showed that there was some publication bias in the results of UAER, TER, and SBP (p < 0.05) and no significant publication bias in Scr, BUN, β_2_-MG, TC, and TG (p > 0.05), as illustrated in Supplementary Appendix Figure A.2. The detailed results of Egger’s regression tests can be found in Supplementary Appendix Table A.27.

### 3.7 Tests of inconsistency and heterogeneity

This study did not form a closed loop, so inconsistency tests could not be performed. The results of the heterogeneity analysis showed that there was no significant heterogeneity in TER (I^2^ = 10.5%) and that there was significant heterogeneity in the other outcomes (I^2^ > 90%). More detailed information can be found in Supplementary Appendix Tables A.28–A.35. The source of heterogeneity was considered to be related to the stage of DN.

### 3.8 Subgroup analysis

We performed subgroup analyses using the specific stages of DN as an inclusion criterion. There were 48 trials of stage III, 34 trials of unknown stage, four trials of stage IV, and 13 trials of multiple stages. Unfortunately, a network meta-analysis of stage IV or multiple stages could not be performed for the primary endpoints because of the small number of included trials. The main results were as follows: in stage III, ST was the best treatment for UAER, which was consistent with the original network meta-analysis; for Scr, GG was the best treatment. In the unspecified stage, GG and DS were the best treatments for UAER and Scr, respectively. However, they were not fully consistent with the original results. Detailed information can be found in Supplementary Appendix Tables A.36–A.38.

### 3.9 Sensitivity analysis

It was taken into account that the concentration of the drug in the body may affect the results of the comparisons of efficacy. We excluded trials with an intervention period of less than 28 days and then performed a network meta-analysis, and the results of the primary endpoints were consistent with the original results. Specific information is provided in Supplementary Appendix Tables A.39, A.40.

## 4 Discussion

### 4.1 Main findings

TCMIs are sterile preparations derived from Chinese herbal materials through extraction and purification processes. They are formulated as solutions, emulsions for direct administration via an injection, or as powders or concentrated solutions that require reconstitution into solutions immediately before clinical use. As a new dosage form, TCMIs are considered a major innovation in the modernisation of Chinese medicine. It is increasingly being developed for a wide range of diseases (Geng et al., 2015; Liu et al., 2023; Ma et al., 2024). However, due to the complexity of TCMI’s composition, its quality control is still not fully resolved. Fortunately, the identification and exploration of relevant components has been ongoing. The CFDA has enhanced and standardized the protocols for the identification, testing, content determination, and pharmacological evaluation of TCMI components, thereby providing clearer evidence to support their safety profiles. Additional details can be found in Supplementary Appendix Tables A.2–A.4.

This network meta-analysis evaluated and compared the efficacy and safety of ten TCMIs in combination with ACEI/ARB in the treatment of DN. As is known, UAER is an important indicator of disease staging in DN. Scr, BUN, and β_2_-MG can reflect glomerular filtration function to some extent. Their monitoring is effective in predicting the progression of DN to a certain extent. ST and SK were excellent in reducing UAER and Scr, respectively. However, subgroup analysis was performed, and it was found that this result was not stable and could be affected by the stage of DN. The results of subgroup analysis need to be considered with caution. In the subgroup analyses, GG had a greater impact on the primary endpoints, probably because GG included only one trial in the primary endpoints. In addition, many studies did not report the stage of DN, which also affected the results of the subgroup analysis. DN is mostly undetected in stages Ⅰ and Ⅱ without obvious clinical manifestations, so the results of the original network meta-analysis may be more reliable than those of the subgroup analysis. DS demonstrated the highest TER in treating DN. However, this finding was based on only three RCTs of limited quality, so it should be treated with caution. Additionally, it exhibited the most significant effect in reducing BUN. YX was excellent in reducing β_2_-MG. For UAER and Scr, DS represented the most effective treatment option. The heterogeneity of several indicators in this study was high, and this study explored the source of heterogeneity by exploring the stage of DN. In fact, conventional treatments and the dosage of different TCMIs may also affect the results. However, the original study had limited coverage of conventional treatment and could not quantitatively compare its impact on outcomes. In addition, the issue of not being able to standardise the dosage of different TCMIs was not known to have an effect on the results. In addition, all of the original studies included in this meta-analysis were conducted in China, and many did not report the use of double-blind procedures or random allocation methods. Concerns have been raised regarding the reproducibility and quality of the original studies, which will affect the results of the meta-analysis. Therefore, more high-quality studies on TCMIs are needed in order to further validate these results.

Many diabetics with inadequate glycaemic control have abnormal lipid profiles. Studies have demonstrated that hypertriglyceridemia is closely related to atherosclerosis in diabetic patients (La Sala et al., 2019; Zhang et al., 2022). So, TC and TG may also reflect renal function to some extent. This study demonstrated that DC was the most effective in reducing TG, whereas ST exhibited superior efficacy in lowering TC. The evidence indicates that in diabetic and non-diabetic nephropathy, a more intensive blood pressure reduction below 140/90 mmHg may produce a favourable effect on renal function, and survival is multi-fold (Grassi et al., 2016). Therefore, blood pressure management is also important in slowing down the progression of DN. In this study, we demonstrated the significant efficacy of SX in lowering SBP in DN patients. However, this finding was based on only one RCT of limited quality, so it should be treated with caution. Furthermore, no significant ARs were observed in the relevant studies on HQ, whereas varying degrees of ARs were reported in others.

The aetiology of DN is complex, including inflammatory responses, dysregulation of glycolipid metabolism, oxidative stress, autophagy, endoplasmic reticulum stress, and immune responses. ST, SK, YX, and DS can optimally improve different renal function indices. Contemporary pharmacological research suggests that the diverse Chinese medicinal components present in these TCMIs may contribute to delaying the progression of DN through multiple pathways.

DS, a single herbal formulation, has shown superior efficacy in reducing BUN. It may preserve glomerular filtration integrity primarily through its anti-inflammatory targeting of the NF-κB/p38 MAPK pathway (Zhang et al., 2018). In contrast, SK, a multi-herbal compound, shows enhanced Scr-lowering effects through synergistic actions. Astragaloside IV inhibits Nox4 signalling-mediated oxidative stress and slows down podocyte apoptosis, whereas salvia components inhibit TGF-β/Smad3-driven fibrosis, collectively improving tubular secretory capacity and metabolic homoeostasis (Huang et al., 2023; Shen et al., 2023; Huang et al., 2024; Liu et al., 2024). This mechanistic divergence may underscore the glomerular-centric action of DS versus the multi-targeted tubulointerstitial remodelling of SK.

DC contains *Salvia miltiorrhiza* and Chuanxiongzine, which can effectively reduce TG. Danshen polysaccharide is the main active component in the aqueous extract of *Salvia miltiorrhiza* and can attenuate immune liver injury in mice by inhibiting the activation of the TLR4/MyD88 signalling pathway (Wang X. et al., 2019). *Ligusticum chuanxiong* alleviates liver injury by regulating nitric oxide biosynthesis and key pathways such as Toll-like receptor, NOD-like receptor, and TNF signalling pathways (Lu et al., 2023). The liver is the main site of cholesterol synthesis. Thus, both synergistically regulate cholesterol metabolism.

YX is an extract of *Ginkgo biloba*. *Ginkgo biloba* extracts attenuate β-glycerophosphate-induced vascular smooth muscle calcification by inhibiting the Wnt/β-catenin signalling pathway. It also inhibits extracellular matrix accumulation, epithelial–mesenchymal marker expression, and endoplasmic reticulum stress (Wang J. et al., 2019; Han et al., 2021). β_2_-MG passes easily through the glomerular filtration membrane, and almost all filtered β_2_-MG is reabsorbed and degraded by proximal tubular cells. It is therefore not difficult to understand why YX is more effective in reducing β_2_-MG. Moreover, *Ginkgo biloba* extract might exert its vascular protective effects by inducing vascular HO-1 expression (Chen et al., 2011). Therefore, the control of SBP is better in all the injections containing *Ginkgo biloba* extract, such as SX and YX.

ST is an extract of leeches and earthworms. It demonstrates dual efficacy in reducing UAER and TC. Hirudin protects the kidney and prevents proteinuria by inhibiting the p38 MAPK signalling pathway, reducing endoplasmic reticulum stress in podocytes and attenuating PAN damage to podocyte cytoskeletal proteins (Long et al., 2022). Aqueous extract of earthworms attenuates oxidative stress-induced renal injury by activating intrarenal Sirt1/Nrf2 cascade and ameliorating mitochondrial damage (Shu et al., 2024). In addition, the aqueous extract of earthworms reduces liver fibrosis in mice through the activation of the hepatic LKB1/Nrf2 signalling pathway, which suppresses hepatic stellate cell activation, thereby reducing triglyceride synthesis (Zhang T. et al., 2024).

### 4.2 Comparison with other studies

To the best of our knowledge, this study was the most comprehensive and up-to-date systematic review and network meta-analysis evaluating almost all the studies of TCMIs used in combination with ACEI/ARB for the treatment of DN. In the past, most of the studies of herbal injections combined with ACEI/ARB for the treatment of DN involved a single TCMI or several TCMIs (Zhu et al., 2022; Zhang Z. et al., 2024). A previous study conducted a network meta-analysis of TCM decoctions that had a large number of ingredients, and it was not possible to control for variable uniqueness. Therefore, there was some bias. The composition of TCMIs is more stable than that of TCM decoctions, which is of quantitative importance. Therefore, this study was well designed to investigate the optimal herbal injections for the treatment of DN in modern clinical applications.

### 4.3 Limitations of the study

First, “holistic diagnosis and treatment” was the essence of TCM. Unfortunately, this study included patients with DN who were given a single injection for treatment, which, to a certain extent, violated the essence in treating diseases in TCM and may have an impact on the results. Second, most of the included studies were of low quality and did not mention the specific method of randomisation allocation and blinding. Thus, not enough information was provided to accurately evaluate randomisation and allocation concealment by the research workers. In addition, some of the included trials did not mention the specific manufacturer of the drug. Third, DH, SX, KD, and GG were included in only four studies, and ST was included in only five studies. In addition, most of the studies did not have a clear safety evaluation. Fourth, previous RCTs have lacked head-to-head studies of herbal injections, adding to the uncertainty of the results. As no closed loops were formed in the analysis, inconsistency assessment was not feasible. Finally, all the literature included was from China.

## 5 Reflection on study design

During the design of the systematic review protocol, detailed definitions of inclusion and exclusion criteria (population, intervention, control, and outcome) were provided in this study, but a standardised PICOTS framework was not used to structure the research question. In future comparative effectiveness studies, clearly defining all elements of PICOTS in advance (particularly the environmental factors within the setting) will facilitate a more comprehensive understanding of the research context and potential sources of heterogeneity, and thereby enhance the rigour and transparency of the protocol design.

The reliability of our findings is severely limited by widespread methodological flaws in the included studies, particularly inadequate randomization, blinding, and critically insufficient reporting of TCMIs. This resulted in low or very low certainty of evidence (GRADE), rendering observed efficacy differences and treatment rankings highly uncertain and likely to change with future high-quality research. The fundamental limitation identified is inadequate TCMI reporting in original RCTs. Lack of chemical composition, source materials, manufacturing processes, and quality control details constitutes a critical weakness in the evidence base, which substantially compromises the reliability and translational potential. Therefore, a paramount priority for future high-quality RCTs must be the comprehensive and transparent reporting of all critical details pertaining to TCMIs used in research.

## 6 Conclusion

In this study, we indicate that when combined with ACEI/ARB, the Shuxuetong, Shenkang, Danshen, Danshen-Chuanxiongqin, Yinxingdamo, and Shuxuening injections may confer advantages in improving DN indicators. However, due to limitations in the methodological quality of the included studies (especially deficiencies in randomisation and blinding) and the critical lack of reporting on key information regarding TCMI components, the reliability of these findings is compromised.

## Data Availability

The original contributions presented in the study are included in the article/Supplementary Material; further enquiries can be directed to the corresponding author.

## Abbreviations

ACEI, angiotensin-converting enzyme inhibitor; ARB, angiotensin II receptor blockers; ACEI/ARB, angiotensin-converting enzyme inhibitor or angiotensin II receptor blockers; ARs, adverse reactions; BUN, blood urea nitrogen; DN, diabetic nephropathy; DC, Danshen-Chuanxiongqin injection; DH, Danhong injection; GG, Gegensu injection; HQ, Huangqi injection; KD, Kudiezi injection; MD, mean difference; OR, odds ratio; TC, total cholesterol; TG, triglyceride; DS, Danshen injection; RCTs, randomised controlled trials; SBP, systolic blood pressure; Scr, serum creatinine; SK, Shenkang injection; ST, Shuxuetong injection; SX, Shuxuening injection; SUCRA, surface under the cumulative ranking curve; TCM, traditional Chinese medicine; TCMI, traditional Chinese medicine injection; TER, total effective rate; UAER, urinary albumin excretion rate; YX, Yinxingdamo injection; 95% CI, 95% confidence interval; β_2_-MG, β_2_-microglobulin.
