# Supplementary material for: Comparative effectiveness of traditional Chinese medicine injections combined with ACEI/ARB for diabetic nephropathy: A systematic review and network meta-analysis
Source: Front Pharmacol. 2025 Jul 18;16:1543275. doi: 10.3389/fphar.2025.1543275 (PMC12313559; doi:10.3389/fphar.2025.1543275)
Supplement: Supplementary file 1 [file Supplementaryfile1.docx]

**Appendix**

[Appendix Table A.1: PRISMA_2020_checklist 3](#_Toc29242)

[Appendix Table A.2: Extract and extraction process description of TCMIs 5](#_Toc26396)

[Appendix Table A.3: More details about each TCMI 21](#_Toc32535)

[Appendix Table A.4-A.13: Search Strategy 25](#_Toc624)

[Table A.4: Search Strategy: PubMed 25](#_Toc18681)

[Table A.5: Search Strategy: Web of Science 26](#_Toc11349)

[Table A.6: Search Strategy: Scopus 27](#_Toc9031)

[Table A.7: Search Strategy: Embase 27](#_Toc18833)

[Table A.8: Search Strategy: Cochrane Library 28](#_Toc10951)

[Table A.9: Search Strategy: China National Knowledge Infrastructure (CNKI) 28](#_Toc27286)

[Table A.10: Search Strategy: the Chinese Scientific Journal database (VIP) 29](#_Toc8640)

[Table A.11: Search Strategy: Wanfang database 29](#_Toc30131)

[Table A.12: Search Strategy: SinoMed 29](#_Toc13820)

[Table A.13: Chinese Clinical Trial Registry (ChiCTR) 29](#_Toc1898)

[Appendix Table A.14: Specific intervention methods of the study 29](#_Toc18480)

[Appendix Table A.15: Basic information on the included studies 36](#_Toc25307)

[Appendix Table A.16: Risk of Bias of Included Studies 40](#_Toc18260)

[Appendix Table A.17-A.24: GRADE assessment 44](#_Toc23079)

[Table A.17: GRADE assessment: UAER 44](#_Toc2023)

[Table A.18: GRADE assessment: Scr 46](#_Toc5934)

[Table A.19: GRADE assessment: BUN 48](#_Toc32314)

[Table A.20: GRADE assessment: β](#_Toc12065)_[2](#_Toc12065)_[-MG 51](#_Toc12065)

[Table A.21: GRADE assessment: TC 53](#_Toc15417)

[Table A.22: GRADE assessment: TG 55](#_Toc32699)

[Table A.23: GRADE assessment: SBP 57](#_Toc22086)

[Table A.24: GRADE assessment: TER 58](#_Toc2845)

[Appendix Table A.25: SUCRA values 60](#_Toc12323)

[Appendix Table A.26: Adverse reaction 60](#_Toc28572)

[Appendix Table A.27: Egger’s regression test 61](#_Toc31236)

[Appendix Table A.28-A.35: Heterogeneity analysis 62](#_Toc17161)

[Table A.28: Heterogeneity analysis: UAER 62](#_Toc10034)

[Table A.29: Heterogeneity analysis: Scr 63](#_Toc5437)

[Table A.30: Heterogeneity analysis: BUN 65](#_Toc13047)

[Table A.31: Heterogeneity analysis: β](#_Toc15338)_[2](#_Toc15338)_[-MG 67](#_Toc15338)

[Table A.32: Heterogeneity analysis: TC 68](#_Toc28065)

[Table A.33: Heterogeneity analysis: TG 68](#_Toc23035)

[Table A.34: Heterogeneity analysis: SBP 69](#_Toc6877)

[Table A.35: Heterogeneity analysis: TER 69](#_Toc26389)

[Appendix Table A.36-A.38: Subgroup analysis 70](#_Toc13249)

[Table A.36: Subgroup analysis: UAER 70](#_Toc24561)

[Table A.37: Subgroup analysis: Scr 71](#_Toc29237)

[Table A.38: Subgroup analysis: SUCRA values 71](#_Toc20130)

[Appendix Table A.39-A.40: Sensitivity analysis 72](#_Toc14494)

[Table A.39: Sensitivity analysis: League table 72](#_Toc4622)

[Table A.40: Sensitivity analysis: SUCRA values 72](#_Toc16383)

[Appendix Fig A.1: INPLASY Protocol 72](#_Toc17950)

[Appendix Fig A.2: Funnel plots 73](#_Toc2016)

**Abbreviation explanation of** **traditional Chinese medicine injections (TCMIs) in this appendix:**

DC, Danshen-chuanxiongqin injection; DH, Danhong injection; GG, Gegensu injection; HQ, Huangqi injection; KD, Kudiezi injection; DS, Danshen injection; SK, Shenkang injection; ST, Shuxuetong injection; SX, Shuxuening injection; YX, Yinxingdamo injection.

Appendix Table A.1: PRISMA_2020_checklist

| **Section and Topic** | **Item #** | **Checklist item** | Location where item is reported |
| --- | --- | --- | --- |
| **TITLE** | | |  |
| Title | 1 | Identify the report as a systematic review. | Title |
| **ABSTRACT** | | |  |
| Abstract | 2 | See the PRISMA 2020 for Abstracts checklist. | Abstract |
| **INTRODUCTION** | | |  |
| Rationale | 3 | Describe the rationale for the review in the context of existing knowledge. | Introduction |
| Objectives | 4 | Provide an explicit statement of the objective(s) or question(s) the review addresses. | Introduction |
| **METHODS** | | |  |
| Eligibility criteria | 5 | Specify the inclusion and exclusion criteria for the review and how studies were grouped for the syntheses. | Inclusion criteria; Exclusion criteria |
| Information sources | 6 | Specify all databases, registers, websites, organisations, reference lists and other sources searched or consulted to identify studies. Specify the date when each source was last searched or consulted. | Materials and methods; Search strategy |
| Search strategy | 7 | Present the full search strategies for all databases, registers and websites, including any filters and limits used. | Search strategy |
| Selection process | 8 | Specify the methods used to decide whether a study met the inclusion criteria of the review, including how many reviewers screened each record and each report retrieved, whether they worked independently, and if applicable, details of automation tools used in the process. | Study selection process and data extraction |
| Data collection process | 9 | Specify the methods used to collect data from reports, including how many reviewers collected data from each report, whether they worked independently, any processes for obtaining or confirming data from study investigators, and if applicable, details of automation tools used in the process. | Study selection process and data extraction |
| Data items | 10a | List and define all outcomes for which data were sought. Specify whether all results that were compatible with each outcome domain in each study were sought (e.g. for all measures, time points, analyses), and if not, the methods used to decide which results to collect. | Study selection process and data extraction |
|  | 10b | List and define all other variables for which data were sought (e.g. participant and intervention characteristics, funding sources). Describe any assumptions made about any missing or unclear information. | Study selection process and data extraction; Inclusion criteria;Exclusion criteria |
| Study risk of bias assessment | 11 | Specify the methods used to assess risk of bias in the included studies, including details of the tool(s) used, how many reviewers assessed each study and whether they worked independently, and if applicable, details of automation tools used in the process. | Risk of bias assessment |
| Effect measures | 12 | Specify for each outcome the effect measure(s) (e.g. risk ratio, mean difference) used in the synthesis or presentation of results. | Statistical analysis |
| Synthesis methods | 13a | Describe the processes used to decide which studies were eligible for each synthesis (e.g. tabulating the study intervention characteristics and comparing against the planned groups for each synthesis (item #5)). | Inclusion criteria;Exclusion criteria; Study selection process and data extraction |
|  | 13b | Describe any methods required to prepare the data for presentation or synthesis, such as handling of missing summary statistics, or data conversions. | Study selection process and data extraction |
|  | 13c | Describe any methods used to tabulate or visually display results of individual studies and syntheses. | Statistical analysis |
|  | 13d | Describe any methods used to synthesize results and provide a rationale for the choice(s). If meta-analysis was performed, describe the model(s), method(s) to identify the presence and extent of statistical heterogeneity, and software package(s) used. | Statistical analysis |
|  | 13e | Describe any methods used to explore possible causes of heterogeneity among study results (e.g. subgroup analysis, meta-regression). | Statistical analysis |
|  | 13f | Describe any sensitivity analyses conducted to assess robustness of the synthesized results. | Statistical analysis |
| Reporting bias assessment | 14 | Describe any methods used to assess risk of bias due to missing results in a synthesis (arising from reporting biases). | Statistical analysis; Risk of bias assessment |
| Certainty assessment | 15 | Describe any methods used to assess certainty (or confidence) in the body of evidence for an outcome. | Statistical analysis; Risk of bias assessment |
| **RESULTS** | | |  |
| Study selection | 16a | Describe the results of the search and selection process, from the number of records identified in the search to the number of studies included in the review, ideally using a flow diagram. | Literature selection and study characteristics |
|  | 16b | Cite studies that might appear to meet the inclusion criteria, but which were excluded, and explain why they were excluded. | Literature selection and study characteristics |
| Study characteristics | 17 | Cite each included study and present its characteristics. | Literature selection and study characteristics |
| Risk of bias in studies | 18 | Present assessments of risk of bias for each included study. | Quality assessment of evidence |
| Results of individual studies | 19 | For all outcomes, present, for each study: (a) summary statistics for each group (where appropriate) and (b) an effect estimate and its precision (e.g. confidence/credible interval), ideally using structured tables or plots. | Network meta-analysis |
| Results of syntheses | 20a | For each synthesis, briefly summarise the characteristics and risk of bias among contributing studies. | Cluster analysis; Publication bias |
|  | 20b | Present results of all statistical syntheses conducted. If meta-analysis was done, present for each the summary estimate and its precision (e.g. confidence/credible interval) and measures of statistical heterogeneity. If comparing groups, describe the direction of the effect. | Network meta-analysis |
|  | 20c | Present results of all investigations of possible causes of heterogeneity among study results. | Tests of inconsistency and heterogeneity; Subgroup analysis |
|  | 20d | Present results of all sensitivity analyses conducted to assess the robustness of the synthesized results. | Tests of inconsistency and heterogeneity; Subgroup analysis; Sensitivity analysis |
| Reporting biases | 21 | Present assessments of risk of bias due to missing results (arising from reporting biases) for each synthesis assessed. | Cluster analysis; Publication bias |
| Certainty of evidence | 22 | Present assessments of certainty (or confidence) in the body of evidence for each outcome assessed. | Tests of inconsistency and heterogeneity; Subgroup analysis; Sensitivity analysis |
| **DISCUSSION** | | |  |
| Discussion | 23a | Provide a general interpretation of the results in the context of other evidence. | Main findings |
|  | 23b | Discuss any limitations of the evidence included in the review. | Main findings |
|  | 23c | Discuss any limitations of the review processes used. | Main findings; Limitations of the study; |
|  | 23d | Discuss implications of the results for practice, policy, and future research. | Reflection on Study Design |
| **OTHER INFORMATION** | | |  |
| Registration and protocol | 24a | Provide registration information for the review, including register name and registration number, or state that the review was not registered. | Materials and methods |
|  | 24b | Indicate where the review protocol can be accessed, or state that a protocol was not prepared. | Materials and methods |
|  | 24c | Describe and explain any amendments to information provided at registration or in the protocol. | Materials and methods |
| Support | 25 | Describe sources of financial or non-financial support for the review, and the role of the funders or sponsors in the review. | Funding |
| Competing interests | 26 | Declare any competing interests of review authors. | Conflict of Interest |
| Availability of data, code and other materials | 27 | Report which of the following are publicly available and where they can be found: template data collection forms; data extracted from included studies; data used for all analyses; analytic code; any other materials used in the review. | Supplementary Material |

Appendix Table A.2: Extract and extraction process description of TCMIs

**1. Related regulations**

| **Sterility Assurance in Manufacturing** | | | |
| --- | --- | --- | --- |
| Terminal Sterilization | Moist Heat Sterilization | **Primary Method**: Steam sterilization at 121°C for 15–30 minutes (minimum F₀≥12) to achieve a sterility assurance level (SAL) ≤10⁻⁶.  Validation Requirements:  Thermal Mapping: Ensure uniform heat distribution in the sterilization chamber.  Bioburden Monitoring: Pre-sterilization bioburden must be ≤100 CFU/mL, with validation of microbial resistance (D-value).  **Applicability**: Suitable for thermally stable formulations (e.g., solutions with pH 3–10). | |
|  | **Alternative Methods** | **Radiation Sterilization**: Gamma irradiation (dose: 25–50 kGy) for heat-sensitive products.  Validation: Requires dose mapping and microbial validation (e.g., Bacillus pumilus spores as biological indicators).  **Ethylene Oxide (EO) Sterilization**: Limited use for heat- and radiation-sensitive products, with strict residual gas control (EO ≤1 ppm; ethylene chlorohydrin ≤2 ppm). | |
| **Filtration Sterilization** | **Process Design** | **Filter Selection**: Use 0.22 μm (or smaller) sterilizing-grade membranes (e.g., PVDF, PES) validated for bacterial retention.  **Prefiltration**: Remove particles >5 μm to protect the sterilizing filter. | |
|  | **Validation and Testing** | **Bacterial Retention Test**: Validate using Brevundimonas diminuta (ATCC 19146) at ≥10⁷ CFU/cm².  **Integrity Testing**:  Bubble Point Test: Minimum pressure ≥0.3 MPa (for 0.22 μm filters).  Diffusion Flow Test: ≤10 mL/min·cm² under specified pressure. | |
|  | **Aseptic Processing** | **Environmental Controls**: ISO Class 5 cleanrooms for filling lines, with frequent monitoring of airborne particles (<3,520 particles/m³ for ≥0.5 μm) and microbial counts (NMPA GMP Annex 1: Sterile Products).  **Personnel Training**: Operators must follow aseptic gowning protocols and undergo regular microbiological monitoring. | |
| **Comprehensive Quality Control Measures** | | | |
| **Microbial Limits Testing** | **Acceptance Criteria** | | **Pre-sterilization**: Total aerobic microbial count (TAMC) ≤100 CFU/mL; total yeast/mold count (TYMC) ≤10 CFU/mL.  **Post-sterilization**: Sterility confirmed by membrane filtration or direct inoculation (no growth after 14 days). |
|  | **Methods** | | **Membrane Filtration**: Filter 100 mL of sample through 0.45 μm membranes, incubate in fluid thioglycollate medium (30–35°C) and soybean-casein digest medium (20–25°C).  **Direct Inoculation**: For viscous or insoluble products, inoculate directly into culture media. |
| **Endotoxin Testing** | **Limits** | | **General Limit**: ≤0.5 EU/mL for most injections.  **Dose-Dependent Limit**: Calculated as K/M, where K=5.0 EU/kg/h and M=maximum single dose (mL/kg/h). |
|  | **Testing Methods** | | **Gel-Clot LAL**: Qualitative method with sensitivity 0.03–1.0 EU/mL.  **Photometric LAL**: Quantitative turbidimetric or chromogenic assays (e.g., kinetic chromogenic method). |
| **Heavy Metal Residues** | **Permissible Limits** | | Lead (Pb) ≤5 ppm, Cadmium (Cd) ≤0.3 ppm, Arsenic (As) ≤2 ppm, Mercury (Hg) ≤0.2 ppm.  Copper (Cu) and Chromium (Cr) ≤10 ppm (if specified in monographs). |
|  | **Analytical Methods** | | **ICP-MS**: For multi-element detection with detection limits ≤0.01 ppm.  **Atomic Absorption Spectrometry (AAS)**: Single-element analysis with validation of recovery rates (85–115%). |
| **Impurity Control** | **Impurity Control** | | **Proteins: Tested by Bradford assay or SDS-PAGE; limit ≤0.05 mg/mL.**  **Tannins: Colorimetric assay (ferric chloride method); limit ≤0.1 mg/mL.**  **Resins: HPLC-UV detection; limit ≤0.05 mg/mL.** |
|  | **Process-Related Impurities** | | **Solvent Residues: Ethanol ≤0.5%, acetone ≤0.1% (GC-FID analysis).** |
| **Particulate Matter** | **Visible Particles** | | **None detected in 20 containers (manual inspection under light).** |
|  | **Subvisible Particles** | | **≤6,000 particles ≥10 μm and ≤600 particles ≥25 μm per container (light obscuration particle counter).** |
| **Regulatory Compliance** | | | |
| **Documentation and Validation** | **Botanical Origin Verification:**  **DNA barcoding or HPLC fingerprinting to confirm plant species.**  **Process Validation:**  **Sterilization, filtration, and aseptic processes must undergo three consecutive successful batches.** | | |
| **Batch Release Testing** | **Mandatory Tests: Sterility, endotoxins, heavy metals, pH (4.0–7.0), visible/subvisible particles, and active ingredient content (95–105% of labeled claim).** | | |
| **Stability Studies** | **Accelerated Testing: 40°C ± 2°C / 75% RH ± 5% for 6 months.**  **Long-Term Testing: 25°C ± 2°C / 60% RH ± 5% for 24 months.**  **Parameters Monitored: Physical stability, pH, microbial limits, and degradation products.** | | |
| **Implementation standards** | | | |
| HQ | **China National Medical Products Administration (NMPA) National Drug Standard: WS3-B-3335-98** | | |
| YX | **National Drug Standard of China (Elevated from Local Standards) – Chemical Drugs, Volume 1: WS-10001-(HD-0087)-2002** | | |
| **SX** | **China National Medical Products Administration (NMPA) National Drug Standard: WS3-B-3707-98-2004-2012** | | |
| DC | **China National Medical Products Administration (NMPA) National Drug Standard: WS-10001-（HD-1138）-2002-2017** | | |
| DS | **China National Medical Products Administration (NMPA) National Drug Standard: WS3-B-3766-98-2011** | | |
| DH | **China National Medical Products Administration (NMPA) National Drug Standard: WS-11220（ZD-1220）-2002** | | |
| SK | **National Drug Standard (Provisional) of the China National Medical Products Administration (NMPA): YBZ08522004** | | |
| KD | **National Traditional Chinese Medicine (TCM) Standards Compilation – Internal Medicine, Cardiology System Volume: WS-10354 (ZD-0354)-2002** | | |
| ST | **China National Medical Products Administration (NMPA) National Drug Standard:WS3-548（Z-048）-2005（Z）** | | |
| GG | **Pharmacopoeia of the People's Republic of China (2005 Edition), Part II: WS3-B-3707-98-2004-2012** | | |

Reference:

Chinese Pharmacopoeia (2020 Edition), General Chapters.

National Medical Products Administration (NMPA). Quality Control Standards for Traditional Chinese Medicine Injections. 2017.

NMPA. Technical Requirements for Re-evaluation of TCM Injection Safety. 2018.

NMPA. Guidelines for Sterilizing Filtration Technologies and Applications. 2018.

NMPA. Good Manufacturing Practice (GMP) Annex 1: Sterile Products. 2020.

**2. Basic Processes**

| Injection | Basic Processes |
| --- | --- |
| HQ | 1. Primary Aqueous Extraction  (1) The crude drug was decocted with water for multiple cycles.  (2) The combined decoction was filtered, concentrated, and subjected to ethanol precipitation.  (3) The mixture was refrigerated, filtered, and the ethanol was removed.  (4) The concentrate was diluted with water for injection and refrigerated.  2. Residue Ethanol Extraction  (1) The retained residue underwent reflux extraction with ethanol for multiple cycles.  The ethanol extract was concentrated, adjusted with water for injection, and refrigerated.  3. Combined Processing  Aqueous and ethanol extracts were pooled, adjusted with ethanol, and refrigerated.  Post-filtration, ethanol was removed, and the solution was concentrated.  Notes: Water for injection was used as the solvent throughout. |
| YX | 1. Ginkgo Leaf Extract Preparation  (1) Primary Extraction  Ginkgo leaves undergo reflux extraction with purified water. The filtrates are combined, concentrated to an appropriate volume, cooled, and filtered.  (2) Resin Purification  The concentrate is purified via macroporous resin adsorption, eluted with ethanol, and concentrated to remove solvents. The residue is redissolved in purified water, cooled, filtered, and vacuum-dried.  (3) Secondary Purification  The dried extract is treated with ethanol, centrifuged, concentrated, mixed with purified water, and vacuum-dried again.  2. Solution Formulation  (1) Extract Solution Preparation  The dried extract is homogenized with purified water under heating. Dipyridamole is added, and the pH is adjusted. The solution undergoes reflux, cooling, pH readjustment, followed by filtration and ultrafiltration.  (2) Sterilization  The ultrafiltered solution is sterilized under high-temperature conditions.  3. Component Preparation  (1) Solution A  Dipyridamole is dissolved in a mixture of propylene glycol and water for injection.  (2) Solution B  Tween 80 and vitamin C are dissolved in water for injection.  (3) Mixing Process  Solutions A and B are combined, treated with activated carbon for decolorization, and filtered.  4. Final Product Preparation  (1) Blending  The sterilized extract solution is mixed with the component solutions, and the pH is adjusted to the target range.  (2) Filtration and Filling  The blended solution is filtered through dual membranes and filled into ampoules according to specified volumes and excipient ratios.  (3) Terminal Sterilization  Filled ampoules undergo high-temperature pretreatment and terminal sterilization. |
| SX | The injection is manufactured through integrated extraction, purification, and formulation processes.  1. Raw Material Processing & Extraction  (1) Primary Extraction: Finely crushed leaves undergo ultrasonic extraction with an acidic solution.  (2) Sequential Reflux Extractions:  - First residue is refluxed with ethanol.  - Second residue is treated with a mixed solvent system.  (3) Post-extraction: Combined extracts are neutralized, allowed to settle, diluted, and acidified.  2. Purification & Chromatography  (1) Centrifugation: Performed to remove particulate matter.  (2) Resin Column Processing:  - Purification using a mixed-resin column.  - Sequential elution with purified water and ethanol solutions of increasing concentration.  3. Composite Preparation  (1) Solvent Extraction: Conducted twice with a specific solvent mixture under controlled conditions.  (2) Concentration: Organic phase is evaporated, dissolved in anhydrous ethanol, pH-adjusted, and cooled.  (3) Final Processing: Filtered, concentrated, and dried.  4. Final Formulation  (1) Solution Preparation: Composite dissolved in water for injection, filtered, and pH-adjusted.  (2) Decolorization: Treated with activated charcoal under heating.  (3) Sterilization: Cooled, pH-adjusted, diluted to target volume, homogenized, and terminally sterilized. |
| DC | The injection is prepared through sequential extraction, purification, and formulation processes.  1. Salvia Miltiorrhiza Extract Preparation  (1) Decoction: Raw material is decocted multiple times with water. Combined decoctions are filtered and concentrated.  (2) Acid-Base Precipitation: Treated with alkaline and acidic solutions for sedimentation, followed by filtration.  (3) Ethanol Processing: Ethanol precipitation is performed twice, with supernatant concentration, dilution, refrigeration, and re-concentration.  2. Final Extract Standardization  (1) Adjusted to a standardized concentration with water for injection.  (2) Clarification: Homogenized, refrigerated, filtered, and concentrated.  (3) Activated Charcoal Treatment: Treated with activated charcoal under heating, followed by coarse and membrane filtration.  3. Formulation with Tetramethylpyrazine  (1) Component Preparation: Tetramethylpyrazine HCl is dissolved in water, and glycerol is added.  (2) Mixing: Salvia extract is blended with the tetramethylpyrazine-glycerol solution and diluted to the target concentration.  (3) pH Adjustment: Stabilized to a defined pH range using acid/base solutions.  4. Sterilization & Packaging  (1) Final membrane filtration.  (2) Filled into ampoules under aseptic conditions.  (3) Terminal sterilization is performed post-filling. |
| DS | The injection is prepared through sequential extraction, ethanol precipitation, and sterilization processes.  1.Raw Material Processing  (1) Decoction: Crude herb undergoes multiple cycles of water decoction.  (2) Filtration & Concentration: Combined filtrates are sieved and concentrated under controlled temperature.  2.Ethanol Precipitation  (1) Primary Precipitation: Ethanol is added to achieve target concentration, followed by refrigeration, filtration, and solvent recovery.  (2) Secondary Precipitation: Further ethanol adjustment under alkaline conditions, refrigeration, filtration, and concentration.  3.Final Purification  (1) Dilution & pH Adjustment: Reconstituted with water for injection, pH-adjusted, refrigerated, and ultrafiltered.  (2) Activated Carbon Treatment: Heated with activated carbon, filtered, and pH-adjusted.  4.Formulation & Sterilization  (1) Volume Standardization: Diluted to target volume with water for injection.  (2) Filling: Dispensed into ampoules under aseptic conditions.  (3) Terminal Sterilization: Sealed ampoules undergo validated sterilization. |
| DH | 1. Preparation of Extraction Liquid A (Salvia miltiorrhiza Extract)  (1) Decoction pieces are soaked in preheated ethanol.  (2) Percolation is performed using heated ethanol, followed by ethanol recovery, gelatin precipitation, and filtration.  (3) The filtrate is concentrated to a clear paste, membrane-filtered, and refrigerated.  2. Preparation of Extraction Liquid B (Safflower Extract)  (1) Dried safflower undergoes dual hot water extraction.  (2) Combined filtrates are concentrated to a clear paste, membrane-filtered, and refrigerated.  3. Formulation & Sterilization  (1) Extracts A and B are mixed with injection-grade water and NaCl.  (2) pH is adjusted, and the solution is diluted to the target volume.  (3) Membrane-filtered, aseptically filled into vials, and terminally sterilized. |
| SK | The injection is prepared through parallel processing of two herbal groups, followed by combined purification and sterilization.  1.Herbal Group 1 Processing (Rheum officinale & Salvia miltiorrhiza)  (1) Decoction: Herbs undergo multiple cycles of water decoction.  (2) Ethanol Precipitation: Sequential ethanol precipitation steps with increasing concentrations, including gelatin treatment for impurity removal.  (3) Final Adjustments: Ethanol removal, dilution with water for injection, refrigeration, and filtration.  2.Herbal Group 2 Processing (Astragalus membranaceus & Carthamus tinctorius)  (1) Decoction: Processed using the same decoction protocol as Group 1.  (2) Ethanol Precipitation: Identical three-step ethanol precipitation sequence.  (3) Final Adjustments: Ethanol removal, dilution, refrigeration, and filtration.  3.Combined Formulation  (1) Mixing & Purification: Combined filtrates treated with activated carbon under heating, refrigerated, and filtered.  (2) Standardization: Diluted to target concentration, pH-adjusted, and volume-calibrated.  4.Sterilization & Packaging  (1) Filtration: Multi-stage membrane filtration.  (2) Filling: Aseptically dispensed into ampoules.  (3) Sterilization: Validated steam sterilization.  (4) Quality Control: Visual inspection prior to final release. |
| KD | The injection is prepared through sequential extraction, purification, and sterilization processes.  1.Decoction & Concentration  Raw herb undergoes dual water decoction cycles.  Combined decoctions are concentrated to a standardized crude drug concentration.  2.Alkali-Acid Precipitation  (1) Alkaline Treatment: Concentrate is treated with an alkaline solution, settled, and centrifuged.  (2) Acidic Ethanol Processing: Precipitate is suspended in ethanol, acidified, neutralized, and filtered.  3.Purification & Ultrafiltration  (1) Ethanol Removal: Solvent is evaporated, and residue is reconstituted with water for injection.  (2) Activated Carbon Treatment: Treated with activated carbon under heating, refrigerated, and filtered.  (3) Ultrafiltration: Concentrate undergoes heat sterilization and sequential membrane ultrafiltration.  4.Final Sterilization  (1) Formulation: Ultrafiltrate is pH-adjusted and diluted to target volume.  (2) Sterilization: Membrane-filtered, filled into containers, and terminally sterilized. |
| ST | 1.Raw Material Pretreatment  Hirudo and Pheretima are mixed, hydrated in physiological saline, rinsed, and homogenized.  2.Extraction & Concentration  (1) Double Extraction: Homogenate undergoes dual cycles of physiological saline extraction. Combined extracts are concentrated.  (2) Ethanol Precipitation: Ethanol is added to the concentrate, chilled, filtered, and solvent recovered.  3.Purification  (1) Decolorization: Filtrate is treated with activated carbon and filtered.  (2) Ultrafiltration: Purified via molecular weight cut-off membrane ultrafiltration.  4.Final Formulation  (1) Standardization: Adjusted to target volume with water for injection.  (2) Sterilization: Membrane-filtered, aseptically filled into vials, and terminally sterilized. |
| GG | The injection is formulated through sequential mixing, pH adjustment, and terminal sterilization.  1.Formulation  (1) Composition: Contains puerarin, propylene glycol, glutamic acid, Tween 80, ethanol, and water for injection per unit volume.  2.Mixing & Incubation  Puerarin and ethanol are mixed to form a uniform suspension and incubated under controlled temperature.  Solution Preparation  (1) Component Addition: Propylene glycol, glutamic acid, Tween 80, and a portion of water are added to the suspension and stirred until dissolution.  (2) Volume Adjustment: Diluted to the target volume with water for injection and adjusted to the target pH range.  3.Sterilization & Packaging  (1) Filtration: Sterilized through dual membrane filtration.  (2) Filling: Aseptically filled into ampoules under inert atmosphere.  (3) Terminal Sterilization: Validated steam sterilization process. |

Reference:

Chen, Y.X., and Wang, X.D. A preparation method of bitter saucer injection.

Chen, Z.C., and Yang, G.D. A kind of preparation method of blood-sparing injections.

Fang, T.H., Fan, Y.Q., Zhou, G.H., Jia, W.J., and Cui, Y.H. A kind of shu haining injection prepared from ginkgo biloba composition and its preparation method.

Guo, D.Y. A preparation method of Danhong injection and its products.

Li, X.M., Zhao, Y.X., Jiang, G.Z., and Sun, S.B. A refining method of high stability astragalus injection and astragalus injection.

Li, Y.H., Xu, Z.Y., Chai, J.G., Shen, P.Q., Zhang, J.B., Wang, M.L., et al. A preparation method of Salvia divinorum injection and its quality control method.

Wu, F., Lv, Y.Y., Cao, F.J., Wang, G., Wu, H.J., He, B.S., et al. Preparation method of Renkang injection.

Zhu, Y.X. A pharmaceutical preparation for the treatment of cardiovascular and cerebrovascular diseases and its preparation method.

**3. Identification**

| Injection | Identification |
| --- | --- |
| HQ | Identification of Astragaloside IV by Thin-Layer Chromatography (TLC):  A sample solution was prepared by dissolving 1.5 mL of the test substance in 30 mL of water, followed by extraction with water-saturated n-butanol (20 mL × 2). The combined n-butanol layers were washed with water (20 mL × 2), evaporated to dryness, and reconstituted in 0.5 mL of methanol. A reference solution was prepared by dissolving astragaloside IV in methanol (1 mg/mL). Both solutions (2 μL each) were spotted on a silica gel G plate and developed using the lower layer of a chloroform-methanol-water (13:7:2) mixture pre-equilibrated at <10°C. After drying, the plate was sprayed with 10% sulfuric acid-ethanol solution, heated at 105°C, and examined under daylight and UV light (365 nm). The test sample exhibited a brownish spot in daylight and an orange-yellow fluorescent spot under UV light, corresponding to the reference substance. |
| YX | 1.Flavonoid Color Reaction: A 2 mL sample treated with magnesium powder and concentrated hydrochloric acid developed a red color upon standing, characteristic of flavonoid compounds.  2.Fluorescence Reaction: A 2 mL sample mixed with ethanol (10 mL) exhibited green fluorescence, which disappeared after adding dilute hydrochloric acid, confirming pH-dependent fluorescence properties.  3.HPLC Retention Time Matching: In the HPLC chromatograms recorded for the assay of dipyridamole and Ginkgo total flavonoids, the retention time of the test sample peak corresponded to that of the reference standard peak, confirming chemical identity. |
| SX | Identification of Ginkgo biloba Extract by Thin-Layer Chromatography (TLC):  1.TLC with Ginkgo Leaf Reference Material:  (1) The test solution (prepared by extracting 30 mL sample with water-saturated n-butanol, evaporating, and dissolving in ethanol) and a reference solution (prepared from Ginkgo leaf crude drug) were spotted on a sodium carboxymethylcellulose-silica G plate.  (2) Developed with ethyl acetate–butanone–formic acid–water (5:3:1:1), visualized by spraying with 3% aluminum chloride ethanol solution.  (3) Under daylight and UV light (365/254 nm), the test sample showed spots identical in color and fluorescence to the reference material.  2.TLC for Ginkgolides A and B:  (1) Using the assay test solution (from ginkgolide A quantification) and reference standards (ginkgolides A/B in methanol), samples were chromatographed on the same plate type.  (2) Developed with toluene–ethyl acetate–acetone–methanol (5:2.5:2.5:0.3), treated with acetic anhydride vapor, heated at 160°C, and observed under UV (365 nm).  (3) The test sample displayed fluorescent spots matching the reference standards in position and color. |
| DC | 1.Ferric Chloride Color Reaction: A 1 mL sample treated with 2-3 drops of ferric chloride test solution exhibited a dirty green color, indicative of phenolic or tannin components.  2.HPLC Retention Time Confirmation: In the chromatogram recorded under the content determination method, the main peak of the test solution exhibited a retention time corresponding to that of the reference standard solution, confirming chemical identity. |
| DS | 1.UV-Vis Spectrophotometry: A 0.5 mL sample diluted to 500 mL with water exhibited maximum absorption at 281 ± 3 nm by spectrophotometric analysis.  2.Ferric Chloride Reaction: A mixture of 2 drops of the sample and 2 drops of water, treated with 1 drop of ferric chloride test solution, developed a dark green color, suggesting phenolic or tannin constituents.  3.TLC with Protocatechualdehyde Reference:  (1) The test solution (prepared by evaporating 4 mL of the sample, dissolving in anhydrous ethanol) and a reference solution (protocatechualdehyde in anhydrous ethanol, 1 mg/mL) were chromatographed on a silica gel G plate using benzene-ethyl acetate-formic acid (8:5:0.8) as the mobile phase.  (2) After spraying with a 2% ferric chloride–1% potassium ferricyanide solution (1:1), the test sample displayed a spot matching the reference standard in color and position. |
| DH | 1.UV-Vis Spectrophotometry: A 0.5 mL sample diluted to 500 mL with water exhibited maximum absorption at 281 ± 3 nm by spectrophotometric analysis, confirming characteristic UV absorption.  2.Ferric Chloride Reaction: Addition of 1 drop of ferric chloride test solution to a mixture of the sample (2 drops) and water (2 drops) produced a dark green coloration, indicative of phenolic or tannin-like constituents.  3.TLC with Protocatechualdehyde Reference:  (1) The test solution (prepared by evaporating 4 mL of the sample and dissolving the residue in anhydrous ethanol) and a reference solution (protocatechualdehyde in anhydrous ethanol, 1 mg/mL) were chromatographed on a silica gel G plate using benzene–ethyl acetate–formic acid (8:5:0.8) as the mobile phase.  (2) After derivatization with 2% ferric chloride–1% potassium ferricyanide solution (1:1), the test sample displayed a spot matching the reference standard in color and position, confirming the presence of protocatechualdehyde. |
| SK | NA |
| KD | Identification by Thin-Layer Chromatography (TLC):  The test solution was prepared by evaporating 20 mL of the sample and dissolving the residue in 50% ethanol. A reference solution containing adenosine (0.6 mg/mL in 50% ethanol) was used. Both solutions were spotted on a silica gel GF254 plate (prepared with 0.05 mol/L disodium hydrogen phosphate solution) and developed using chloroform–ethyl acetate–isopropanol–water–ammonia (8:2:6:0.4:0.6) as the mobile phase. After drying, the plate was examined under UV light (254 nm). The test sample exhibited a spot identical in color and position to the adenosine reference standard. |
| ST | 1.TLC with Hirudo Reference Material:  (1) The test solution (prepared by evaporating 2 mL of the sample and dissolving the residue in 50% ethanol) and a reference solution (prepared from Hirudo crude drug by grinding, sonication, and centrifugation) were chromatographed on a silica gel H plate with sodium carboxymethylcellulose binder.  (2) Developed using *n*-butanol–water–tetrahydrofuran (3.5:0.5:1.0), visualized by spraying with 0.2% ninhydrin ethanol solution and heating at 105°C.  (3) The test sample displayed a purplish-red spot at the same position as the reference material.  2. UV-Vis Spectrophotometry: A 0.2 mL sample diluted to 10 mL with water exhibited a maximum absorption at 252 ± 2 nm during scanning from 200 to 400 nm, confirming characteristic UV absorption. |
| GG | 1.Iron Complexation Reaction: A sample treated with 0.5% ferric chloride solution (2-3 drops) followed by 0.5% potassium ferricyanide solution (2-3 drops) developed a blue-green coloration, characteristic of phenolic or flavonoid components.  2.UV-Vis Spectrophotometry: A solution of the sample in ethanol (containing approximately 10 μg/mL of puerarin) exhibited a maximum absorption at 250 nm by ultraviolet-visible spectrophotometry (General Chapter 0401), confirming the presence of puerarin. |

Note：The above information is from China Pharmaceutical Information Query Platform (recognised by the State Drug Administration of China)

**4. Quality control tests for the pharmaceutical product**

| Injection | Quality control tests for the pharmaceutical product |
| --- | --- |
| HQ | 1.pH Value: Must be within 6.0-7.5 (Chinese Pharmacopoeia).  2.Pyrogen Test: Conducted via the pyrogen testing method (Chinese Pharmacopoeia) using a dosage of 1.60 mL per kg of rabbit body weight. Results must comply with regulatory standards.  3.Hemolysis Test:  (1) Preparation of 2% Red Blood Cell (RBC) Suspension: Rabbit cardiac blood is defibrinated, washed with physiological saline, and centrifuged until the supernatant is clear. The RBCs are diluted to a 2% suspension with physiological saline, prepared on the day of use.  (2) Procedure: Five test tubes are used:  Tubes 1-3: 0.3 mL test sample + 2.2 mL physiological saline.  Tube 4: 2.5 mL physiological saline (negative control).  Tube 5: 2.5 mL distilled water (positive control).  3.All tubes are mixed with 2.5 mL RBC suspension, incubated at 36.5±0.5°C for 3 hours, and observed for absence of hemolysis.  4.Additional Requirements: Must comply with all relevant specifications for injections (Chinese Pharmacopoeia). |
| YX | 1.pH Value: Must comply with 3.5–5.5 (Chinese Pharmacopoeia).  2.Heavy Metals: Accurately measure 2 mL of the product, evaporate to dryness in a crucible on a water bath, and test according to Method II (Chinese Pharmacopoeia). Heavy metal content shall not exceed 5 ppm.  3.Pyrogen Test: Dilute the product 1:1 with 10% glucose injection and test according to the pyrogen method (Chinese Pharmacopoeia). A dose of 3 mL diluted solution per kg of rabbit body weight is administered slowly. Results must meet regulatory requirements.  4.Additional Requirements: Conform to all relevant specifications for injections (Chinese Pharmacopoeia). |
| SX | 1. Total Flavonol Glycosides (Chinese Pharmacopoeia):  (1) Chromatographic Conditions: C18 column; mobile phase: methanol-0.4% phosphoric acid (55:45); detection wavelength: 368 nm. System suitability: theoretical plates ≥2500 (quercetin peak), resolution >1.5 (quercetin vs. isorhamnetin).  (2) Standard Solution: Accurately weighed quercetin, kaempferol, and isorhamnetin reference standards (dried over P₂O₅) dissolved in methanol (0.03 mg/mL, 0.03 mg/mL, and 0.02 mg/mL, respectively) or prepared from individual stock solutions.  (3) Test Solution: 10 mL sample + 16 mL methanol + 6 mL 18% HCl, refluxed at 85°C for 1.5 h, cooled, diluted to 50 mL with methanol, filtered (0.45 μm).  (4) Assay: Inject 10 μL of standard and test solutions. Calculate total flavonol glycosides as (quercetin + kaempferol + isorhamnetin) × 2.51.  (5) Specification: 90.0–110.0% of labeled claim.  2. Ginkgolide A (Chinese Pharmacopoeia):  (1) Chromatographic Conditions: C18 column; mobile phase: methanol-water (3:7); differential refractive index detector. System suitability: theoretical plates ≥2500 (ginkgolide A peak).  (2) Standard Solution: Accurately weighed ginkgolide A reference standard dissolved in methanol (1 mg/mL).  (3) Test Solution: 25 mL sample acidified to pH 2, extracted with ether (4×20 mL), washed with 5% NaCl (2×15 mL), ether layer evaporated, residue dissolved in methanol (2 mL).  (4) Assay: Inject 10 μL of standard and test solutions.  (5) Specification: ≥80.0% of labeled claim. |
| DC | 1.Danshensu (Salvianic Acid A) Assay (HPLC, Chinese Pharmacopoeia):  (1) Chromatographic Conditions: C18 column; mobile phase: methanol-0.2% glacial acetic acid (15:85); flow rate: 1.0 mL/min; detection wavelength: 280 nm. System suitability: theoretical plates ≥1500 (Danshensu peak).  (2) Standard Solution: Accurately weighed danshensu sodium reference standard dissolved in methanol to prepare ~90 mg/mL solution (equivalent to 0.875 mg/mL danshensu).  (3) Test Solution: 5–25 mL sample diluted with methanol to volume, filtered (0.45 μm).  (3) Assay: Inject 10 μL of standard and test solutions. Quantify via external standard method using peak area.  2. Ligustrazine Hydrochloride Assay (HPLC, Chinese Pharmacopoeia):  (1) Chromatographic Conditions: C18 column; mobile phase: methanol-0.4% phosphoric acid (10:90); flow rate: 1.0 mL/min; detection wavelength: 292 nm. System suitability: theoretical plates ≥2000 (ligustrazine peak).  (2) Standard Solution: Accurately weighed ligustrazine hydrochloride reference standard dissolved in methanol to prepare ~40 μg/mL solution.  (3) Test Solution: 1 mL sample diluted stepwise with methanol (1 mL → 50 mL → 10 mL), filtered (0.45 μm).  (4) Assay: Inject 5 μL of standard and test solutions. Quantify via external standard method using peak area.  3. Specification: The sterile aqueous solution contains danshensu in the range of 0.36–0.44 mg/mL. |
| DS | 1.pH Value: Must comply with 5.0–7.0 (Chinese Pharmacopoeia).  2.Pyrogen Test: Tested via the pyrogen method (Chinese Pharmacopoeia) using a dosage of 1.5 mL per kg of rabbit body weight. Results must meet regulatory standards.  3.Hemolysis and Aggregation Test:  (1) Preparation of 2% Red Blood Cell (RBC) Suspension: Rabbit cardiac blood is defibrinated, washed with physiological saline, and centrifuged until the supernatant is colorless. RBCs are diluted to a 2% suspension with physiological saline (prepared fresh and mixed before use).  (2) Procedure:  Tubes 1–3: 0.3 mL test sample + 2.2 mL physiological saline.  Tube 4: 2.5 mL physiological saline (negative control).  Tube 5: 2.5 mL distilled water (positive control).  (3) All tubes are mixed with 2.5 mL RBC suspension and incubated at 36.5 ± 0.5°C for 3 hours. No hemolysis or aggregation should occur. If aggregation is observed in the test sample tube, gentle shaking should uniformly disperse the aggregates.  (4) Additional Requirements: Conform to all specifications for injections (Chinese Pharmacopoeia). |
| DH | 1.pH Value: Must comply with 4.5–6.5 (Chinese Pharmacopoeia).  2.Protein Test: Add 1–3 drops of tannic acid solution to 1 mL of the product. No turbidityshould occur.  3.Residue on Ignition: Test 10 mL of the product according to Chinese Pharmacopoeia. Residue must not exceed 1.5% (g/mL).  4.Hemolysis Test:  (1) Preparation of 2% RBC Suspension: Defibrinate rabbit cardiac blood by shaking with glass beads for 10 minutes. Wash red blood cells (RBCs) with physiological sodium chloride solution until supernatant is colorless, then dilute to a 2% suspension (freshly prepared and mixed before use).  (2) Procedure:  Tubes 1–3: 0.3 mL test sample + 2.2 mL saline.  Tube 4: 2.5 mL saline (negative control).  Tube 5: 2.5 mL distilled water (positive control).  Add 2.5 mL RBC suspension to each tube, incubate at 36.5 ± 0.5°C for 3 hours. No hemolysis should occur.  5.Pyrogen Test: Conducted according to Chinese Pharmacopoeia. Inject 2 mL per kg of rabbit body weight. Results must meet standards.  6.Additional Requirements: Conform to all specifications for injections and related substance tests (Chinese Pharmacopoeia). |
| SK | NA |
| KD | 1.pH Value: Must comply with 5.5–7.2 (Chinese Pharmacopoeia).  2.Sulfate Test: Test 2 mL of the product according to Chinese Pharmacopoeia 2000. Results must not exceed the limit of 0.005% (compared to a reference solution prepared with 1 mL standard potassium sulfate solution).  3.Calcium Salt Test: Add 1 mL ammonium oxalate solution to 2 mL of the product, mix, and let stand for 10 minutes. No turbidity should occur.  4.Protein Test: Add 1–3 drops of tannic acid solution to 1 mL of the product. No turbidity should be observed.  5.Tannins Test: Mix 1 mL of the product with 5 mL of freshly prepared 1% egg white in saline. No turbidity or precipitate should form after standing for 10 minutes.  6.Resins Test: Extract 5 mL of the product with 10 mL chloroform. Evaporate the chloroform layer, dissolve the residue in 2 mL glacial acetic acid, add 3 mL water, and let stand for 30 minutes. No flocculent matter should precipitate.  7.Oxalate Test: Test 2 mL of the product according to Chinese Pharmacopoeia. No turbidity or precipitate is permitted.  8.Potassium Ion Test: Evaporate 2 mL of the product to dryness and test according to Chinese Pharmacopoeia. Results must not exceed the limit (compared to a reference solution prepared with 0.8 mL standard potassium solution).  9.Insoluble Particles: Mix 5 vials of the product with 500 mL of 5% glucose injection filtered through a 0.45 μm membrane. Test according to Chinese Pharmacopoeia. Results must comply with standards.  10.Bacterial Endotoxins: Test according to Chinese Pharmacopoeia Endotoxin content must be <6 EU/mL.  11.Additional Requirements: Conform to all specifications for injections (Chinese Pharmacopoeia). |
| ST | 1.pH Value: Must comply with 5.00–6.00 (Chinese Pharmacopoeia).  2.High-Molecular-Weight Substances (HPLC Method, Chinese Pharmacopoeia):  (1) Chromatographic Conditions: TSKGEL2000SWxl column (7.8 mm × 300 mm); mobile phase: trifluoroacetic acid-acetonitrile-water (0.025:30:70); detection wavelength: 214 nm; flow rate: 0.7 mL/min. System suitability: theoretical plates ≥5000 (insulin peak).  (2) Standard Solution: Human insulin (MW 5800) dissolved in mobile phase (0.1 mg/mL).  (3) Test Solution: Dilute 2 mL sample to 10 mL with mobile phase.  (4) Assay: Inject 20 μL of standard and test solutions. Peaks eluting before insulin (MW 5800) are considered high-MW impurities. Total area of these peaks must be ≤1.0% (area normalization method).  3.Relative Density: ≥1.005 (Chinese Pharmacopoeia).  4.Residue on Ignition: Test 2 mL of the product (Chinese Pharmacopoeia). Residue must not exceed 1.5% (g/mL).  5.Heavy Metals: Test 2 mL of the product (Chinese Pharmacopoeia). Content must be ≤10 ppm.  6.Arsenic: Test 0.4 mL of the product (Chinese Pharmacopoeia). Content must be ≤5 ppm.  7.Protein Test: Add 1.0 mL of 30% sulfosalicylic acid to 1 mL sample. No turbidity should occur.  8.Potassium Ion:  (1) Limit Test: Ash 2.0 mL sample, dissolve in 6% acetic acid, and compare turbidity with a reference (0.8 mL standard potassium solution). No greater turbidity is permitted.  (2) Quantitative Limit: Potassium ion content must be ≤1.0 mg/mL.  9.Pyrogen Test: Conducted via intravenous injection of 1.0 mL per kg rabbit body weight (Chinese Pharmacopoeia). Results must comply with standards.  10.Allergy Test: Performed according to Chinese Pharmacopoeia.  11.Abnormal Toxicity: Tested via intravenous injection (Chinese Pharmacopoeia). Results must meet requirements.  12.Hemolysis and Aggregation Test:  (1) 2% RBC Suspension: Defibrinate rabbit cardiac blood, wash with saline, and prepare a 2% suspension (freshly prepared and mixed).  (2) Procedure: Mix 2.5 mL RBC suspension with sample (0–0.5 mL) and saline (2.5–2.0 mL). Incubate at 37°C for 3 hours, observing every 15 minutes initially, then hourly. No hemolysis should occur in the 0.3 mL sample tube within 2 hours. Aggregates (if present) must disperse upon shaking or under microscopic examination.  13.Additional Requirements: Conform to all specifications for injections (Chinese Pharmacopoeia). |
| GG | 1.pH Value: Must comply with 3.5–5.5 (Chinese Pharmacopoeia).  2.Solution Color: Compare the product with Yellow Reference Solution No. 2 (Chinese Pharmacopoeia). The sample must not appear darker.  3.Related Substances (HPLC Method, Chinese Pharmacopoeia):  (1) Chromatographic Conditions: C18 column (4.6 mm × 250 mm, 5 μm); detection wavelength: 250 nm; mobile phase A: 0.1% citric acid, mobile phase B: methanol (gradient elution as specified).  (2) System Suitability: Peaks for puerarin (retention time ≈14 min) and caffeine must show resolution >4.0.  (3) Impurity Limits:  Impurity I (8-(β-D-glucofuranosyl)-4′,7-dihydroxyisoflavone) and Impurity II (8-(α-D-glucofuranosyl)-4′,7-dihydroxyisoflavone): Each ≤1.5% of the main peak area.  Other impurities: Individual ≤0.5%, total ≤3.0%.  4.Propylene Glycol (GC Method, Chinese Pharmacopoeia):  (1) Chromatographic Conditions: PEG-20M column; temperature program: 50°C to 220°C (30°C/min); detector at 280°C.  (2) Limit: ≤550 mg/mL.  5.Diethylene Glycol (GC Method, Chinese Pharmacopoeia):  (1) Chromatographic Conditions: Same as propylene glycol.  (2) Limit: ≤0.001%.  6.Abnormal Toxicity: Prepare a solution containing 10 mg/mL puerarin in saline (Chinese Pharmacopoeia). Results must comply with standards.  7.Pyrogen Test: Dilute 5 mL of the product to 10 mg/mL puerarin with pyrogen-free saline. Inject 5 mL/kg rabbit body weight (Chinese Pharmacopoeia).  8.Allergy Test: Conducted according to General Chapter 1147.  9.Hemolysis and Aggregation: Prepare a 20 mg/mL puerarin solution in saline (Chinese Pharmacopoeia). No hemolysis/aggregation permitted.  10.Sterility Test: Assessed via membrane filtration (Chinese Pharmacopoeia).  11.Additional Requirements: Conform to all specifications for injections (Chinese Pharmacopoeia). |

Note：The above information is from China Pharmaceutical Information Query Platform (recognised by the State Drug Administration of China)

**5. Assay**

| Injection | Assay |
| --- | --- |
| HQ | 1.Content Determination: The sample (10 ml) was evaporated to dryness on a water bath. The residue was dissolved in 2 ml of 1% sodium hydroxide solution and passed through a D101 macroporous adsorption resin column (1 cm × 12 cm). After sequential elution with 50 ml of 1% sodium hydroxide (discarded), water (neutral eluent, ~50 ml, discarded), 30% ethanol (50 ml, discarded), and 70% ethanol (50 ml collected), the collected 70% ethanol eluate was evaporated to dryness. The residue was dissolved in methanol and diluted to 2 ml as the test solution. A reference solution was prepared by dissolving astragaloside reference standard in methanol (1 mg/ml). Thin-layer chromatography (TLC) was performed on silica gel G plates using the lower layer of chloroform-ethyl acetate-methanol-water (15:40:22:10, stored below 10°C) as the developing system. After derivatization with 20% sulfuric acid ethanol solution and heating at 105°C, the plates were scanned at λs=395 nm and λR=700 nm for quantitative analysis by TLC scanning.  2.Specification: The preparation contains not less than 0.08 mg of astragaloside IV (C41H68O14) per 1 ml. |
| YX | 1.Dipyridamole Assay  (1) Content Determination: Quantified by HPLC (Chinese Pharmacopoeia).  (2) Chromatographic Conditions: Column: C18 (octadecylsilane-bonded silica); Mobile phase: 0.1% sodium dihydrogen phosphate (pH 4.6 adjusted with phosphoric acid)-methanol (25:75); Flow rate: 1.0 mL/min; Detection wavelength: 290 nm. System suitability: Theoretical plates ≥2000 (dipyridamole peak); RSD ≤2.0% for five replicate injections.  (3) Standard Solution: Accurately weighed dipyridamole reference standard dissolved in 80% methanol (15 mg/mL).  (4) Test Solution: 2 mL sample diluted to 50 mL with 80% methanol.  (5) Procedure: 20 μL of standard and test solutions injected. Quantification via external standard method using peak area.  2.Ginkgo Total Flavonoids Assay  (1) Content Determination: Analyzed by HPLC (Chinese Pharmacopoeia).  (2) Chromatographic Conditions: Column: C18; Mobile phase: 0.4% phosphoric acid-methanol (50:50); Flow rate: 1.2 mL/min; Detection wavelength: 360 nm. System suitability: Theoretical plates ≥1500 (quercetin peak); RSD ≤2.0% for five replicate injections.  (3) Standard Solution: Accurately weighed quercetin reference standard (12 mg) diluted with methanol to 200 mL.  (4) Test Solution: 25 mL sample hydrolyzed with 15 mL sulfuric acid (5% v/v) and 20 mL methanol under reflux (4 h), cooled, diluted to 100 mL with methanol, and filtered (0.45 mm membrane).  (5) Procedure: 20 μL of solutions injected. Total flavonoids calculated as Q×2.50 + K×2.59 + I×2.44, where Q (quercetin), K (kaempferol), and I (isorhamnetin) were quantified via external standard method. |
| SX | 1.Total Flavonol Glycosides Assay  (1) Content Determination: Analyzed by HPLC (Chinese Pharmacopoeia).  (2) Chromatographic Conditions: C18 column; mobile phase: methanol-0.4% phosphoric acid (55:45); detection wavelength: 368 nm. System suitability: Theoretical plates ≥2500 (quercetin peak); resolution ≥1.5 (quercetin vs. isorhamnetin).  (3) Standard Solution: Prepared by dissolving quercetin, kaempferol, and isorhamnetin reference standards (pre-dried over P2O5) in methanol to final concentrations of 0.03 mg/mL, 0.03 mg/mL, and 0.02 mg/mL, respectively (or prepared as individual stock solutions: quercetin 0.1 mg/mL, kaempferol 0.1 mg/mL, isorhamnetin 0.05 mg/mL; mixed before use).  (4) Test Solution: 10 mL sample hydrolyzed with 16 mL methanol and 6 mL 18% HCl under reflux (1.5 h), cooled, diluted to 50 mL with methanol, filtered (0.45 μm).  (5) Procedure: Injected 10 μL of solutions. Total flavonol glycosides calculated as (quercetin + kaempferol + isorhamnetin) × 2.51.  (6) Specification: Total flavonol glycosides must be 90.0–110.0% of the labeled claim.  2. Ginkgolide A Assay  (1) Content Determination: Analyzed by HPLC (Chinese Pharmacopoeia) with refractive index detection.  (2) Chromatographic Conditions: C18 column; mobile phase: methanol-water (30:70); theoretical plates ≥2500 (ginkgolide A peak).  (3) Standard Solution: Prepared by dissolving ginkgolide A reference standard in methanol (1 mg/mL).  (4) Test Solution: 25 mL sample acidified to pH 2 with dilute HCl, extracted with ethyl acetate (4 × 20 mL), washed with 5% NaCl (2 × 15 mL), back-extracted, dried, dissolved in methanol, and diluted to 2 mL.  (5) Procedure: Injected 10 μL of solutions.  (6) Specification: Ginkgolide A content ≥80% of the labeled claim. |
| DC | 1.Danshensu (Salvianic Acid A) Assay  (1) Content Determination: Analyzed by HPLC (Chinese Pharmacopoeia 2000 Edition, Part II, Appendix VD).  (2) Chromatographic Conditions: C18 column; mobile phase: methanol-0.2% glacial acetic acid (15:85); flow rate: 1.0 mL/min; detection wavelength: 280 nm. System suitability: Theoretical plates ≥1500 (Danshensu peak).  (3) Standard Solution: Prepared by dissolving danshensu sodium reference standard in methanol (90 μg/mL, equivalent to 78.75 μg/mL danshensu).  (4) Test Solution: 5–25 mL sample diluted with methanol to volume, filtered (0.45 μm membrane).  (5) Procedure: 10 μL of standard and test solutions injected. Quantified via external standard method using peak area.  2. Ligustrazine Hydrochloride Assay  (1) Content Determination: Analyzed by HPLC (Chinese Pharmacopoeia).  (3) Chromatographic Conditions: C18 column; mobile phase: methanol-0.4% phosphoric acid (10:90); flow rate: 1.0 mL/min; detection wavelength: 292 nm. System suitability: Theoretical plates ≥2000 (ligustrazine hydrochloride peak).  (4) Standard Solution: Prepared by dissolving ligustrazine hydrochloride reference standard in methanol (40 μg/mL).  (5) Test Solution: 1 mL sample diluted to 50 mL with methanol, further diluted 1 mL to 10 mL, filtered (0.45 μm membrane).  (6) Procedure: 5 μL of solutions injected. Quantified via external standard method using peak area.  3. Specification:  The sterile aqueous solution contains danshensu (calculated as free acid) in the range of 0.36–0.44 mg per 1 mL. |
| DS | Content Determination of Protocatechuic Aldehyde  Analytical Method: HPLC (Chinese Pharmacopoeia).  (1) Chromatographic Conditions: Column: octadecylsilane-bonded silica; mobile phase: methanol-0.2 mol/L ammonium acetate (pH 2.2 adjusted with sulfuric acid) (12:88); detection wavelength: 280 nm. System suitability: Theoretical plates ≥3000 (protocatechuic aldehyde peak).  (2) Standard Solution: Prepared by dissolving protocatechuic aldehyde reference standard in 5% methanol (0.02 mg/mL).  (3) Test Solution: 2 mL sample (from fill uniformity test) diluted to 25 mL with water.  (4) Procedure: Injected 10 μL of standard and test solutions. Quantification via external standard method.  (5) Specification: Contains not less than 0.2 mg of protocatechuic aldehyde (C7H6O3) per 1 mL. |
| DH | 1. Danshen (Salvia miltiorrhiza) Assay  Content Determination: Analyzed by HPLC (Chinese Pharmacopoeia).  (1) Chromatographic Conditions: C18 column; mobile phase: methanol-1% glacial acetic acid (13:87); detection wavelength: 280 nm. System suitability: Theoretical plates ≥5000 (danshensu peak).  (2) Standard Solutions: Prepared by dissolving danshensu sodium and protocatechuic aldehyde reference standards in water (50 μg/mL each).  (3) Test Solution: 5 mL sample diluted to 20 mL with water.  (4) Procedure: 10 μL of standard and test solutions injected. Quantified via external standard method.  (5) Specification: Contains not less than 0.5 mg/mL of total danshensu (C9H10O5) and protocatechuic aldehyde (C7H6O3).  2. Total Flavonoids Assay  Content Determination: Analyzed by spectrophotometric method (Chinese Pharmacopoeia).  (1) Standard Solution: Prepared by dissolving rutin reference standard (dried at 120°C) in 50% methanol (0.2 mg/mL anhydrous rutin).  (2) Calibration Curve: Prepared by reacting rutin standard solutions (0.2–1.0 mg/mL) with 5% NaNO2 (0.3 mL, 6 min), 10% Al(NO3)3 (0.3 mL, 6 min), and 4 mL NaOH, followed by dilution with 50% methanol. Absorbance measured at 500 nm.  (3) Test Solution: 5 mL sample diluted to 100 mL with water, further diluted 1 mL to 10 mL with 50% methanol.  (4) Procedure: Reaction performed as per calibration curve. Absorbance compared to blank (50% methanol).  (5) Specification: Contains not less than 5.0 mg/mL of total flavonoids (calculated as rutin, C27H30O16). |
| SK | NA |
| KD | 1. Total Flavonoids Assay  (1) Content Determination: Analyzed by spectrophotometric method (Chinese Pharmacopoeia).  Standard Solution: Prepared by dissolving rutin reference standard (dried to constant weight at 120°C under reduced pressure) in 60% ethanol (0.1 mg/mL, heated at 80°C if necessary).  (2) Test Solution: 10 mL sample (from fill uniformity test) diluted to 50 mL with 60% ethanol.  (3) Procedure: 5 mL of standard and test solutions reacted with 5% NaNO2 (0.3 mL, 6 min), 10% Al(NO3)3 (0.3 mL, 6 min), and 1 mol/L NaOH (4 mL), diluted to 10 mL with 60% ethanol. Absorbance measured at 505 nm against a blank (5 mL test solution diluted to 10 mL with 60% ethanol).  (4) Specification: Contains not less than 4.0 mg of total flavonoids (calculated as anhydrous rutin, C27H30O16) per 10 mL.  2. Adenosine Assay  (1) Content Determination: Analyzed by HPLC (Chinese Pharmacopoeia).  (2) Chromatographic Conditions: C18 column; mobile phase: acetonitrile-water (6:94); detection wavelength: 260 nm. System suitability: Theoretical plates ≥5000 (adenosine peak).  (3) Standard Solution: Prepared by dissolving adenosine reference standard in 10% methanol (35 μg/mL).  (4) Test Solution: 25 mL pooled sample (from 6 vials) extracted with chloroform (2 × 10 mL), aqueous layer evaporated to dryness, residue dissolved in 10% methanol, diluted to 5 mL, and filtered (0.45 μm).  (5) Procedure: Injected 10–15 μL of solutions. Quantified via external standard method.  (6) Specification: Contains not less than 0.025 mg of adenosine (C10H12N5O4) per 10 mL, derived from Ixeris sonchifolia. |
| ST | Content Determination  1. Total Solids  (1) Method: 20 mL sample evaporated to dryness in a pre-weighed evaporating dish, dried at 105°C to constant weight.  (2) Specification: Contains not less than 12.0 mg/mL of total solids.  2. Total Amino Acids and Peptide-Bound Amino Acids  Analytical Method: HPLC (Chinese Pharmacopoeia).  (1) Standard Solution: Prepared by dissolving amino acid reference standards (aspartic acid, glutamic acid, serine, histidine, glycine, threonine, alanine, arginine, tyrosine, cystine, valine, methionine, phenylalanine, isoleucine, leucine, lysine, proline) in pH 10.4 borate buffer, derivatized with o-phthalaldehyde (OPA) and 9-fluorenylmethyl chloroformate (FMOC-Cl).  (2) Test Solutions:  Total Amino Acids: 50 μL sample hydrolyzed with 6 mol/L HCl (containing 1% phenol) at 110°C for 22 h, neutralized, filtered, and derivatized.  Free Amino Acids: Directly derivatized without hydrolysis.  (3) Procedure: Injected 10 μL of solutions. Peptide-bound amino acids = Total amino acids – Free amino acids.  (4) Specification: Total amino acids ≥2.00 mg/mL; Peptide-bound amino acids ≥0.65 mg/mL.  3. Total Sugars and Polysaccharides  (1) Total Sugars:  Method: 2 mL sample hydrolyzed with 36 mL dilute HCl, neutralized, reacted with 0.01 mol/L iodine (25 mL), titrated with 0.01 mol/L sodium thiosulfate.  Calculation: 1 mL 0.01 mol/L iodine ≈ 0.9909 mg glucose (C6H12O6·H2O).  (2) Monosaccharides: Determined similarly without acid hydrolysis.  (3) Polysaccharides: Total sugars – Monosaccharides.  (4) Specification: Total sugars ≥6.00 mg/mL; Polysaccharides ≥0.90 mg/mL.  4. Hypoxanthine  Analytical Method: HPLC (Chinese Pharmacopoeia 2005 Edition, Part I, Appendix VID).  (1) Chromatographic Conditions: C18 column; mobile phase: 0.1% disodium hydrogen phosphate; detection wavelength: 254 nm. System suitability: Theoretical plates ≥3000 (hypoxanthine peak).  (2) Standard Solution: Hypoxanthine reference standard dissolved in water (0.1 mg/mL).  (3) Procedure: Injected 6 μL of solutions.  (4) Specification: Contains not less than 0.05 mg/mL of hypoxanthine. |
| GG | Content Determination of Puerarin  Analytical Method: HPLC (Chinese Pharmacopoeia).  1.Chromatographic Conditions: C18 column; mobile phase: 0.1% citric acid-methanol (75:25); detection 2.wavelength: 250 nm. System suitability: Theoretical plates ≥5000 (puerarin peak); resolution meets requirements.  3.Procedure: Accurately weighed sample dissolved in mobile phase to prepare test solution (50 μg/mL puerarin). Injected 10 μL into HPLC. Quantified via external standard method using peak area.  4.Specification: The sterile aqueous solution contains puerarin (C21H20O9) with solubilizing excipients. Puerarin content: 93.0–107.0% of the labeled claim. |

Note: The above information is from China Pharmaceutical Information Query Platform (recognised by the State Drug Administration of China)

**6. Pharmacological effects**

| Injection | Pharmacological interaction |
| --- | --- |
| HQ | It demonstrates cardioprotective effects through positive inotropic action, enhanced myocardial contractility, increased coronary blood flow, cardiomyocyte protection, and improved cardiovascular function. |
| YX | This preparation demonstrates dual mechanisms through:  1.Ginkgo total flavonoids - dilates coronary/cerebral vessels, alleviates cerebral ischemic symptoms, and improves memory function.  2.Dipyridamole - inhibits platelet aggregation via:  - Dose-dependent adenosine reuptake blockade (0.5-1.9 μg/mL), elevating cAMP through A2 receptor/adenylate cyclase activation  - Selective cGMP-PDE inhibition enhancing EDRF-mediated vasodilation  - Suppression of TXA2 synthesis  - Potentiation of endogenous PGI2  Additionally, it reduces heart rate in anesthetized cats/dogs and exerts cardioprotective effects against myocardial ischemia by limiting infarct size. |
| SX | NA |
| DC | This formulation exhibits antiplatelet aggregation, coronary artery dilation, blood viscosity reduction, erythrocyte velocity acceleration, microcirculation improvement, along with anti-myocardial ischemia and infarction properties. |
| DS | NA |
| DH | NA |
| SK | 1. Nephroprotective effects: Reduces serum urea nitrogen (BUN) and creatinine (Scr) levels while elevating hemoglobin (Hb) in chronic renal failure (CRF) model rats (5/6 nephrectomy or unilateral nephrectomy + adenine-induced).  2. Renal histopathological mitigation: Alleviates compensatory nephron lesions in CRF rats.  3. Immunomodulation: Enhances nonspecific phagocytic function of mononuclear macrophages and DNCB-induced delayed cutaneous hypersensitivity in immunosuppressed mice.  4. Hemorheological improvement: Decreases whole blood viscosity, plasma viscosity, and erythrocyte aggregation in blood stasis model rats. |
| KD | 1. Antioxidant and vascular effects: Inhibits oxygen free radicals, prevents ischemia-reperfusion injury, dilates blood vessels, increases cerebral/cardiac blood flow, enhances fibrinolysis, reduces myocardial oxygen consumption, improves microcirculation, and alleviates pain, sedation, and smooth muscle spasms.  2. Hemorheological modulation: Normalizes blood rheology parameters and regulates endothelin levels to exert blood-activating and stasis-resolving effects.  3. Antitumor activity: Demonstrates inhibitory effects on tumor progression.  4. Hepatic protection: Attenuates liver fibrosis in miniature swine induced by multifactorial insults.  5. Alcohol-induced liver injury mitigation: Protects against acute alcoholic liver damage.  6. Cardioprotection: Reduces injury in acute myocardial ischemia models. |
| ST | Animal studies demonstrate that this formulation:  1. Prolongs coagulation time in mice.  2. Reduces platelet aggregation/adhesion rates.  3. Inhibits venous thrombosis formation (in vivo/in vitro).  4. Increases femoral artery blood flow in embolized dogs.  5. Shortens plasma euglobulin lysis time.  6. Alleviates behavioral deficits in rats with middle cerebral artery occlusion. |
| GG | This formulation contains puerarin, a flavonoid glycoside derived from Pueraria lobata or P. thomsonii, with the following actions:  1. Vasodilation: Expands coronary/cerebral vasculature, reduces myocardial oxygen consumption, improves microcirculation, and inhibits platelet aggregation.  2. Animal studies confirm:  (1) Smooth muscle relaxation via total flavonoids, contrasting with contractile effects from choline/acetylcholine/casein R.  (2) Hypotensive effects in normotensive/hypertensive models  (3) Coronary artery dilation (40% blood flow increase, 29% resistance reduction at 30 mg/kg IV)  (4) Inhibition of thrombin-induced 5-HT release in platelets |

Note: The above information is from China Pharmaceutical Information Query Platform (recognised by the State Drug Administration of China)

Appendix Table A.3: More details about each TCMI

|  | Manufacturer | Batch number | Indications | Adverse reaction |
| --- | --- | --- | --- | --- |
| HQ | Jiangsu Jiuxu Pharmaceutical Co. | Z20003189 | Viral myocarditis and cardiac dysfunction presenting with Heart Qi deficiency and blood stasis obstruction in the cardiovascular system. Hepatitis manifesting as Spleen deficiency with dampness retention syndrome. | 1.Systemic reactions: Anaphylactoid reactions, anaphylactic shock, chills, pyrexia, facial pallor.  2.Respiratory system: Dyspnea, cyanosis, bronchospasm, cough.  3.Cardiovascular system: Palpitations, chest tightness.  4.Gastrointestinal system: Nausea, vomiting.  5.Dermatological reactions: Hyperhidrosis, skin rash, pruritus.  6.Neurological system: Dizziness, cephalalgia. |
|  | Zhengda Youthful Bao Pharmaceutical Co. | Z33020179; Z33020178 |  |  |
|  | China Shineway Pharmaceutical Group Co. | Z13020999 |  |  |
|  | Heilongjiang Jumbo Island Pharmaceutical Co. | Z23020862 |  |  |
|  | Shanghai Fuda Pharmaceutical Co. | Z31020083 |  |  |
|  | Chengdu Dior Pharmaceutical Co. | Z51021776 |  |  |
| YX | Guizhou Yibai Pharmaceutical Co. | H52020032 | Prophylaxis and therapeutic management of coronary artery disease.  Prevention and treatment of thromboembolic disorders. | 1.Gastrointestinal system disorders: Nausea, vomiting, abdominal discomfort, abdominal pain, abdominal distension, diarrhea, xerostomia.  2.Neurological system disorders: Dizziness, headache, cephalic distension, vertigo, local or generalized numbness and convulsions, tremors, visual disturbances, tinnitus.  3.Dermatological disorders: Skin rash, pruritus, hyperhidrosis, skin flushing or pallor with cyanosis.  4.Systemic disorders: Chills, rigors, pyrexia, generalized pain (including chest pain, lumbar pain), fatigue, trembling, edema, facial pallor.  5.Respiratory system disorders: Chest tightness, dyspnea, tachypnea, cough, laryngeal edema, suffocation.  6.Cardiovascular system disorders: Palpitations, tachycardia, arrhythmia, hypertension or hypotension.  7.Immune dysfunction and infections: Allergic reactions, anaphylactoid reactions, anaphylactic shock.  8.Administration site reactions: Phlebitis, injection site erythema and swelling, injection site pain.  9.Hematological disorders: Purpura, hemorrhage. |
|  | Tonghua Guhong Pharmaceutical Co. | H22026140 |  |  |
|  | Shanxi Puder Pharmaceutical Co. | H14023516 |  |  |
| SX | Heilongjiang Jumbo Island Pharmaceutical Co. | Z23022004 | This preparation is clinically indicated for ischemic cardiovascular and cerebrovascular diseases, including coronary artery disease, angina pectoris, cerebral embolism, and cerebrovascular spasm. | 1.Allergic reactions: Flushing, skin rash, pruritus, urticaria, allergic dermatitis, angioedema, laryngeal edema, dyspnea, bronchospasm, suffocation, palpitations, cyanosis, hypotension, anaphylactic shock.  2. Systemic disorders: Chills, hyperpyrexia, pyrexia, generalized pain, hyperhidrosis, allergic purpura, coma.  3. Respiratory system disorders: Tachypnea, cough.  4. Cardiovascular and cerebrovascular disorders: Palpitations, chest tightness, tachycardia, hypertension. When combined with other antiplatelet or anticoagulant agents, case reports of intracranial hemorrhage have been documented.  5. Gastrointestinal disorders: Xerostomia, anorexia, nausea, vomiting, gastrointestinal discomfort, abdominal distension, abdominal pain, diarrhea, constipation, abnormal hepatic biochemical parameters (e.g., elevated transaminases). Cases of gastrointestinal hemorrhage have been reported.  6. Dermatological disorders: Subcutaneous petechiae and ecchymosis.  7. Neuropsychiatric disorders: Dizziness, headache, convulsions, tremors, insomnia.  8. Others: Phlebitis, intraocular hemorrhage, hematuria. |
|  | Shijiazhuang Yinhu Pharmaceutical Co. | Z14021945 |  |  |
| DC | Guizhou Byte Pharmaceutical Co. | H52020959 | This preparation is clinically indicated for occlusive cerebrovascular diseases (e.g., cerebral hypoperfusion, thrombosis, embolism) and ischemic cardiovascular disorders including coronary artery disease-associated chest tightness, angina pectoris, myocardial infarction, ischemic stroke, and thromboangiitis obliterans. |  |
|  | Jilin Si Chang Pharmaceutical Co. | H22026448 |  | 1.Allergic reactions: Cutaneous flushing or pallor, skin rash, pruritus, chills, laryngeal edema, dyspnea, palpitations, cyanosis, hypotension progressing to shock.  2.Systemic disorders: Chills, cold intolerance, pyrexia (including hyperpyrexia), generalized pain, chest tightness, chest pain, fatigue, tremor, edema, facial pallor.  3.Dermatological disorders: Skin rash, pruritus, hyperhidrosis.  4.Cardiovascular disorders: Phlebitis, flushing, cyanosis, palpitations, arrhythmia, hypertension or hypotension.  5.Neurological disorders: Dizziness, headache, cephalic distension, local or generalized numbness, convulsions, agitation, tremors, vertigo, insomnia, mental disorders, somnolence.  6.Gastrointestinal disorders: Nausea, vomiting, abdominal pain, diarrhea, abdominal distension, xerostomia.  7.Respiratory disorders: Dyspnea, tachypnea, chest tightness, suffocation, cough, pharyngeal discomfort.  8.Musculoskeletal disorders: Myalgia, back pain, arthralgia.  9.Hematological disorders: Purpura, hemorrhage.  10.Administration site reactions: Injection site pain, injection site induration.  11.Others: Visual disturbances, tinnitus, hypoacusis. |
| DS | Zhengda Youthful Bao Pharmaceutical Co. | Z33020176 | This product is clinically indicated for coronary artery disease presenting with chest tightness and angina pectoris. | 1.Allergic reactions: Cutaneous flushing or pallor, skin rash, pruritus, chills, laryngeal edema, dyspnea, palpitations, cyanosis, hypotension progressing to shock.  2.Dermatological disorders: Skin rash (including erythema, papules, wheals), pruritus, hyperhidrosis, localized cutaneous reactions.  3.Systemic reactions: Cold intolerance, rigors, pyrexia (including hyperpyrexia), fatigue, generalized pain, facial pallor, edema, anaphylactic shock.  4.Respiratory disorders: Cough, pharyngeal discomfort, chest tightness, suffocation, dyspnea.  5.Cardiovascular disorders: Palpitations, chest tightness, suffocation, cyanosis, arrhythmia, hypertension or hypotension.  6.Gastrointestinal disorders: Nausea, vomiting, abdominal pain, abdominal distension, xerostomia.  7.Neuropsychiatric disorders: Dizziness, headache, convulsions, tremors, localized or generalized numbness.  8.Administration site reactions: Flushing, pain, purpura.  9.Others: Visual disturbances, facial discomfort. |
| DH | Shandong Pharmaceutical Co. | Z20026866 | This product is clinically indicated for chest obstruction syndrome and stroke due to blood stasis obstruction, presenting with chest pain, tightness, palpitations, facial deviation, dysarthria, limb numbness, and motor impairment; and for managing coronary artery disease, angina pectoris, myocardial infarction, blood stasis-pattern cor pulmonale, ischemic encephalopathy, and cerebral thrombosis. | 1.Allergic reactions: Facial flushing, skin rash, pruritus, urticaria, laryngeal edema, dyspnea, suffocation, palpitations, cyanosis, hypotension progressing to anaphylactic shock.  2.Systemic disorders: Chills, hyperpyrexia.  3.Cardiovascular disorders: Chest tightness, palpitations, hypertension.  4.Gastrointestinal disorders: Nausea, vomiting, abdominal pain, diarrhea. Cases of abnormal hepatic biochemical parameters (e.g., elevated transaminases) have been reported.  5.Neurological disorders: Dizziness, headache, convulsions, coma.  7. Hemorrhagic manifestations: Purpura, hematuria, epistaxis, gingival hemorrhage, conjunctival hemorrhage, gastrointestinal hemorrhage, subcutaneous petechiae and ecchymosis. |
| SK | Xi'an Century Shengkang Pharmaceutical Co. | Z20040110 | This product is clinically indicated for chronic kidney disease presenting with dampness-turbidity and blood stasis syndrome, characterized by nausea/vomiting, viscous oral sensation, sallow complexion, lethargy, lumbago, anorexia, abdominal distension, squamous skin, limb numbness, purplish tongue with ecchymosis, thick greasy tongue coating, and choppy or thready pulse. | Administration site reactions: Localized adverse reactions including erythema, pain, pruritus cutaneous rash, and xerostomia may occur. |
| KD | Shenyang Shuangding Pharmaceutical Co. | Z20025449 | This product is clinically indicated for chest obstruction syndrome due to blood stasis obstruction, characterized by chest tightness, precordial pain, bitter taste, dark-red tongue with ecchymosis; applicable to coronary artery disease and angina pectoris presenting these symptoms, and for cerebral infarction. | 1.Allergic reactions: Cutaneous flushing, skin rash, pruritus, dyspnea, suffocation, palpitations, cyanosis, hypotension progressing to anaphylactic shock.  (1) Rare: Severe manifestations including respiratory distress, convulsions, anaphylactic shock.  (2) Very rare: Delayed hypersensitivity reactions (Type IV) upon re-exposure or prolonged use.  3.Systemic disorders: Chills, pyrexia, malaise, hyperhidrosis, generalized pain, edema.  4.Respiratory disorders: Tachypnea, suffocation, cough.  5.Cardiovascular disorders: Palpitations, chest tightness, arrhythmia, precordial discomfort.  6.Gastrointestinal disorders: Nausea, vomiting, abdominal pain, diarrhea.  7.Dermatological disorders:  (1) Inflammatory lesions: Erysipelas.  (2) Eruptive lesions: Papules, erythematous rash。  (3) Local reactions: Cutaneous reactivity。  8.Neuropsychiatric disorders: Dizziness, headache, cephalic distension, vertigo, convulsions, agitation.  9.Administration site reactions: Phlebitis, Rash, pain, paresthesia  10. Others: Tremors. |
|  | Tonghua Huaxia Pharmaceutical Co. | Z20025450 |  |  |
| ST | Mudanjiang Youbo Pharmaceutical Co. | Z20010100 | This product is clinically indicated for acute-phase meridian-collateral stroke due to blood stasis obstruction, characterized by hemiplegia, facial deviation, and dysarthria; and for acute cerebral infarction presenting these clinical patterns. | 1.Hypersensitivity reactions: Cutaneous flushing, skin rash, pruritus, urticaria, laryngeal edema, dyspnea, suffocation, palpitations, cyanosis, hypotension, anaphylactic shock.  2.Systemic disorders: Rigors, pyrexia (including hyperpyrexia), cold intolerance, fatigue.  3.Respiratory disorders: Chest tightness, dyspnea, tachypnea, cough, suffocation.  4.Cardiovascular disorders: Palpitations.  5.Gastrointestinal disorders: Nausea, vomiting, abdominal pain, diarrhea.  6.Neurological disorders: Dizziness, headache, convulsions.  7.Dermatological disorders: Rash (maculopapular/erythematous), urticaria, erythema, pruritus, hyperhidrosis.  8.Others:  (1) Hemorrhagic events: Purpura, hematuria, gastrointestinal hemorrhage, conjunctival hemorrhage, subcutaneous hemorrhage.  (2) Coagulopathy: Prothrombin time abnormality. |
| GG | Zhejiang Kang Enbei Pharmaceutical Co. | H20056320 | This product is clinically indicated as adjunctive therapy for coronary artery disease, angina pectoris, myocardial infarction, retinal artery/vein occlusion, and sudden sensorineural hearing loss. | 1.Gastrointestinal reactions:  (1) Transient events: Initial administration may elicit abdominal distension and nausea.  (2) Resolution: Symptoms typically resolve spontaneously with continued administration.  2. Hypersensitivity reactions:  (1) Reported manifestations: Cutaneous rash, allergic asthma, anaphylactic shock.  (2) Clinical protocol: Immediate discontinuation and symptomatic management are mandated.  3. Hematological toxicity (Rare events): Acute intravascular hemolysis presenting with rigors, pyrexia, jaundice, lumbar pain, and hemoglobinuria. |

Note：The above information is from China Pharmaceutical Information Query Platform (recognised by the State Drug Administration of China)

Appendix Table A.4-A.13: Search Strategy

Table A.4: Search Strategy: PubMed

| #1 | "Diabetic Nephropathies"[Mesh] |
| --- | --- |
| #2 | ((((((((((((((((((Diabetic Nephropathies[Title/Abstract])) OR (Nephropathies, Diabetic[Title/Abstract])) OR (Nephropathy, Diabetic[Title/Abstract])) OR (Diabetic Nephropathy[Title/Abstract])) OR  (Diabetic Kidney Disease[Title/Abstract])) OR (Diabetic Kidney Diseases[Title/Abstract])) OR (Kidney Disease,  Diabetic[Title/Abstract])) OR (Kidney Diseases, Diabetic[Title/Abstract])) OR  (Diabetic Glomerulosclerosis[Title/Abstract])) OR (Glomerulosclerosis, Diabetic[Title/Abstract])) OR  (Intracapillary Glomerulosclerosis[Title/Abstract])) OR (Nodular Glomerulosclerosis[Title/Abstract])) OR  (Glomerulosclerosis, Nodular[Title/Abstract])) OR (Kimmelstiel-Wilson Syndrome[Title/Abstract])) OR  (Kimmelstiel Wilson Syndrome[Title/Abstract])) OR (Syndrome, Kimmelstiel-Wilson[Title/Abstract])) OR  (Kimmelstiel-Wilson Disease[Title/Abstract])) OR (Kimmelstiel Wilson Disease[Title/Abstract]) |
| #3 | #1 OR #2 |
| #4 | "Injections"[Mesh] |
| #5 | ((((Injections[Title/Abstract]) ) OR (Injection[Title/Abstract])) OR (Injectables[Title/Abstract]))  OR (Injectable[Title/Abstract]) |
| #6 | #4 OR #5 |
| #7 | "Angiotensin-Converting Enzyme Inhibitors"[Mesh] |
| #8 | ((((((((((((((((((((((((((((Angiotensin-Converting Enzyme Inhibitors) OR  (Angiotensin Converting Enzyme Inhibitors)) OR (Enzyme Inhibitors, Angiotensin-Converting)) OR  (Inhibitors, Angiotensin-Converting Enzyme)) OR (Inhibitors, Angiotensin Converting Enzyme)) OR  (Inhibitors, Kininase II)) OR (Kininase II Antagonists)) OR (Kininase II Inhibitors)) OR  (Angiotensin-Converting Enzyme Antagonists)) OR (Angiotensin Converting Enzyme Antagonists)) OR  (Enzyme Antagonists, Angiotensin-Converting)) OR (Kininase II Inhibitor)) OR  (II Inhibitor, Kininase)) OR (Inhibitor, Kininase II)) OR (Antagonists, Kininase II)) OR  (Inhibitors, ACE)) OR (ACE Inhibitors)) OR (Angiotensin I-Converting Enzyme Inhibitors)) OR  (Angiotensin I Converting Enzyme Inhibitors)) OR (Angiotensin Converting Enzyme Inhibitor)) OR  (ACE Inhibitor)) OR (Inhibitor, ACE)) OR (Angiotensin I-Converting Enzyme Inhibitor)) OR  (Angiotensin I Converting Enzyme Inhibitor)) OR (Angiotensin-Converting Enzyme Inhibitor)) OR  (Enzyme Inhibitor, Angiotensin-Converting)) OR (Inhibitor, Angiotensin-Converting Enzyme)) OR  (Antagonists, Angiotensin-Converting Enzyme)) OR (Antagonists, Angiotensin Converting Enzyme) |
| #9 | "Angiotensin Receptor Antagonists"[Mesh] |
| #10 | (((((((((((((Angiotensin Receptor Antagonists) OR (Antagonists, Angiotensin Receptor)) OR  (Receptor Antagonists, Angiotensin)) OR (Angiotensin Receptor Blockers)) OR (Receptor Blockers, Angiotensin)) OR (Angiotensin Receptor Blocker)) OR (Blocker, Angiotensin Receptor)) OR (Receptor Blocker, Angiotensin)) OR (Angiotensin Receptor Antagonist)) OR (Antagonist, Angiotensin Receptor)) OR  (Receptor Antagonist, Angiotensin)) OR (Angiotensin II Receptor Antagonists)) OR (Angiotensin II Receptor Blockers)) OR (Angiotensin II Receptor Antagonist) |
| #11 | #7 OR #8 OR #8 OR #10 |
| #12 | ((randomized controlled trial[Publication Type]) OR (randomized[Title/Abstract])) |
| #13 | #3 AND #6 AND #11 AND #12 |

Table A.5: Search Strategy: Web of Science

| #1 | ((((((((((((((((((TS=(Diabetic Nephropathies)) OR TS=(Nephropathies, Diabetic)) OR  TS=(Nephropathy, Diabetic)) OR TS=(Diabetic Nephropathy)) OR TS=(Diabetic Kidney Disease)) OR  TS=(Diabetic Kidney Diseases)) OR TS=(Kidney Disease, Diabetic)) OR TS=(Kidney Diseases, Diabetic)) OR  TS=(Diabetic Glomerulosclerosis)) OR TS=(Glomerulosclerosis, Diabetic)) OR  TS=(Intracapillary Glomerulosclerosis)) OR TS=(Nodular Glomerulosclerosis)) OR  TS=(Glomerulosclerosis, Nodular)) OR TS=(Kimmelstiel-Wilson Syndrome)) OR  TS=(Kimmelstiel Wilson Syndrome)) OR TS=(Syndrome, Kimmelstiel-Wilson)) OR  TS=(Syndrome, Kimmelstiel-Wilson)) OR TS=(Kimmelstiel-Wilson Disease)) OR  TS=(Kimmelstiel Wilson Disease) |
| --- | --- |
| #2 | (((TS=(Injections)) OR TS=(Injection)) OR TS=( Injectables)) OR TS=(Injectable) |
| #3 | (((((((((((ALL=(Angiotensin-Converting Enzyme Inhibitors)) OR  ALL=(Angiotensin Converting Enzyme Inhibitors)) OR ALL=(Enzyme Inhibitors, Angiotensin-Converting)) OR  ALL=(Inhibitors, Angiotensin-Converting Enzyme)) OR ALL=(Inhibitors, Angiotensin Converting Enzyme)) OR ALL=(Inhibitors, Kininase II)) OR ALL=(Angiotensin Receptor Antagonists)) OR  ALL=(Antagonists, Angiotensin Receptor)) OR ALL=(Receptor Antagonists, Angiotensin))  OR ALL=(Angiotensin Receptor Blockers)) OR ALL=(Receptor Blockers, Angiotensin)) OR  ALL=(Angiotensin Receptor Blocker) |
| #4 | (ALL=(randomized controlled trial)) OR ALL=(randomized) |
| #5 | #1 AND #2 AND #3 AND #4 |

Table A.6: Search Strategy: Scopus

| #1 | ( ( TITLE ( "Diabetic Nephropathies" ) OR TITLE ( "Nephropathies, Diabetic" ) OR TITLE ( "Nephropathy, Diabetic" ) OR TITLE ( "Diabetic Nephropathy" ) OR TITLE ( "Diabetic Kidney Disease" ) OR TITLE ( "Diabetic Kidney Diseases" ) OR TITLE ( "Kidney Disease Diabetic" ) OR TITLE ( "Kidney Diseases, Diabetic" ) OR TITLE ( "Diabetic Glomerulosclerosis" ) OR TITLE ( "Glomerulosclerosis, Diabetic" ) OR TITLE ( "Intracapillary Glomerulosclerosis" ) OR TITLE ( "Nodular Glomerulosclerosis" ) OR TITLE ( "Glomerulosclerosis, Nodular" ) OR TITLE ( "Kimmelstiel-Wilson Syndrome" ) OR TITLE ( "Kimmelstiel Wilson Syndrome" ) OR TITLE ( "Syndrome, Kimmelstiel-Wilson" ) OR TITLE ( "Syndrome, Kimmelstiel-Wilson" ) OR TITLE ( "Kimmelstiel-Wilson Disease" ) OR TITLE ( "Kimmelstiel Wilson Disease" ) ) ) AND ( ( TITLE ( "Injections" ) OR TITLE ( "Injection" ) OR TITLE ( "Injectables" ) OR TITLE ( "Injectable" ) ) ) AND ( ( ALL ( "Angiotensin-Converting Enzyme Inhibitors" ) OR ALL ( "Angiotensin Converting Enzyme Inhibitors" ) OR ALL ( "Enzyme Inhibitors, Angiotensin-Converting" ) OR ALL ( "Inhibitors, Angiotensin-Converting Enzyme" ) OR ALL ( "Inhibitors, Angiotensin Converting Enzyme" ) OR ALL ( "Inhibitors, Kininase II" ) OR ALL ( "Angiotensin Receptor Antagonists" ) OR ALL ( "Antagonists, Angiotensin Receptor" ) OR ALL ( "Receptor Antagonists, Angiotensin" ) OR ALL ( "Angiotensin Receptor Blockers" ) OR ALL ( "Receptor Blockers, Angiotensin" ) OR ALL ( "Angiotensin Receptor Blocker" ) ) ) AND ( ( ALL ( "randomized controlled trial" ) OR ALL ( "randomized" ) ) ) |
| --- | --- |

Table A.7: Search Strategy: Embase

| #1 | 'diabetic nephropathy'/exp |
| --- | --- |
| #2 | 'diabetic nephropathy':ti OR 'diabetic renal disease':ti OR 'diabetic nephropathies':ti OR 'diabetic kidney disease':ti OR 'diabetes nephropathy':ti |
| #3 | #1 OR #2 |
| #4 | 'injection'/exp |
| #5 | 'blood vessel injection':ti OR 'gluteal injection':ti OR 'injection solution':ti OR 'injections':ti OR 'percutaneous injection':ti OR 'injection':ti |
| #6 | #4 OR #5 |
| #7 | 'ace related carboxypeptidase' OR 'ace-related carboxypeptidase' OR 'ace2 angiotensin converting enzyme protein 2' OR 'ace2 angiotensin-converting enzyme protein 2' OR 'ace2 enzyme' OR 'ace2 protein' OR  'ace2 receptor' OR 'angiotensin converting enzyme 2 receptor' OR  'angiotensin converting enzyme ⅱ' OR 'angiotensin-converting enzyme 2' OR  'angiotensin-converting enzyme-related carboxypeptidase' OR 'dipeptidyl carboxypeptidase 2' OR  'enzyme ace2' OR 'protein ace2' OR 'receptor ace2' OR 'angiotensin converting enzyme 2' |
| #8 | 'angiotensin converting enzyme 2'/exp |
| #9 | 'angiotensin receptor antagonist'/exp |
| #10 | 'angiotensin ⅱ receptor antagonist' OR 'angiotensin ⅱ receptor antagonists' OR  'angiotensin ⅱ receptor blocker' OR 'angiotensin ⅱ receptor blockers' OR 'angiotensin ⅱ receptor blocking agent' OR 'angiotensin ⅱ receptor blocking agents' OR 'angiotensin receptor antagonists' OR 'angiotensin receptor blocker' OR 'angiotensin receptor blockers' OR 'angiotensin receptor blocking agent' OR 'angiotensin receptor blocking agents' OR 'angiotensin receptor antagonist' |
| #11 | #7 OR #8 OR #9 OR #10 |
| #12 | 'randomized controlled trial'/exp |
| #13 | 'controlled trial, randomized' OR 'randomised controlled study' OR 'randomised controlled trial' OR 'randomized controlled study' OR 'trial, randomized controlled' OR 'randomized controlled trial' |
| #14 | #12 OR #13 |
| #15 | #3 AND #6 AND #11 AND #14 |

Table A.8: Search Strategy: Cochrane Library

| #1 | MeSH descriptor: [Diabetic Nephropathies] explode all trees |
| --- | --- |
| #2 | Diabetic Nephropathies OR Kimmelstiel Wilson Disease OR Glomerulosclerosis, Nodular OR Kimmelstiel-Wilson Disease OR Syndrome, Kimmelstiel-Wilson OR Intracapillary Glomerulosclerosis OR Kimmelstiel Wilson Syndrome OR Kimmelstiel-Wilson Syndrome OR Nodular Glomerulosclerosis OR Diabetic Kidney Disease OR Kidney Disease, Diabetic OR Nephropathy, Diabetic OR Nephropathies, Diabetic OR Kidney Diseases, Diabetic OR Diabetic Kidney Diseases OR Diabetic Nephropathy OR Diabetic Glomerulosclerosis OR Glomerulosclerosis, Diabetic |
| #3 | #1 OR #2 |
| #4 | MeSH descriptor: [Injections] explode all trees |
| #5 | Injections OR Injection OR Injectable OR Injectables |
| #6 | #4 OR #5 |
| #7 | MeSH descriptor: [Injections] explode all trees |
| #8 | Angiotensin-Converting Enzyme Inhibitors OR Angiotensin Converting Enzyme Antagonists OR Inhibitors, Angiotensin Converting Enzyme OR Enzyme Antagonists, Angiotensin-Converting OR Kininase II Inhibitors OR Angiotensin-Converting Enzyme Antagonists OR Angiotensin I-Converting Enzyme Inhibitor OR Antagonists, Kininase II OR Angiotensin-Converting Enzyme Inhibitor OR Inhibitor, Angiotensin-Converting Enzyme OR Angiotensin I Converting Enzyme Inhibitor OR Antagonists, Angiotensin Converting Enzyme OR Angiotensin Converting Enzyme Inhibitors OR Antagonists, Angiotensin-Converting Enzyme OR Kininase II Inhibitor OR Angiotensin I-Converting Enzyme Inhibitors OR Enzyme Inhibitor, Angiotensin-Converting OR Inhibitors, Angiotensin-Converting Enzyme OR ACE Inhibitor OR II Inhibitor, Kininase OR Inhibitor, ACE OR ACE Inhibitors OR Angiotensin Converting Enzyme Inhibitor OR Kininase II Antagonists OR Enzyme Inhibitors, Angiotensin-Converting OR Inhibitor, Kininase II OR Inhibitors, Kininase II OR Angiotensin I Converting Enzyme Inhibitors OR Inhibitors, ACE |
| #9 | MeSH descriptor: [Injections] explode all trees |
| #10 | Angiotensin Receptor Blocker OR Receptor Antagonist, Angiotensin OR Angiotensin Receptor Blockers OR Receptor Blockers, Angiotensin OR Blocker, Angiotensin Receptor OR Receptor Antagonists, Angiotensin OR Antagonist, Angiotensin Receptor OR Receptor Blocker, Angiotensin OR Angiotensin Receptor Antagonist OR Antagonists, Angiotensin Receptor OR Angiotensin II Receptor Blockers OR Angiotensin II Receptor Antagonists OR Angiotensin II Receptor Blocker OR Angiotensin II Receptor Antagonist |
| #11 | #7 OR #8 OR #9 OR #10 |
| #12 | MeSH descriptor: [Injections] explode all trees |
| #13 | Randomized Controlled Trial OR randomized |
| #14 | #12 OR #13 |
| #15 | #3 AND #6 AND #11 AND #14 |

Table A.9: Search Strategy: China National Knowledge Infrastructure (CNKI)

| #1 | （主题：糖尿病肾病 + 糖尿病肾脏病变 + 毛细管间性肾小球硬化症 + 糖尿病性肾小球硬化症 + Kimmelstiel Wilson病 + 结节性肾小球硬化症 + 糖尿病肾疾病 + 基-威综合征）AND（主题：注射剂 + 注射液）AND（全文：ACEI + ARB + 沙坦 + 普利 + 血管紧张素转化酶抑制剂 + 血管紧张素Ⅱ受体拮抗剂(精确)）AND（全文：随机 + RCT + rcts(精确)） |
| --- | --- |

Table A.10: Search Strategy: the Chinese Scientific Journal database (VIP)

| #1 | ((((((((((题名或关键词=糖尿病肾病 OR 题名或关键词=糖尿病肾脏病变) OR 题名或关键词=毛细管间性肾小球硬化症) OR 题名或关键词=糖尿病性肾小球硬化症) OR 题名或关键词=糖尿病性肾小球硬化症) OR 题名或关键词=结节性肾小球硬化症) OR 题名或关键词=糖尿病肾疾病) OR 题名或关键词=基威综合征) AND ((题名或关键词=注射液 OR 题名或关键词=注射剂) OR 题名或关键词=注射)) AND ((任意字段=随机 OR 任意字段=RCT) OR 任意字段=rcts)) AND (((((任意字段=ACEI OR 任意字段=ARB) OR 任意字段=沙坦) OR 任意字段=普利) OR 任意字段=血管紧张素转化酶抑制剂) OR 任意字段=血管紧张素Ⅱ受体拮抗剂)) |
| --- | --- |

Table A.11: Search Strategy: Wanfang database

| #1 | 主题:(糖尿病肾病 OR 糖尿病肾脏病变 OR 毛细管间性肾小球硬化症 OR 糖尿病性肾小球硬化症 OR Kimmelstiel Wilson OR 结节性肾小球硬化症 OR 糖尿病肾疾病 OR 基-威综合征) and 主题:(注射剂 OR 注射液) and 全部:("随机" OR "RCT" OR "rcts") and 全部:("ACEI" OR "ARB" OR "沙坦" OR "普利" OR "血管紧张素转化酶抑制剂" OR "血管紧张素Ⅱ受体拮抗剂") |
| --- | --- |

Table A.12: Search Strategy: SinoMed

| #1 | "糖尿病肾病"[不加权:扩展] |
| --- | --- |
| #2 | "糖尿病肾病"[中文标题:智能] OR "糖尿病肾脏病变"[中文标题:智能] OR "毛细管间性肾小球硬化症"[中文标题:智能] OR "糖尿病性肾小球硬化症"[中文标题:智能] OR "Kimmelstiel"[中文标题:智能] OR "Wilson病"[中文标题:智能] |
| #3 | (#2) OR (#1) |
| #4 | "注射剂"[中文标题:智能] OR "注射液"[中文标题:智能] |
| #5 | "ACEI"[全部字段:智能] OR "ARB"[全部字段:智能] OR "沙坦"[常用字段:智能] OR "普利"[常用字段:智能] OR "血管紧张素转化酶抑制剂"[常用字段:智能] OR "血管紧张素Ⅱ受体拮抗剂"[常用字段:智能] |
| #6 | "血管紧张素转换酶抑制药"[不加权:扩展] |
| #7 | "血管紧张素受体拮抗剂"[不加权:扩展] |
| #8 | (#7) OR (#6) OR (#5) |
| #9 | "随机"[全部字段:智能] OR "RCT"[全部字段:智能] OR "rcts"[全部字段:智能] |
| #10 | (#9) AND (#8) AND (#4) AND (#3) |

Table A.13: Chinese Clinical Trial Registry (ChiCTR)

| #1 | 注册题目: 糖尿病肾病  研究疾病名称: 糖尿病肾病  干预措施: 注射液  研究类型: 干预性研究/Interventional study  公开试验结果文件: 是/Yes  All other options are default. |
| --- | --- |

Appendix Table A.14: Specific intervention methods of the study

|  | Study | Experimental group | Control group |
| --- | --- | --- | --- |
| 1 | Zhang YX 2021 | On the basis of the control group, HQ 50ml was added. | Routine treatment: diabetic diet; high-quality, low-protein diet; moderate or low intensity exercise programme; control of blood glucose and blood lipids.  On this basis, benazepril 10mg was given. |
| 2 | Zhang SB 2020 | On the basis of the control group, HQ 20ml was added. | Routine treatment: nutritional intervention and dietary guidance; moderate exercise; control of blood glucose.  On this basis, ACEI/ARB drug was given. |
| 3 | Qiao MF 2018 | On the basis of the control group, HQ 20ml was added. | Routine treatment: control of blood glucose.  On this basis, benazepril hydrochloride 10mg was given. |
| 4 | Zhang X 2017 | On the basis of the control group, HQ 20ml was added. | Routine treatment: control of blood glucose levels; low-fat, low-protein and high-calorie diet, and an appropriate and regular exercise programme for patients according to their gender, age and physical fitness. Calcium antagonists were added to patients with hypertension.  On this basis, valsartan 80mg was given. |
| 5 | Li H 2017 | On the basis of the control group, HQ 50ml was added. | Routine treatment: control of blood glucose; control of blood pressure and blood lipid; low-protein diet; low-salt and low-fat diet was given to those with combined hypertension.  On this basis, irbesartan 150mg was given. If blood pressure was >130/80mmHg, diuretics and calcium channel blockers were added. |
| 6 | Tang H 2016 | On the basis of the control group, HQ 20ml was added. | Routine treatment: basic diabetes mellitus treatment, including health education, dietary control, high-protein diet；appropriate exercise；other symptomatic supportive treatment.  On this basis, valsartan 80mg was given. |
| 7 | Ren YJ 2016 | On the basis of the control group, HQ 50ml was added. | Routine treatment: control of blood glucose; low-fat, low-protein, low-salt and other high-quality diet; moderate exercise.  On this basis, irbesartan 150mg was given, and if blood pressure was high, calcium channel blockers, antihypertensives and diuretics were added. |
| 8 | Liu SY 2016 | On the basis of the control group, HQ 20ml was added. | Routine treatment: insulin control of blood glucose; diet control; appropriate exercise; health education.  On this basis, valsartan 80mg was given. |
| 9 | Cai R 2016 | On the basis of the control group, HQ 50ml was added. | Routine treatment: reasonable weight control; diabetic diet; appropriate exercise; low-protein diet; control of blood glucose and blood pressure; correction of dyslipidaemia.  On this basis, benazepril 10 mg was given. |
| 10 | Wang XH 2015 | On the basis of the control group, HQ 50ml was added. | Routine treatment: low-protein diet; diabetic nephropathy related knowledge education; combined with hypertension need low-salt, low-fat diet, appropriate exercise; hypoglycemic drugs or insulin to control blood glucose; control of blood pressure and blood lipids  On this basis, irbesartan 150mg was given. If blood pressure > 130/80 mmHg, calcium channel blocker antihypertensive drugs and diuretics were added. |
| 11 | Liu XX 2015 | On the basis of the control group, HQ 50ml was added. | Routine treatment: high-quality, low-protein diabetic diet; low or moderate intensity exercise; application of insulin and hypoglycaemic drugs to control blood glucose; control of blood pressure ≤130/80 mmHg. If the control did not meet the standard, calcium antagonist or β-blocker drugs were added.  On this basis, benazepril 10mg was given. |
| 12 | Liu S 2015 | On the basis of the control group, HQ 40ml was added. | Routine treatment: moderate exercise; teaching about health knowledge; low-protein and high-quality diet; temporary prohibition of the use of other vasodilators, anticoagulants, antiplatelet agents, traditional Chinese medicines, etc.; hypertensive patients should not take ACEI/ARB drugs as antihypertensive medications, and give preference to calcium-ionic anticlipotent agents, so as to keep the blood pressure at <130/80 mm Hg; glucose control with preference for gliquidone, repaglinide drug.  On this basis, irbesartan 150mg was given. |
| 13 | Li SB 2015 | On the basis of the control group, HQ 30ml was added. | Routine treatment: control of blood glucose; if necessary, the addition of calcium channel blockers or β-blockers in cases of hypertension until the blood pressure was below 125/75 mmHg.  On this basis, benazepril hydrochloride was given. |
| 14 | Zhang Q 2014 | On the basis of the control group, HQ 60ml was added. | Routine treatment: dietary guidance; sensible exercise; stabilisation of blood glucose ; control of blood pressure.  On this basis, losartan 50mg was given. |
| 15 | Sun YH 2014 | On the basis of the control group, HQ 20ml was added. | Routine treatment: diabetes education; diabetes diet; blood glucose control with hypoglycaemic drugs or insulin.  On this basis, telmisartan 40mg was given. |
| 16 | Liu XX 2013 | On the basis of the control group, HQ 50ml was added. | Routine treatment: diabetic diet, low-salt diet, dietary calorie control, and high- quality, low-protein diet; exercise therapy programme stipulated at low or moderate intensity; anticoagulation and lipid lowering; blood glucose control with insulin and hypoglycemic agents; control of blood pressure ≤130/80 mmHg, and the addition of calcium antagonists or β-blockers when blood pressure control was unsatisfactory.  On this basis, benazepril 10mg was given. |
| 17 | Huang SL 2013 | On the basis of the control group, HQ 20ml was added. | Routine treatment: low-salt, low-fat diabetic diet; glucose control with hypoglycaemic drugs that have less effect on renal function.  On this basis, losartan 50mg was given. |
| 18 | Zhang Y 2012 | On the basis of the control group, HQ 20ml was added. | Routine treatment: diabetes mellitus health education; dietary control; appropriate exercise; insulin for blood glucose control.  On this basis, losartan 80mg was given. |
| 19 | Chen XX 2012 | On the basis of the control group, HQ 40ml was added. | Routine treatment: low-salt, low-fat, high-quality, low-protein and diabetic diet; smoking cessation; moderate exercise; choice of hypoglycaemic drugs that cause less damage to the kidneys and insulin to control blood glucose.  On this basis, benazepril hydrochloride 10mg was given. |
| 20 | Zhu LY 2011 | On the basis of the control group, HQ 40ml was added. | Routine treatment: low-salt diabetic diet and insulin for glucose control.  On this basis, valsartan 80mg was given. |
| 21 | Zeng J 2010 | On the basis of the control group, HQ 40ml was added. | Routine treatment: low-salt, low-protein diabetic diet; avoidance of smoking and alcohol; moderate exercise; control of blood glucose and lipids.  On this basis, enalapril 10mg was given. |
| 22 | Li ZQ 2010 | On the basis of the control group, HQ 50ml was added. | Routine treatment: dietary control, exercise therapy, low-fat diet; hypoglycaemic agents, insulin to control blood glucose; low-protein diet; in those with combined hypertension, antihypertensive drugs other than ACEI/ARB.  On this basis, telmisartan 40 mg was given. |
| 23 | Huan HM 2010 | On the basis of the control group, HQ 30ml was added. | Routine treatment: low-salt, low-protein diabetic diet; smoking cessation; moderate exercise and regular treatment to lower blood sugar and lipids.  On this basis, losartan Potassium 100mg was given. |
| 24 | Geng F 2010 | On the basis of the control group, HQ 60ml was added. | Routine treatment: diabetes mellitus education; moderate exercise; high-quality, low-protein diet; glucose-lowering drugs and insulin to control blood glucose; patients with hypertension, if the effect of Benadryl alone is not satisfactory in lowering blood pressure, then added nifedipine.  On this basis, benazepril 10mg was given. |
| 25 | Xu ZX 2009 | On the basis of the control group, HQ 40ml was added. | Routine treatment: insulin for blood glucose control.  On this basis, valsartan 80 mg was given. |
| 26 | Tian PW 2009 | On the basis of the control group, HQ 50mlwas added. | Routine treatment: diabetes mellitus conventional treatment.  On this basis, enalapril 10 mg was given. |
| 27 | Cui BS 2009 | On the basis of the control group, HQ 50ml was added. | Routine treatment: diabetes education; high-quality, low-protein diet; glucose-lowering medications to control blood glucose.  On this basis, captopril 75mg was given. |
| 28 | Xu XL 2008 | On the basis of the control group, HQ 60ml was added. | Routine treatment: dietary control; exercise therapy; high-quality, low-protein diet; insulin to control blood sugar; control of blood pressure within 130/80 mmHg; long-acting calcium channel blockers may be added if necessary.  On this basis, losartan 50mg was given. |
| 29 | Chen JN 2010 | On the basis of the control group, HQ 20ml was added. | Routine treatment: diabetes education; diabetic diet; blood pressure control; conventional insulin therapy.  On this basis, irbesartan 150mg was given. |
| 30 | Jiang L 2005 | On the basis of the control group, HQ 30ml was added. | Routine treatment: high-quality, low-protein diet; health education; control of blood glucose; control of blood pressure and blood lipids.  On this basis, benazepril 10mg was given. |
| 31 | Li HJ 2007 | On the basis of the control group, HQ 40ml was added. | Routine treatment:blood glucose control with hypoglycaemic drugs or insulin on the basis of dietary modification.  On this basis, captopril 75mg was given. |
| 32 | Huang W 2004 | On the basis of the control group, HQ 40ml was added. | Routine treatment: diabetes education; high-quality, low-protein diet; blood glucose control.  On this basis, benazepril hydrochloride 10-30mg was given, and the dosage was adjusted according to blood pressure. |
| 33 | Ma CH 2006 | On the basis of the control group, HQ 40ml was added. | Routine treatment: diabetes education; high-quality, low-protein diet; blood glucose control with insulin or hypoglycaemic agents.  On this basis, enalapril 10 mg was given. |
| 34 | Shi XH 2010 | On the basis of the control group, YX 20ml was added. | Routine treatment: dietary control; medication or insulin control; blood pressure control; dietary control; medication or insulin control; blood pressure control (amlodipine) at about 130/80mmHg.  On this basis, benazepril 10mg was given. |
| 35 | Zhang CJ 2013 | On the basis of the control group, YX 20ml was added. | Routine treatment: high-quality, low-protein diet; injection of insulin or drugs to control blood sugar; strict control of lipids and blood pressure complications; appropriate exercise.  On this basis, irbesartan 75mg was given. |
| 36 | Wang GC 2012 | On the basis of the control group, YX 25ml was added. | Routine treatment: control of blood glucose; stabilisation of blood pressure; regulation of blood lipids, anti-infection; improvement of microcirculation; correction of water-electrolyte disorders.  On this basis, valsartan 20mg was given. |
| 37 | Chen JS 2015 | On the basis of the control group, YX 25ml was added. | Routine treatment: insulin to control blood glucose; low-salt, low-fat diet; diabetes health education; moderate exercise.  On this basis, valsartan 80mg was given. |
| 38 | Wang QZ 2011 | On the basis of the control group, YX 20ml was added. | Routine treatment: diabetes education; dietary control; insulin or medication to control blood glucose.  On this basis, irbesartan 150mg was given. |
| 39 | Xing LY 2022 | On the basis of the control group, YX 20ml was added. | Routine treatment: dietary control; oral hypoglycaemic agents.  On this basis, candesartan cilexetil 8mg was given. |
| 40 | Shen SL 2017 | On the basis of the control group, YX 20ml was added. | Routine treatment: oral hypoglycaemic drugs or insulin were taken to control blood glucose.  On this basis, benazepril hydrochloride 5mg was given. |
| 41 | Zhang YM 2015 | On the basis of the control group, YX 25ml was added. | Routine treatment: low-sugar, low-fat, low-protein diet with vegetables and crude fibre as the mainstay; strengthened exercise; reasonable control of blood pressure.  On this basis, irbesartan 150 mg was given. |
| 42 | Li H 2014 | On the basis of the control group, YX 25ml was added. | Routine treatment: control of protein intake; moderate exercise; insulin or hypoglycaemic drugs to control blood glucose.  On this basis, irbesartan 300 mg was given. |
| 43 | Liu YX 2019 | On the basis of the control group, YX 25ml was added. | Routine treatment: control of blood glucose, stabilisation of water-electrolyte balance.  On this basis, candesartan cilexetil 8mg was given. |
| 44 | Huang JY 2017 | On the basis of the control group, YX 20ml was added. | Routine treatment: the basic treatment of diabetes mellitus includes diet control, reasonable exercise, stabilisation of blood pressure, regulation of blood lipids and prevention of infection.  On this basis, candesartan cilexetil 8mg was given. |
| 45 | Cheng H 2018 | On the basis of the control group, YX 20ml was added. | Routine treatment: diet control and use of exercise therapy.  On this basis, telmisartan 80mg was given. |
| 46 | Zhu J 2020 | On the basis of the control group, YX 25ml was added. | Routine treatment: dietary control; lipid lowering; oral or subcutaneous hypoglycemic agents.  On this basis, valsartan 80mg was given. |
| 47 | You W 2014 | On the basis of the control group, YX 20ml was added. | Routine treatment: dietary and lipid control; oral or injectable hypoglycaemic drugs.  On this basis, valsartan 80mg was given. |
| 48 | Li L 2012 | On the basis of the control group, YX 20ml was added. | Routine treatment: health education; rational diet; control of blood lipids; hypoglycemic drugs or insulin to control blood glucose.  On this basis, valsartan 80mg was given. |
| 49 | Li SH 2009 | On the basis of the control group, YX 20ml was added. | Routine treatment: diabetic diet; strict control of blood glucose and blood pressure.  On this basis, fosinopril sodium 10mg was given. |
| 50 | Li ZY 2013 | On the basis of the control group, YX 20ml was added. | Routine treatment: control of blood glucose and blood pressure.  On this basis, valsartan 80mg was given. |
| 51 | Wu GY 2017 | On the basis of the control group, SX 2ml was added. | Benazepril hydrochloride 10mg was given. |
| 52 | Yang QH 2007 | On the basis of the control group, SX 20ml was added. | Routine treatment: blood glucose control; diet control.  On this basis, benazepril 80mg was given. |
| 53 | Xu YY 2020 | On the basis of the control group, SX 20mg was added. | Routine treatment: dietary management; control of blood glucose and blood lipids.  On this basis, losartan potassium 100mg was given. |
| 54 | Zhen D 2012 | On the basis of the control group, SX 30ml was added. | Routine treatment: low-salt, low-protein diabetic diet; conventional blood glucose control and lipid-lowering treatment.  On this basis, telmisartan 80mg was given. |
| 55 | Geng GM 2013 | On the basis of the control group, DC 10ml was added. | Routine treatment: diabetes education; control of protein intake; blood glucose control with hypoglycaemic drugs and insulin.  On this basis, irbesartan tablets 300mg was given. |
| 56 | Tang N 2019 | On the basis of the control group, DC 10ml was added. | Routine treatment: insulin injections to control blood glucose; blood pressure control for those with hypertension; maintenance of acid-base and water-electrolyte balance.  On this basis, fosinopril sodium 20mg was given. |
| 57 | Gong J 2022 | On the basis of the control group, DC 10ml was added. | Routine treatment: control of blood glucose and blood pressure.  On this basis, fosinopril sodium 20mg was given. |
| 58 | Xian LH 2015 | On the basis of the control group, DC 10ml was added. | Routine treatment: diabetic diet; control of blood glucose; control of blood pressure; control of blood lipids.  On this basis, valsartan 80mg was given. |
| 59 | Zhang Y 2014 | On the basis of the control group, DC 10ml was added. | Routine treatment: diabetes health education; medical nutrition therapy; physical activity.  On this basis, losartan potassium 50mg was given. |
| 60 | Tao L 2015 | On the basis of the control group, DC 10ml was added. | Routine treatment: low-protein diet; control of patients’ fasting blood glucose.  On this basis, benazepril 20mg was given. |
| 61 | Tao Y 2016 | On the basis of the control group, DC 10ml was added. | Routine treatment: control of blood glucose; sensible exercise.  On this basis, losartan 50mg was given. |
| 62 | Zhang ZM 2014 | On the basis of the control group, DC 10ml was added. | Routine treatment: blood glucose control.  On this basis, irbesartan 150mg was given. |
| 63 | Fan WK 2017 | On the basis of the control group, DC 20ml was added. | Routine treatment: diet control; blood glucose regulation; antihypertensive therapy; lipid regulation therapy; reasonable exercise; health education.  On this basis, valsartan 80mg was given. |
| 64 | Zhou AJ 2009 | On the basis of the control group, DS 20ml was added. | Routine treatment: diabetic diet; blood pressure control, plus ACEI drugs if blood pressure control was unsatisfactory.  On this basis,, irbesartan 150mg was given. |
| 65 | Wei HB 2016 | On the basis of the control group, DS 10ml was added. | Routine treatment: diabetes education; dietary control; blood pressure control; exercise therapy.  On this basis, valsartan 80mg was given. |
| 66 | Hu B 2016 | On the basis of the control group, DS 400mg was added. | Routine treatment: insulin and hypoglycaemic drugs for blood glucose control; nifedipine controlled-release tablets for blood pressure control.  On this basis, Benazepril 10mg was given. |
| 67 | Xian XW 2014 | On the basis of the control group, DS 10ml was added. | Enalapril maleate 10mg was given. |
| 68 | Tian YH 2015 | On the basis of the control group, DS 800mg was added. | Irbesartan 150mg was given. |
| 69 | Li QY 2011 | On the basis of the control group, DH 40ml was added. | Routine treatment: blood glucose control and antiplatelet therapy.  On this basis, irbesartan 150mg was given. If blood pressure was not up to the standard≥130/80mmHg, amlodipine benzenesulfonate to lower blood pressure was added. |
| 70 | Wang CQ 2012 | On the basis of the control group, DH 20ml was added. | Routine treatment: low-protein diet; control of lipids and blood glucose.  On this basis, benazepril 20mg was given. |
| 71 | Huang YJ 2016 | On the basis of the control group, DH 30ml was added. | Routine treatment: diabetic diet; glucose-lowering drugs and insulin for blood glucose control.  On this basis, valsartan 160mg was given. |
| 72 | Ye XA 2016 | On the basis of the control group, DH 20ml was added. | Routine treatment: control of blood glucose; control of blood pressure; control of diet and exercise.  On this basis, valsartan 80mg was given. |
| 73 | Li JJ 2009 | On the basis of the control group, DH 30ml was added. | Routine treatment: control of blood glucose.  On this basis, valsartan 8mg was given. |
| 74 | Liu W 2009 | On the basis of the control group, DH 40ml was added. | Routine treatment: control of blood glucose; control of diet and exercise; control of blood pressure and administration of calcium channel blockers if necessary.  On this basis, enalapril 10mg was given. |
| 75 | Jing XN 2014 | On the basis of the control group, DH 30-50ml was added. | Routine treatment: oral hypoglycaemic drugs or application of insulin therapy to control blood glucose.  On this basis, fosinopril 10mg was given. |
| 76 | Tao GL 2019 | On the basis of the control group, SK 100ml was added. | Routine treatment: diabetes education; moderate exercise; dietary control and glycaemic control.  On this basis, candesartan cilexetil were started at 4mg and increased to 8mg after one week of treatment. |
| 77 | He LF 2020 | On the basis of the control group, SK 100ml was added. | Routine treatment: diabetic diet; moderate exercise; application of hypoglycemic drugs to keep blood glucose stable; lipid regulation, antiplatelet aggregation and other symptomatic treatment.  On this basis, valsartan 80mg was given. |
| 78 | Hao JL 2022 | On the basis of the control group, SK 100ml was added. | Routine treatment: blood glucose control; lipid control; high-quality, low-protein diet; blood pressure control; moderate exercise.  On this basis, valsartan 80mg was given. |
| 79 | Wang AY2013 | On the basis of the control group, SK 100ml was added. | Routine treatment: diabetes knowledge education; moderate exercise; diet control; strict blood glucose control; strict blood pressure control.  On this basis, enalapril 5mg was given. |
| 80 | Ren LL 2018 | On the basis of the control group, SK 100ml was added. | Routine treatment: low-protein diet; blood glucose control, correction of anaemia.  On this basis, irbesartan 150mg was given. |
| 81 | Li HF 2022 | On the basis of the control group, SK 100ml was added. | Routine treatment: correction of anaemia; low-protein diet; blood glucose control.  On this basis, irbesartan 150mg was given. |
| 82 | Xiao L 2013 | On the basis of the control group, SK 100ml was added. | Routine treatment: health education; dietary guidance; exercise; control of blood glucose.  On this basis, losartan 50mg was given. |
| 83 | Deng ZH 2021 | On the basis of the control group, SK 60ml was added. | Routine treatment: oral hypoglycemic drugs or insulin to control blood glucose; low-sugar, low-salt and low-protein diet; strengthened dietary health management; in patients with symptoms of hypertension, diuretics, calcium channel blockers and other drugs to control blood pressure levels.  On this basis, valsartan 160mg was given. |
| 84 | Hu YL 2015 | On the basis of the control group, SK 100ml was added. | Routine treatment: diabetes health education; moderate exercise; control of blood glucose and lipid-lowering therapy; high-quality, low-protein diet.  On this basis, telmisartan was given, and the dosage was adjusted according to individual blood pressure. |
| 85 | Ma YQ 2019 | On the basis of the control group, SK 100ml was added. | Routine treatment: moderate exercise; high-quality, low-protein diet; lipid, glucose, and blood pressure control.  On this basis, enalapril 5mg was given. |
| 86 | Chen P 2021 | On the basis of the control group, SK 100ml was added. | Valsartan 80mg was given. |
| 87 | Song LH 2012 | On the basis of the control group, SK 80ml was added. | Routine treatment: high-quality, low-protein diet.  On this basis, losartan 100mg was given. If the blood pressure cannot be controlled below 130/80 mmHg, amlodipine tablets was added to control blood pressure. |
| 88 | Rao GF 2013 | On the basis of the control group, KD 40ml was added. | Routine treatment: high-quality, low-protein diabetic diet; appropriate exercise; insulin control of blood glucose; regulation of blood lipids.  On this basis, irbesartan 150mg was given. |
| 89 | Jin QJ 2016 | On the basis of the control group, KD 40ml was added. | Routine treatment: health education; rational exercise; regulation of dietary habits; attention to the control of blood lipids and blood glucose.  On this basis, candesartan 8 mg was given. |
| 90 | Zheng JJ 2013 | On the basis of the control group, KD 40ml was added. | Routine treatment: strict control of hypertension, hyperglycaemia and body mass; correction of lipid metabolism disorders; antiplatelet therapy.  On this basis, benazepril 5~10 mg was given. |
| 91 | Zhang SJ 2023 | On the basis of the control group, KD 30ml was added. | Routine treatment: control of blood glucose; health education; appropriate sports; strict diet control; quit smoking and drinking; regular work and rest；avoid overwork;  On this basis, enalapriltablets 10mg was given. |
| 92 | Li HF 2010 | On the basis of the control group, ST 6ml was added. | administration of different doses of hypoglycaemic and antihypertensive drugs according to blood glucose and blood pressure levels; maintenance of electrolyte balance.  On this basis, Benazepril hydrochloride 10 mg was given. |
| 93 | Sun XP 2015 | On the basis of the control group, ST 6ml was added. | Routine treatment: diabetes mellitus health education; high-quality, low-protein diet; moderate exercise; insulin to control blood glucose and symptomatic treatment; those with hypertension were given ARB/ACEI to control blood pressure.  On this basis, olmesartan 40mg was given. |
| 94 | Li B 2013 | On the basis of the control group, ST 4ml was added. | Routine treatment: dietary control and exercise therapy to blood glucose.  On this basis, 5-10 mg of benazepril was administered at a dose determined by blood pressure. |
| 95 | Qin WG 2010 | On the basis of the control group, ST 8ml was added. | Routine treatment: health education; high-quality, low-protein diet; moderate exercise and hypoglycaemic therapy.  On this basis, benazepril hydrochloride 10-20mg was given, and the drug was individualised according to blood pressure. |
| 96 | Zhu YX 2003 | On the basis of the control group, GG 10ml was added. | Routine treatment: conventional treatment of diabetes mellitus, including dietary control and insulin therapy to keep blood glucose at a good level; control of blood pressure.  On this basis, enalapril 10mg was given. |
| 97 | Xu JQ 2011 | On the basis of the control group, GG 400mg was added. | Routine treatment: conventional treatment for diabetes mellitus, including dietary therapy and moderate exercise; oral hypoglycaemic drugs or insulin subcutaneous injections were used to control blood glucose according to different conditions.  On this basis, benazepril hydrochloride 10mg was used. |
| 98 | Yuan DY 2009 | On the basis of the control group, GG 500mg was added. | Benazepril hydrochloride 10mg was given. |
| 99 | Jiao FZ 2011 | On the basis of the control group, GG 10ml (including 500mg) was added. | Routine treatment: diabetic diet; reasonable exercise; basic treatment; control protein intake; comprehensive drug therapy to control, so that blood pressure, blood glucose to meet the standard.  On this basis, captopril 50-75mg was given. |

Appendix Table A.15: Basic information on the included studies

| Number | Study | Group (n) | Follow-up duration | Intervation | Age (mean ± SD), years | Sex (Male /Female） | Diabetes duration  (mean ± SD), years | Disease Staging, years | Outcomes |
| --- | --- | --- | --- | --- | --- | --- | --- | --- | --- |
|  |  |  |  |  |  |  |  |  |  |
| 1 | Zhang YX 2021 | Combination:50 | NA | HQ+ACEI | 57.9±7.8 | 27/23 | 7.6±1.4 | NA | ②⑤ |
|  |  | Control:50 |  | ACEI | 57.3±7.6 | 26/24 | 7.8±1.6 |  |  |
| 2 | Zhang SB 2020 | Combination:40 | 4 weeks | HQ+ACEI/ARB | 48.22±11.26 | 24/16 | 6.97±5.19 | Ⅲ,Ⅳ | ①③④ |
|  |  | Control:40 |  | ACEI/ARB | 46.58±12.05 | 21/19 | 6.48±4.59 |  |  |
| 3 | Qiao MF 2018 | Combination:40 | 4 weeks | HQ+ACEI | 58.5±12.5 | 23/17 | 7.5±3.5 | NA | ①③④⑥⑦ |
|  |  | Control:40 |  | ACEI | 58.5±11.5 | 24/16 | 6.5±3.5 |  |  |
| 4 | Zhang X 2017 | Combination:89 | 3 weeks | HQ+ARB | 54.8±1.5 | 64/25 | 4.9±0.5 | Ⅲ | ①②③④⑤ |
|  |  | Control:89 |  | ARB | 55.1±1.2 | 59/30 | 5.1±0.3 |  |  |
| 5 | Li H 2017 | Combination:55 | 4 weeks | HQ+ARB | NA | 32/23 | 7.8±2.7 | Ⅲ | ③④ |
|  |  | Control:55 |  | ARB | NA | 33/22 | 7.9±2.7 |  |  |
| 6 | Tang H 2016 | Combination:72 | 4 weeks | HQ+ARB | 52.48±4.11 | 42/30 | 2.11±0.78 | Ⅲ | ③④⑤ |
|  |  | Control:72 |  | ARB | 52.13±4.23 | 45/27 | 2.09±0.81 |  |  |
| 7 | Ren YJ 2016 | Combination:45 | 4 weeks | HQ+ARB | 57.6±5.3 | NA | 6.7±2.2 | Ⅲ | ②④⑤⑥⑦ |
|  |  | Control:45 |  | ARB | 56.9±5.8 | NA | 6.6±2.4 |  |  |
| 8 | Liu SY 2016 | Combination:37 | 3 weeks | HQ+ARB | 60.2±8.4 | 18/19 | 10.6±7.5 | NA | ①④ |
|  |  | Control:37 |  | ARB | 56.7±7.5 | 20/17 | 6.6±2.5 |  |  |
| 9 | Cai R 2016 | Combination:40 | 4 weeks | HQ+ACEI | 56.5±10.2 | 22/18 | 7.8±2.1 | Ⅲ | ①②⑤ |
|  |  | Control:40 |  | ACEI | 57.2±9.7 | 24/16 | 7.9±1.8 |  |  |
| 10 | Wang XH 2015 | Combination:54 | 4 weeks | HQ+ARB | 68.5±12.5 | 30/24 | 7.4±2.6 | NA | ③④ |
|  |  | Control:54 |  | ARB | 68.1±11.9 | 31/23 | 7.2±2.8 |  |  |
| 11 | Liu XX 2015 | Combination:36 | 30 days | HQ+ACEI | 54.0±10.8 | 21/15 | 6.4±3.5 | Ⅲ | ②③④⑥⑦ |
|  |  | Control:36 |  | ACEI | 53.8±10.7 | 20/16 | 6.3±3.7 |  |  |
| 12 | Liu S 2015 | Combination:28 | 4 weeks | HQ+ARB | 67.0±7.1 | 14/14 | 7.3±2.0 | Ⅲ | ①②④ |
|  |  | Control:28 |  | ARB | 67.5±7.2 | 15/13 | 7.2±1.7 |  |  |
| 13 | Li SB 2015 | Combination:30 | 4 weeks | HQ+ACEI | 41.2±3.2 | 19/11 | NA | Ⅲ,Ⅳ | ② |
|  |  | Control:30 |  | ACEI | 40.8±3.3 | 17/13 | NA |  |  |
| 14 | Zhang Q 2014 | Combination:28 | 4 weeks | HQ+ARB | 51.25±10.42 | 16/12 | 9.42±3.21 | Ⅲ | ②③④ |
|  |  | Control:28 |  | ARB | 51.41±10.54 | 17/11 | 9.41±3.20 |  |  |
| 15 | Sun YH 2014 | Combination:38 | 2 weeks | HQ+ARB | 52.63±9.12 | 22/16 | 6.1±1.4 | Ⅲ | ②③④ |
|  |  | Control:38 |  | ARB | 51.84±8.63 | 18/20 | 5.9±1.7 |  |  |
| 16 | Liu XX 2013 | Combination:39 | 30 days | HQ+ACEI | 53.6±11.4 | 23/16 | 7.3±3.5 | Ⅲ | ②⑤ |
|  |  | Control:38 |  | ACEI | 53.4±10.9 | 27/11 | 7.4±3.6 |  |  |
| 17 | Huang SL 2013 | Combination:50 | 4 weeks | HQ+ARB | 67.6±3.4 | NA | 12.5±2.4 | NA | ③④⑦ |
|  |  | Control:50 |  | ARB | 66.5±3.6 | NA | 11.4±3.2 |  |  |
| 18 | Zhang Y 2012 | Combination:30 | 3 weeks | HQ+ARB | 52.6±5.3 | 23/16 | 10.4±7.8 | Ⅲ | ①④ |
|  |  | Control:30 |  | ARB | 50.7±7.3 | 27/11 | 11.3±7.1 |  |  |
| 19 | Chen XX 2012 | Combination:40 | 6 weeks | HQ+ACEI | 47.3±7.3 | 17/21 | 6.1±5.8 | Ⅲ | ②③④⑤⑧ |
|  |  | Control:38 |  | ACEI | 48.1±5.6 | 18/22 | 6.0±6.4 |  |  |
| 20 | Zhu LY 2011 | Combination:28 | 4 weeks | HQ+ARB | 49.8±13.1 | 13/15 | 12.3±6.8 | Ⅲ | ②③④ |
|  |  | Control:28 |  | ARB | 48.6±13.7 | 14/14 | 14.6±7.8 |  |  |
| 21 | Zeng J 2010 | Combination:26 | 4 weeks | HQ+ACEI | 49.3±10.5 | 16/10 | 10.8±5.5 | Ⅲ | ②③④⑤⑧ |
|  |  | Control:26 |  | ACEI | 51.8±9.5 | 26/15 | 11.2±5.8 |  |  |
| 22 | Li ZQ 2010 | Combination:42 | 4 weeks | HQ+ARB | 58.4±8.2 | 26/16 | 6.8±4.2 | Ⅲ | ②④ |
|  |  | Control:28 |  | ARB | 56.2±7.4 | 18/10 | NA |  |  |

| Number | Study | Group (n) | Follow-up duration | Intervation | Age (mean ± SD), years | Sex (Male /Female） | Diabetes duration  (mean ± SD), years | Disease Staging, years | Outcomes |
| --- | --- | --- | --- | --- | --- | --- | --- | --- | --- |
|  |  |  |  |  |  |  |  |  |  |
| 23 | Huan HM 2010 | Combination:20 | 4 weeks | HQ+ARB | 68.8±13.9 | 11/9 | 12.8±7.3 | Ⅲ | ③④⑧ |
|  |  | Control:20 |  | ARB | 69.1±15.4 | 10/10 | 13.5±6.9 |  |  |
| 24 | Geng F 2010 | Combination:30 | 4 weeks | HQ+ACEI | 57±9 | 12/18 | 5.7±2.0 | Ⅲ | ③④⑥⑦ |
|  |  | Control:30 |  | ACEI | 59±12 | 14/16 | 6.7±1.9 |  |  |
| 25 | Xu ZX 2009 | Combination: 28 | 4 weeks | HQ+AEB | 49.84±13.1 | 13/11 | 12.3±6.8 | Ⅲ | ②③④⑤⑥⑦⑧ |
|  |  | Control:28 |  | ARB | 48.6±13.7） | 14/14 | 14.6±7.8 |  |  |
| 26 | Tian PW 2009 | Combination:40 | 8 weeks | HQ+ACEI | 50.4±7.2 | 19/21 | 8.2±3.7 | NA | ②⑤ |
|  |  | Control:40 |  | ACEI | 49.1±7.0 | 20/20 | 7.9±3.5 |  |  |
| 27 | Cui BS 2009 | Combination:36 | 4 weeks | HQ+ACEI | 50.8±11.9 | 24/12 | 4.8±2.6 | Ⅲ | ②③④⑧ |
|  |  | Control Group:36 |  | ACEI | 51.6±11.2 | 25/11 | 4.6±2.6 |  |  |
| 28 | Xu XL 2008 | Combination:38 | 4 weeks | HQ+ARB | 48.7±12.8 | 25/17 | 11.5±8.1 | Ⅲ | ②③④⑥⑦ |
|  |  | Control:42 |  | ARB | 49.8±11.9 | 22/16 | 10.7±8.5 |  |  |
| 29 | Chen JN 2010 | Combination:58 | 4 weeks | HQ+ARB | 46.3±11.7 | 30/28 | 4.8±3.4 | Ⅲ | ③④ |
|  |  | Control:54 |  | ARB | 47.2±11.9 | 28/26 | 4.7±2.6 |  |  |
| 30 | Jiang L 2005 | Combination:32 | 4 weeks | HQ+ACEI | 56.91±10.20 | 18/14 | 5.86±1.91 | NA | ②③④⑧ |
|  |  | Control:30 |  | ACEI | 57.33±9.74 | 16/14 | 5.73±2.20 |  |  |
| 31 | Li HJ 2007 | Combination:42 | 4 weeks | HQ+ACEI | NA | 42/26 | NA | Ⅲ | ②③④⑧ |
|  |  | Control:34 |  | ACEI | NA | 32/23 | NA |  |  |
| 32 | Huang W 2004 | Combination:30 | 4 weeks | HQ+ACEI | 56.6±10 | 16/14 | 5.6±1.7 | Ⅲ | ③④⑧ |
|  |  | Control:28 |  | ACEI | 58.9±9.9 | 18/10 | 5.8±2.0 |  |  |
| 33 | Ma CH 2006 | Combination:84 | 4 weeks | HQ+ACEI | 51.8±9.6 | 50/34 | 6.2±2.3 | NA | ②③④⑧ |
|  |  | Control:84 |  | ACEI | 54.6±9.2 | 48/36 | 6.4±2.7 |  |  |
| 34 | Shi XH 2010 | Combination:31 | 4 weeks | YX+ACEI | 50 | 18/13 | NA | Ⅲ | ②③④ |
|  |  | Control:31 |  | ACEI | 48 | 17/14 | NA |  |  |
| 35 | Zhang CJ 2013 | Combination:24 | 4 weeks | YX+ARB | 48.6±11.8 | 13/11 | 6.5±3.5 | Ⅰ,Ⅱ,Ⅲ | ①②③④⑦⑧ |
|  |  | Control:24 |  | ARB | 48.8±12.4 | 14/10 | 6.4±3.2 |  |  |
| 36 | Wang GC 2012 | Combination:89 | 8 weeks | YX+ARB | 57.95±12.63 | 47/42 | 12.53±5.85 | Ⅲ | ① |
|  |  | Control:89 |  | ARB | 56.14±12.52 | 45/44 | 12.58±5.57 |  |  |
| 37 | Chen JS 2015 | Combination:43 | 3 weeks | YX+ARB | 42.3±2.9 | 21/22 | 5.9±0.7 | NA | ①②③④ |
|  |  | Control:42 |  | ARB | 44±3 | 20/22 | 6.1±0.5 |  |  |
| 38 | Wang QZ 2011 | Combination:34 | 6 weeks | YX+ARB | 52.97±4.08 | 21/13 | NA | Ⅲ | ②⑤ |
|  |  | Control:34 |  | ARB | 54.58±4.98 | 22/12 | NA |  |  |
| 39 | Xing LY 2022 | Combination:98 | 15 days | YX+ARB | 56.39±4.55 | 52/46 | 4.23±1.28 | NA | ①② |
|  |  | Control:98 |  | ARB | 56.37±4.21 | 51/47 | 4.28±1.30 |  |  |
| 40 | Shen SL 2017 | Combination:30 | 3 weeks | YX+ACEI | 66.49±8.62 | 17/13 | 8.34±4.22 | Ⅲ | ②③④⑤ |
|  |  | Control:30 |  | ACEI | 65.51±8.38 | 21/9 | 8.93±4.15 |  |  |
| 41 | Zhang YM 2015 | Combination:68 | 4 weeks | YX+ARB | 57.7±4.2 | 35/33 | 6.7±3.1 | NA | ①②④⑤⑥⑦ |
|  |  | Control:68 |  | ARB | 58±4 | 37/31 | 6.3±2.7 |  |  |
| 42 | Li H 2014 | Combination:12 | 3 weeks | YX+ARB | 55.2±15.7 | 6/6 | 6.3±3.6 | NA | ①④⑥⑦ |
|  |  | Control:12 |  | ARB | 56.3±14.5 | 7/5 |  |  |  |
| 43 | Liu YX 2019 | Combination:31 | 12 weeks | YX+ARB | 57.2±5.0 | 16/15 | 5.4±1.6 | NA | ②④ |
|  |  | Control:31 |  | ARB | 56.9±5.2 | 17/14 | 5.6±1.5 |  |  |
| 44 | Huang JY 2017 | Combination:118 | 8 weeks | YX+ARB | 56.8±3.4 | 63/55 | 11.6±2.8 | NA | ①③④⑥ |
|  |  | Control:118 |  | ARB | 55.4±3.7 | 60/58 | 10.7±2.9 |  |  |

| Number | Study | Group (n) | Follow-up duration | Intervation | Age (mean ± SD), years | Sex (Male /Female） | Diabetes duration  (mean ± SD), years | Disease Staging, years | Outcomes |
| --- | --- | --- | --- | --- | --- | --- | --- | --- | --- |
|  |  |  |  |  |  |  |  |  |  |
| 45 | Cheng H 2018 | Combination:68 | 4 weeks | YX+ARB | 45.3±11.2 | 32/36 | NA | Ⅲ | ②④⑧ |
|  |  | Control:57 |  | ARB | 48.2±10.5 | 26/31 | NA |  |  |
| 46 | Zhu J 2020 | Combination:30 | 30 days | YX+ARB | 60.3±7.2 | 10/20 | 8.3±1.6 | NA | ①③④ |
|  |  | Control:30 |  | ARB | 60.5±7.3 | 12/18 | 8.4±1.7 |  |  |
| 47 | You W 2014 | Combination:31 | 4 weeks | YX+ARB | 51.8±6.2 | 16/15 | NA | NA | ③④ |
|  |  | Control:26 |  | ARB | 51.5±7.6 | 14/12 | NA |  |  |
| 48 | Li L 2012 | Combination:29 | 12 weeks | YX+ARB | 51.2±8.6 | 17/12 | 7.3±4.2 | Ⅲ | ②③④ |
|  |  | Control:31 |  | ARB | 53.4±9.1 | 19/12 | 8.0±5.1 |  |  |
| 49 | Li SH 2009 | Combination:80 | 4 weeks | YX+ACEI | 52.6±7.6 | 56/24 | 9.2±7.2 | Ⅲ | ②③④⑥⑦ |
|  |  | Control:80 |  | ACEI | 51.8±6.3 | 46/34 | 9.3±6.9 |  |  |
| 50 | Li ZY 2013 | Combination:49 | 4 weeks | YX+ARB | 46.13±9.12 | 26/23 | 16.28±2.61 | NA | ③④⑧ |
|  |  | Control:48 |  | ARB | 45.73±8.65 | 25/23 | 6.20±2.59 |  |  |
| 51 | Wu GY 2017 | Combination:30 | 4 weeks | SX+ACEI | 57.6±5.1 | 20/10 | 10.3±2.4 | NA | ①④⑤ |
|  |  | Control:30 |  | ACEI | 56.9±5.3 | 18/12 | 11.01±2.5 |  |  |
| 52 | Yang QH 2007 | Combination:35 | 4 weeks | SX+ACEI | NA | 19/16 | NA | NA | ①③④ |
|  |  | Control:25 |  | ACEI | NA | 13/12 | NA |  |  |
| 53 | Xu YY 2020 | Combination:43 | 8 weeks | SX+ARB | 57.84±7.29 | 22/21 | 4.11±1.80 | NA | ③④ |
|  |  | Control:43 |  | ARB | 58.07±7.15 | 23/20 | 9.56±2.26 |  |  |
| 54 | Zhen D 2012 | Combination:36 | 4 weeks | SX+ARB | NA | 21/15 | NA | NA | ②③④⑥⑦⑧ |
|  |  | Control:36 |  | ARB | NA | 19/17 | NA |  |  |
| 55 | Geng GM 2013 | Combination:38 | 2 weeks | DC+ARB | 56.7±10.8 | 20/18 | 12.9±6.9 | Ⅲ,Ⅳ | ③④⑥⑦⑧ |
|  |  | Control:38 |  | ARB | 57.2±9.7 | 20/18 | 8.6±5.7 |  |  |
| 56 | Tang N 2019 | Combination:97 | 2 weeks | DC+ACEI | 54.43±5.31 | 52/45 | 6.32±1.21 | NA | ①③④⑦ |
|  |  | Control:83 |  | ACEI | 55.02±5.37 | 46/37 | 6.51±1.27 |  |  |
| 57 | Gong J 2022 | Combination:63 | 2 weeks | DC+ACEI | 55.72±2.69 | 33/30 | 1.81±0.29 | Ⅰ,Ⅱ,Ⅲ,Ⅳ | ③④⑥⑦ |
|  |  | Control:63 |  | ACEI | 55.36±2.82 | 34/29 | 1.73±0.36 |  |  |
| 58 | Xian LH 2015 | Combination:38 | 4 weeks | DC+ARB | 59.7±10.5 | 25/13 | 8.7±2.6 | Ⅲ | ② |
|  |  | Control:38 |  | ARB | 58.3±11.8 | 24/14 | 9.3±3.2 |  |  |
| 59 | Zhao Y 2014 | Combination:40 | 4 weeks | DC+ARB | 60.53±17.45 | 16/24 | 10.42±3.18 | Ⅲ | ③④⑤ |
|  |  | Control:40 |  | ARB | 62.13±18.17 | 18/22 | 11.08±3.76 |  |  |
| 60 | Tao L 2015 | Combination:33 | 2 weeks | DC+ACEI | 53.5±5.8 | 20/13 | 8.7±7.1 | NA | ②⑤ |
|  |  | Control:33 |  | ACEI | 54.9±6.1 | 18/15 | 8.9±6.8 |  |  |
| 61 | Tao Y 2016 | Combination:80 | 4 weeks | DC+ARB | 57.8±4.9 | 44/36 | 10.8±2.4 | Ⅲ | ①③⑧ |
|  |  | Control:80 |  | ARB | 57.2±5.3 | 43/37 | 10.3±2.3 |  |  |
| 62 | Zhang ZM 2014 | Combination:40 | 2 weeks | DC+ARB | 56.9±5.4 | 17/23 | NA | Ⅲ,Ⅳ | ① |
|  |  | Control:40 |  | ARB | 59.2±6.4 | 16/24 | NA |  |  |
| 63 | Fan WK 2017 | Combination:75 | 4 weeks | DC+ARB | 51.86±15.17 | 43/32 | 14.59±6.74 | Ⅲ | ①③④ |
|  |  | Control:75 |  | ARB | 52.34±14.65 | 41/34 | 14.36±6.56 |  |  |
| 64 | Zhou AJ 2009 | Combination:30 | 30 days | DS+ARB | 56.22±3.12 | 18/12 | NA | NA | ②⑤⑧ |
|  |  | Control:30 |  | ARB | 55.31±2.83 | 20/10 | NA |  |  |
| 65 | Wei HB 2016 | Combination:51 | 8 weeks | DS+ARB | 53.9±10.7 | 28/23 | 4.6±1.1 | Ⅲ | ①③④ |
|  |  | Control:51 |  | ARB | 54.5±10.2 | 25/26 | 4.7±1.4 |  |  |
| 66 | Hu B 2016 | Combination:184 | 4 weeks | DS+ACEI | 59.14±9.01 | 100/84 | NA | Ⅳ | ④⑥⑦⑧ |
|  |  | Control:184 |  | ACEI | 59.03±8.73 | 98/96 | NA |  |  |

| Number | Study | Group (n) | Follow-up duration | Intervation | Age (mean ± SD), years | Sex (Male /Female） | Diabetes duration  (mean ± SD), years | Disease Staging, years | Outcomes |
| --- | --- | --- | --- | --- | --- | --- | --- | --- | --- |
|  |  |  |  |  |  |  |  |  |  |
| 67 | Xian XW 2014 | Combination:50 | 6 weeks | DS+ACEI | 59.3±6.4 | 32/18 | NA | NA | ①③④ |
|  |  | Control:50 |  | ACEI | 58.8±5.9 | 31/19 | NA |  |  |
| 68 | Tian YH 2015 | Combination:39 | 4 weeks | DS+ARB | 53.9±4.6 | 22/17 | 5.1±3.3 | NA | ① |
|  |  | Control:38 |  | ARB | 54.3±4.8 | 23/15 | 5.3±4.1 |  |  |
| 69 | Li QY 2011 | Combination:40 | 60days | DH+ARB | 60±6 | 26/14 | 8.8±0.9 | NA | ④ |
|  |  | Control:40 |  | ARB | 56±7 | 27/13 | 8.6±1.1 |  |  |
| 70 | Wang CQ 2012 | Combination:30 | 2 weeks | DH+ACEI | 41.6±12.1 | 14/16 | NA | Ⅲ | ②⑤⑦ |
|  |  | Control:30 |  | ACEI | 40.8±12.1 | 13/17 | NA |  |  |
| 71 | Huang YJ 2016 | Combination:48 | 2 weeks | DH+ARB | 61.3±7.4 | 27/21 | 7.9±3.2 | Ⅲ | ③④⑤ |
|  |  | Control:48 |  | ARB | 59.1±6.8 | 28/20 | 8.3 ±2.9 |  |  |
| 72 | Ye XA 2016 | Combination:51 | 36 weeks | DH+ARB | 54.3±11.5 | 26/25 | 9.2±3.4 | Ⅲ | ②③④⑤ |
|  |  | Control:51 |  | ARB | 54.6±11.6 | 27/24 | 9.5 ±3.8 |  |  |
| 73 | Li JJ 2009 | Combination:42 | 2 weeks | DH+ARB | 58.2±8.7 | 24/18 | NA | Ⅲ | ②③④ |
|  |  | Control:40 |  | ARB | 59.2±8.1 | 27/24 | NA |  |  |
| 74 | Liu W 2009 | Combination:66 | 4 weeks | DH+ACEI | 54.3±9.1 | 38/28 | 6.8±2.5 | NA | ①③④ |
|  |  | Control:60 |  | ACEI | 51.8±9.5 | 34/26 | 6.2±2.3 |  |  |
| 75 | Jing XN 2014 | Combination:61 | 4 weeks | DH+ACEI | 45.3±4.9 | 36/25 | 12±9 | NA | ③④⑥⑦ |
|  |  | Control:61 |  | ACEI | 42.4±5.7 | 33/28 | 11±8 |  |  |
| 76 | Tao GL 2019 | Combination:30 | 4 weeks | SK+ARB | 54.5±14.9 | 15/15 | 10.5±1.9 | Ⅲ | ①③④⑥⑦ |
|  |  | Control:30 |  | ARB | 52.8±16.3 | 13/17 | 9.7±3.2 |  |  |
| 77 | He LF 2020 | Combination:80 | 4 weeks | SK+ARB | 49.3士9.0 | 45/35 | 9.0士1.2 | Ⅳ | ④ |
|  |  | Control:80 |  | ARB | 48.5士8.5 | 48/32 | 8.5±1.1 |  |  |
| 78 | Hao JL 2022 | Combination:160 | 4 weeks | SK+ARB | 54.6±5.2 | 90/70 | 4.2±1.1 | Ⅰ,Ⅱ,Ⅲ | ①④⑤ |
|  |  | Control:160 |  | ARB | 54.2±5.4 | 88/22 | 4.5±1.3 |  |  |
| 79 | Wang AY 2013 | Combination:33 | 4 weeks | SK+ACEI | 59.2±8.6 | 19/14 | 8.7±6.4 | Ⅲ | ⑤ |
|  |  | Control:33 |  | ACEI | 60.5±8.8 | 18/15 | 8.1±7.3 |  |  |
| 80 | Ren LL 2018 | Combination:50 | 3 weeks | SK+ARB | 57.13±5.86 | 27/23 | 6.02±2.13 | NA | ①③④⑤ |
|  |  | Control:50 |  | ARB | 60.5±8.8 | 25/25 | 5.79±2.06 |  |  |
| 81 | Li HF 2022 | Combination:44 | 12 weeks | SK+ARB | 57.5±15.2 | 24/20 | 6.02±2.13 | NA | ②③④⑥⑦ |
|  |  | Control:44 |  | ARB | 57.7±15.0 | 22/22 | 5.79±2.06 |  |  |
| 82 | Xiao L 2013 | Combination:34 | 8 weeks | SK+ARB | 57.2±14.9 | 17/17 | 10.1±2.4 | Ⅳ | ⑤⑥⑦ |
|  |  | Control:34 |  | ARB | 57.6±15.2 | 19/15 |  |  |  |
| 83 | Deng ZH 2021 | Combination:91 | 12 weeks | SK+ARB | 52.69±9.11 | 52/39 | 9.10±3.26 | Ⅰ,Ⅱ,Ⅲ | ①③④ |
|  |  | Control:91 |  | ARB | 50.42±8.37 | 49/42 | 9.45±2.51 |  |  |
| 84 | Hu YL 2015 | Combination:36 | 4 weeks | SK+ARB | 60.4±5.7 | 20/16 | 6.1±2.5 | Ⅲ | ②③④ |
|  |  | Control:36 |  | ARB | 58.2±5.7 | 18/18 | 5.8±2.7 |  |  |
| 85 | Ma YQ 2019 | Combination:45 | 4 weeks | SK+ARB | 56.56±5.38 | 28/17 | 4.26±1.20 | Ⅰ,Ⅱ,Ⅲ | ①④ |
|  |  | Control:45 |  | ACEI | 56.61±5.69 | 27/18 | 4.24±1.27 |  |  |
| 86 | Chen P 2021 | Combination:34 | 4 weeks | SK+ARB | 56.39±3.11 | 19/15 | NA | NA | ③④ |
|  |  | Control:34 |  | ARB | 57.92±3.29 | 20/14 | NA |  |  |
| 87 | Song LH 2012 | Combination:43 | 4 weeks | SK+ARB | 54.7±5.4 | 23/20 | 9.2±1.4 | Ⅳ | ⑥⑦⑧ |
|  |  | Control:35 |  | ARB | 53.9±8.6 | 19/16 | 9.7±1.8 |  |  |
| 88 | Rao GF 2013 | Combination:90 | 4 weeks | KD+ARB | 51.2±6.9 | 45/45 | 8.3±4.5 | Ⅲ | ①②③④⑤ |
|  |  | Control:90 |  | ARB | 50.3±7.3 | 47/43 | 8.5±4.3 |  |  |

| Number | Study | Group (n) | Follow-up duration | Intervation | Age (mean ± SD), years | Sex (Male /Female） | Diabetes duration  (mean ± SD), years | Disease Staging, years | Outcomes |
| --- | --- | --- | --- | --- | --- | --- | --- | --- | --- |
|  |  |  |  |  |  |  |  |  |  |
| 89 | Jin QJ 2016 | Combination:179 | 4 weeks | KD+ARB | 49.23±3.24 | 104/75 | 8.45±1.78 | NA | ①③④⑤ |
|  |  | Control:179 |  | ARB | 47.86±3.15 | 98/81 | 8.52±1.59 |  |  |
| 90 | Zheng JJ 2013 | Combination:96 | 4 weeks | KD+ACEI | 55±7 | 54/42 | NA | Ⅲ,Ⅳ | ①⑤ |
|  |  | Control:96 |  | ACEI | 58±6 | 56/60 | NA |  |  |
| 91 | Zhang SJ 2023 | Combination:50 | 2 weeks | KD+ACEI/ARB | 56.97±8.15 | 29/21 | 8.45±1.78 | Ⅱ,Ⅲ,Ⅳ | ①②③④ |
|  |  | Control:50 |  | ACEI/ARB | 58.02±7.43 | 26/24 | 8.52±1.59 |  |  |
| 92 | Li HF 2010 | Combination:30 | 4 weeks | ST+ACEI | 52.4±4.8 | 16/14 | 11.4±2.6 | Ⅲ | ②③④⑤ |
|  |  | Control:30 |  | ACEI | 46.8±4.5 | 12/18 | 13.1±2.5 |  |  |
| 93 | Sun XP 2015 | Combination:35 | 8 weeks | ST+ARB | 75.2±5.8 | 17/18 | 8.3±3.1 | Ⅲ | ②④ |
|  |  | Control:35 |  | ARB | 75.8±5.2 | 18/17 | 8.1±3.3 |  |  |
| 94 | Li B 2013 | Combination:69 | 12 weeks | ST+ACEI | 35.2±3.4 | 37/32 | 18.6±2.4 | Ⅲ,Ⅳ | ①⑥⑦ |
|  |  | Control:65 |  | ACEI | 36.5±3.1 | 34/31 | 18.3±3.0 |  |  |
| 95 | Qin WG 2010 | Combination:40 | 8 weeks | ST+ACEI | 62.4±9.7 | 28/12 | 8.95±1.95 | Ⅲ | ②③④ |
|  |  | Control:40 |  | ACEI | 63.2±9.8 | 27/13 | 8.67±2.31 |  |  |
| 96 | Zhu YX 2003 | Combination:30 | 2 weeks | GG+ACEI | 66.43±9.56 | 16/14 | 13.6±7.59 | Ⅲ,Ⅳ | ② |
|  |  | Control:30 |  | ACEI | 65.90±9.94 | 14/16 | 13.6±7.75 |  |  |
| 97 | Xu JQ 2011 | Combination:22 | 4 weeks | GG+ACEI | 52.2±8.7 | 14/8 | 10.8±3.1 | NA | ②③④⑥ |
|  |  | Control:18 |  | ACEI | 50.3±8.5 | 11/7 | 11.5±3.4 |  |  |
| 98 | Yuan DY 2009 | Combination:42 | 2 weeks | GG+ACEI | 51.2±8.8 | 19/23 | 7.2±3.5 | Ⅲ | ③④⑥⑦ |
|  |  | Control:42 |  | ACEI | 51.8±8.4 | 20/22 | 7.7±3.9 |  |  |
| 99 | Jiao FZ 2011 | Combination:54 | 30 days | GG+ACEI | 51.2±7.6 | 29/25 | 6.7±3.2 | Ⅲ | ②③④ |
|  |  | Control:42 |  | ACEI | 50.7±7.9 | 22/20 | 7.1±2.8 |  |  |

①total effective rate; ②urinary albumin excretion rate; ③blood urea nitrogen; ④serum creatinine; ⑤β_2_-microglobulin; ⑥total cholesterol; ⑦triglyceride; ⑧systolic blood pressure; ACEI, angiotensin-converting enzyme inhibitor; ARB, angiotensin Ⅱ receptor blockers;

Appendix Table A.16: Risk of Bias of Included Studies

| Study ID | Randomization  process | Deviations from intended interventions | Missing outcome data | Measurement of the outcome | Selection of the reported result | Overall Bias |
| --- | --- | --- | --- | --- | --- | --- |
| Zhang YX  2021 | Low | Low | Low | Low | Low | Low |
| Zhang SB  2020 | Low | Low | Low | Low | Low | Low |
| Qiao MF  2018 | Some  concerns | Low | Low | Low | Low | Some  concerns |
| Zhang X  2017 | Some  concerns | Low | Low | Low | Low | Some  concerns |
| Li H  2017 | Some  concerns | Low | Low | Low | Low | Some  concerns |
| Tang H  2016 | Low | Low | Low | Low | Low | Low |
| Ren YJ  2016 | Some  concerns | Low | Low | Low | Low | Some  concerns |
| Liu SY  2016 | Some  concerns | Some  concerns | Low | Low | Low | Some  concerns |
| Cai R  2016 | Low | Low | Low | Low | Low | Low |
| Wang XH 2015 | Some  concerns | Low | Low | Low | Low | Some  concerns |
| Liu XX  2015 | Low | Low | Low | Low | Low | Low |
| Liu S  2015 | Some  concerns | Low | Low | Low | Low | Some  concerns |
| Li SB  2015 | Some  concerns | Low | Some  concerns | Low | Low | Some  concerns |
| Zhang Q  2014 | Low | Low | Low | Low | Low | Low |
| Sun YH  2014 | Some  concerns | Some  concerns | Low | Low | Low | Some  concerns |
| Liu XX  2013 | Some concerns | Low | Low | Low | Low | Some  concerns |
| Huang SL  2013 | Some  concerns | Some  concerns | Low | Low | Low | Some  concerns |
| Zhang Y  2012 | Some  concerns | Some  concerns | Low | Low | Low | Some  concerns |
| Zeng J 2012 | Some  concerns | Some  concerns | Low | Low | Low | Some  concerns |
| Zhu LY  2011 | Some  concerns | Some  concerns | Low | Low | Low | Some  concerns |
| Zeng J  2010 | Some  concerns | Some  concerns | Low | Low | Low | Some  concerns |
| Li ZQ  2010 | Some  concerns | Some  concerns | Low | Low | Low | Some  concerns |
| Huan HM  2010 | Some  concerns | Some  concerns | Low | Low | Low | Some  concerns |
| Geng F  2010 | Low | Low | Low | Low | Low | Low |
| Xu ZX  2009 | Some  concerns | Some  concerns | Low | Low | Some  concerns | Some  concerns |
| Tian PW  2009 | Some  concerns | Some  concerns | Low | Low | Some  concerns | Some  concerns |
| Cui BS  2009 | Some  concerns | Some  concerns | Low | Low | Some  concerns | Some  concerns |
| Xu XL  2008 | Low | Low | Low | Low | Low | Low |
| Chen JN  2010 | Some  concerns | Some  concerns | Low | Low | Low | Some  concerns |
| Jiang Lan  2005 | Some  concerns | Some  concerns | Low | Low | Some  concerns | Some  concerns |
| Li HJ  2007 | Some  concerns | Some  concerns | Low | Low | Some  concerns | Some  concerns |
| Huang W  2004 | Some  concerns | Some  concerns | Low | Low | Some  concerns | Some  concerns |
| Ma CH  2006 | Some  concerns | Some  concerns | Low | Low | Some  concerns | Some  concerns |
| Shi XH  2010 | Some  concerns | Some  concerns | Low | Low | Low | Some  concerns |
| Zhang CJ  2013 | Some  concerns | Some  concerns | Low | Low | Low | Some  concerns |
| Wang GC  2012 | Low | Low | Some  concerns | Low | Low | Some  concerns |
| Chen JS  2015 | Low | Low | Low | Low | Low | Low |
| Wang Qz  2011 | Some  concerns | Some  concerns | Low | Low | Low | Some  concerns |
| Xing LY  2022 | Low | Low | Low | Low | Low | Low |
| Shen SL  2017 | Some  concerns | Low | Low | Low | Low | Some  concerns |
| Zhang YM  2015 | Some  concerns | Some  concerns | Low | Low | Low | Some  concerns |
| Li Hong  2014 | Some  concerns | Some  concerns | Low | Low | Low | Some  concerns |
| Liu YX  2019 | Low | Low | Low | Low | Low | Low |
| Huang JY  2017 | Low | Low | Low | Low | Low | Low |
| Cheng H  2018 | Low | Low | Low | Low | Low | Low |
| Zhu J  2020 | Low | Low | Low | Low | Low | Low |
| You Wen  2014 | Low | Low | Low | Low | Low | Low |
| Li L  2012 | Some  concerns | Some  concerns | Low | Low | Low | Some  concerns |
| Li SH  2009 | Some  concerns | Some  concerns | Low | Low | Some  concerns | Some  concerns |
| Li ZY  2013 | Some  concerns | Some  concerns | Low | Low | Low | Some  concerns |
| Wu GY  2017 | Low | Low | Low | Low | Low | Low |
| Yang QH  2007 | Some  concerns | Some  concerns | Low | Low | Some  concerns | Some  concerns |
| Xu YY  2020 | Low | Low | Low | Low | Low | Low |
| Zhen D  2012 | Some  concerns | Some  concerns | Low | Low | Low | Some  concerns |
| Geng GM  2013 | Some  concerns | Some  concerns | Low | Low | Low | Some  concerns |
| Tang N  2019 | Low | Low | Low | Low | Low | Low |
| Gong J  2022 | Low | Low | Low | Low | Low | Low |
| Xian LH  2015 | Some  concerns | Some  concerns | Low | Low | Low | Some  concerns |
| Zhang Y  2014 | Some  concerns | Some  concerns | Low | Low | Low | Some  concerns |
| Tao L  2015 | Some  concerns | Low | Low | Low | Low | Some  concerns |
| Tao Y  2016 | Low | Low | Low | Low | Low | Low |
| Zhang ZM  2014 | Some  concerns | Some  concerns | Some  concerns | Low | Low | Some  concerns |
| Fan WK  2017 | Low | Low | Low | Low | Low | Low |
| Zhou AJ  2009 | Some  concerns | Some  concerns | Low | Low | Some  concerns | Some  concerns |
| Wei HB  2016 | Some  concerns | Some  concerns | Low | Low | Low | Some  concerns |
| Hu B  2016 | Low | Low | Low | Low | Low | Low |
| Xian XW  2014 | Low | Low | Low | Low | Low | Low |
| Tian YH  2015 | Some  concerns | Some  concerns | Low | Low | Low | Some  concerns |
| Li QY  2011 | Some  concerns | Some  concerns | Low | Low | Low | Some  concerns |
| Wang CQ  2012 | Some  concerns | Some  concerns | Low | Low | Low | Some  concerns |
| Huang YJ  2016 | Low | Low | Low | Low | Low | Some  concerns |
| Ye XA  2016 | Some  concerns | Some  concerns | Low | Low | Low | Some  concerns |
| Li JJ  2009 | Some  concerns | Some  concerns | Low | Low | Some  concerns | Some  concerns |
| Liu W  2009 | Some  concerns | Some  concerns | Low | Low | Some  concerns | Some  concerns |
| Jing XN  2014 | Some  concerns | Some  concerns | Low | Low | Low | Some  concerns |
| Tao GL  2019 | Some  concerns | Low | Low | Low | Low | Some  concerns |
| He LF  2020 | Low | Low | Low | Low | Low | Low |
| Hao JL  2022 | Low | Low | Low | Low | Low | Low |
| Wang AY 2013 | Low | Low | Low | Low | Low | Low |
| Ren LL  2018 | Low | Low | Low | Low | Low | Low |
| Li HF  2022 | Low | Low | Low | Low | Low | Low |
| Xiao L  2013 | Some  concerns | Some  concerns | Low | Low | Low | Some  concerns |
| Deng ZH  2021 | Low | Low | Low | Low | Low | Low |
| Hu YL  2015 | Some  concerns | Some  concerns | Low | Low | Low | Some  concerns |
| Ma YQ  2019 | Low | Low | Low | Low | Low | Low |
| Chen P  2021 | Some  concerns | Low | Low | Low | Low | Some  concerns |
| Song LH  2012 | Some  concerns | Some  concerns | Low | Low | Low | Some  concerns |
| Rao GF  2013 | Low | Low | Low | Low | Low | Low |
| Jin QJ  2016 | Low | Low | Low | Low | Low | Low |
| Zheng JJ  2013 | Low | Low | Low | Low | Low | Low |
| Zhang SJ  2023 | Low | Low | Low | Low | Low | Low |
| Li HF  2010 | Some  concerns | Some  concerns | Low | Low | Low | Some  concerns |
| Sun XP  2015 | Some  concerns | Some  concerns | Low | Low | Low | Some  concerns |
| Li B  2013 | Some  concerns | Some  concerns | Low | Low | Low | Some  concerns |
| Qin WG  2010 | Some  concerns | Some  concerns | Low | Low | Low | Some  concerns |
| Zhu YX  2003 | Some  concerns | Some  concerns | Low | Low | Some  concerns | Some  concerns |
| Xu JQ  2011 | Some  concerns | Some  concerns | Low | Low | Low | Some  concerns |
| Yuan DY  2009 | Low | Low | Low | Low | Some  concerns | Some  concerns |
| Jiao FZ  2011 | Some  concerns | Some  concerns | Low | Low | Low | Some  concerns |

Appendix Table A.17-A.24: GRADE assessment

Table A.17: GRADE assessment: UAER

| Comparison | Number of studies | Within-study bias | Reporting bias | Indirectness | Imprecision | Heterogeneity | Incoherence | Confidence rating | Reason(s) for downgrading |
| --- | --- | --- | --- | --- | --- | --- | --- | --- | --- |
| A:B | 21 | Some concerns | Low risk | No concerns | No concerns | Major concerns | Some concerns | Very low | ["Within-study bias","Heterogeneity","Incoherence"] |
| A:C | 11 | Some concerns | Low risk | No concerns | No concerns | Major concerns | Some concerns | Very low | ["Within-study bias","Heterogeneity","Incoherence"] |
| A:D | 1 | Some concerns | Low risk | No concerns | Major concerns | No concerns | Some concerns | Very low | ["Within-study bias","Imprecision","Incoherence"] |
| A:E | 2 | Some concerns | Low risk | No concerns | Major concerns | No concerns | Some concerns | Very low | ["Within-study bias","Imprecision","Incoherence"] |
| A:F | 1 | Some concerns | Low risk | No concerns | No concerns | Major concerns | Some concerns | Very low | ["Within-study bias","Heterogeneity","Incoherence"] |
| A:G | 3 | Some concerns | Low risk | No concerns | No concerns | Major concerns | Some concerns | Very low | ["Within-study bias","Heterogeneity","Incoherence"] |
| A:H | 2 | No concerns | Low risk | No concerns | Major concerns | No concerns | Some concerns | Low | ["Imprecision","Incoherence"] |
| A:I | 2 | No concerns | Low risk | No concerns | Major concerns | No concerns | Some concerns | Low | ["Imprecision","Incoherence"] |
| A:J | 3 | Some concerns | Low risk | No concerns | No concerns | No concerns | Some concerns | Very low | ["Within-study bias","Incoherence"] |
| A:K | 3 | Some concerns | Low risk | No concerns | Major concerns | No concerns | Some concerns | Very low | ["Within-study bias","Imprecision","Incoherence"] |
| B:C | 0 | Some concerns | Low risk | No concerns | Major concerns | No concerns | Some concerns | Very low | ["Within-study bias","Imprecision","Incoherence"] |
| B:D | 0 | Some concerns | Low risk | No concerns | Major concerns | No concerns | Some concerns | Very low | ["Within-study bias","Imprecision","Incoherence"] |
| B:E | 0 | Some concerns | Low risk | No concerns | Major concerns | No concerns | Some concerns | Very low | ["Within-study bias","Imprecision","Incoherence"] |
| B:F | 0 | Some concerns | Low risk | No concerns | Major concerns | No concerns | Some concerns | Very low | ["Within-study bias","Imprecision","Incoherence"] |
| B:G | 0 | Some concerns | Low risk | No concerns | Major concerns | No concerns | Some concerns | Very low | ["Within-study bias","Imprecision","Incoherence"] |
| B:H | 0 | Some concerns | Low risk | No concerns | Major concerns | No concerns | Some concerns | Very low | ["Within-study bias","Imprecision","Incoherence"] |
| B:I | 0 | No concerns | Low risk | No concerns | Major concerns | No concerns | Some concerns | Low | ["Imprecision","Incoherence"] |
| B:J | 0 | Some concerns | Low risk | No concerns | Major concerns | No concerns | Some concerns | Very low | ["Within-study bias","Imprecision","Incoherence"] |
| B:K | 0 | Some concerns | Low risk | No concerns | Major concerns | No concerns | Some concerns | Very low | ["Within-study bias","Imprecision","Incoherence"] |
| C:D | 0 | Some concerns | Low risk | No concerns | Major concerns | No concerns | Some concerns | Very low | ["Within-study bias","Imprecision","Incoherence"] |
| C:E | 0 | Some concerns | Low risk | No concerns | Major concerns | No concerns | Some concerns | Very low | ["Within-study bias","Imprecision","Incoherence"] |
| C:F | 0 | Some concerns | Low risk | No concerns | Major concerns | No concerns | Some concerns | Very low | ["Within-study bias","Imprecision","Incoherence"] |
| C:G | 0 | Some concerns | Low risk | No concerns | Major concerns | No concerns | Some concerns | Very low | ["Within-study bias","Imprecision","Incoherence"] |
| C:H | 0 | Some concerns | Low risk | No concerns | Major concerns | No concerns | Some concerns | Very low | ["Within-study bias","Imprecision","Incoherence"] |
| C:I | 0 | No concerns | Low risk | No concerns | Major concerns | No concerns | Some concerns | Low | ["Imprecision","Incoherence"] |
| C:J | 0 | Some concerns | Low risk | No concerns | Major concerns | No concerns | Some concerns | Very low | ["Within-study bias","Imprecision","Incoherence"] |
| C:K | 0 | Some concerns | Low risk | No concerns | Major concerns | No concerns | Some concerns | Very low | ["Within-study bias","Imprecision","Incoherence"] |
| D:E | 0 | Some concerns | Low risk | No concerns | Major concerns | No concerns | Some concerns | Very low | ["Within-study bias","Imprecision","Incoherence"] |
| D:F | 0 | Some concerns | Low risk | No concerns | Major concerns | No concerns | Some concerns | Very low | ["Within-study bias","Imprecision","Incoherence"] |
| D:G | 0 | Some concerns | Low risk | No concerns | Major concerns | No concerns | Some concerns | Very low | ["Within-study bias","Imprecision","Incoherence"] |
| D:H | 0 | Some concerns | Low risk | No concerns | Major concerns | No concerns | Some concerns | Very low | ["Within-study bias","Imprecision","Incoherence"] |
| D:I | 0 | Some concerns | Low risk | No concerns | Major concerns | No concerns | Some concerns | Very low | ["Within-study bias","Imprecision","Incoherence"] |
| D:J | 0 | Some concerns | Low risk | No concerns | No concerns | Major concerns | Some concerns | Very low | ["Within-study bias","Incoherence"] |
| D:K | 0 | Some concerns | Low risk | No concerns | Major concerns | No concerns | Some concerns | Very low | ["Within-study bias","Imprecision","Incoherence"] |
| E:F | 0 | Some concerns | Low risk | No concerns | Major concerns | No concerns | Some concerns | Very low | ["Within-study bias","Imprecision","Incoherence"] |
| E:G | 0 | Some concerns | Low risk | No concerns | Major concerns | No concerns | Some concerns | Very low | ["Within-study bias","Imprecision","Incoherence"] |
| E:H | 0 | Some concerns | Low risk | No concerns | Major concerns | No concerns | Some concerns | Very low | ["Within-study bias","Imprecision","Incoherence"] |
| E:I | 0 | Some concerns | Low risk | No concerns | Major concerns | No concerns | Some concerns | Very low | ["Within-study bias","Imprecision","Incoherence"] |
| E:J | 0 | Some concerns | Low risk | No concerns | No concerns | Major concerns | Some concerns | Very low | ["Within-study bias","Heterogeneity","Incoherence"] |
| E:K | 0 | Some concerns | Low risk | No concerns | Major concerns | No concerns | Some concerns | Very low | ["Within-study bias","Imprecision","Incoherence"] |
| F:G | 0 | Some concerns | Low risk | No concerns | Major concerns | No concerns | Some concerns | Very low | ["Within-study bias","Imprecision","Incoherence"] |
| F:H | 0 | Some concerns | Low risk | No concerns | Major concerns | No concerns | Some concerns | Very low | ["Within-study bias","Imprecision","Incoherence"] |
| F:I | 0 | Some concerns | Low risk | No concerns | Major concerns | No concerns | Some concerns | Very low | ["Within-study bias","Imprecision","Incoherence"] |
| F:J | 0 | Some concerns | Low risk | No concerns | Major concerns | No concerns | Some concerns | Very low | ["Within-study bias","Imprecision","Incoherence"] |
| F:K | 0 | Some concerns | Low risk | No concerns | Major concerns | No concerns | Some concerns | Very low | ["Within-study bias","Imprecision","Incoherence"] |
| G:H | 0 | Some concerns | Low risk | No concerns | Major concerns | No concerns | Some concerns | Very low | ["Within-study bias","Imprecision","Incoherence"] |
| G:I | 0 | Some concerns | Low risk | No concerns | Major concerns | No concerns | Some concerns | Very low | ["Within-study bias","Imprecision","Incoherence"] |
| G:J | 0 | Some concerns | Low risk | No concerns | Major concerns | No concerns | Some concerns | Very low | ["Within-study bias","Imprecision","Incoherence"] |
| G:K | 0 | Some concerns | Low risk | No concerns | Major concerns | No concerns | Some concerns | Very low | ["Within-study bias","Imprecision","Incoherence"] |
| H:I | 0 | No concerns | Low risk | No concerns | Major concerns | No concerns | Some concerns | Low | ["Imprecision","Incoherence"] |
| H:J | 0 | Some concerns | Low risk | No concerns | Major concerns | No concerns | Some concerns | Very low | ["Within-study bias","Imprecision","Incoherence"] |
| H:K | 0 | Some concerns | Low risk | No concerns | Major concerns | No concerns | Some concerns | Very low | ["Within-study bias","Imprecision","Incoherence"] |
| I:J | 0 | Some concerns | Low risk | No concerns | Major concerns | No concerns | Some concerns | Very low | ["Within-study bias","Imprecision","Incoherence"] |
| I:K | 0 | Some concerns | Low risk | No concerns | Major concerns | No concerns | Some concerns | Very low | ["Within-study bias","Imprecision","Incoherence"] |
| J:K | 0 | Some concerns | Low risk | No concerns | Major concerns | No concerns | Some concerns | Very low | ["Within-study bias","Imprecision","Incoherence"] |

Table A.18: GRADE assessment: Scr

| Comparison | Number of studies | Within-study bias | Reporting bias | Indirectness | Imprecision | Heterogeneity | Incoherence | Confidence rating | Reason(s) for downgrading |
| --- | --- | --- | --- | --- | --- | --- | --- | --- | --- |
| A:B | 28 | Some concerns | Low risk | No concerns | No concerns | Major concerns | Some concerns | Very low | ["Within-study bias","Heterogeneity","Incoherence"] |
| A:C | 14 | Some concerns | Low risk | No concerns | Major concerns | No concerns | Some concerns | Very low | ["Within-study bias","Imprecision","Incoherence"] |
| A:D | 4 | No concerns | Low risk | No concerns | Major concerns | No concerns | Some concerns | Low | ["Imprecision","Incoherence"] |
| A:E | 5 | No concerns | Low risk | No concerns | No concerns | Major concerns | Some concerns | Low | ["Heterogeneity","Incoherence"] |
| A:F | 3 | No concerns | Low risk | No concerns | No concerns | Major concerns | Some concerns | Low | ["Heterogeneity","Incoherence"] |
| A:G | 6 | Some concerns | Low risk | No concerns | Major concerns | No concerns | Some concerns | Very low | ["Within-study bias","Imprecision","Incoherence"] |
| A:H | 9 | No concerns | Low risk | No concerns | No concerns | Major concerns | Some concerns | Low | ["Heterogeneity","Incoherence"] |
| A:I | 3 | No concerns | Low risk | No concerns | No concerns | Major concerns | Some concerns | Low | ["Heterogeneity","Incoherence"] |
| A:J | 3 | Some concerns | Low risk | No concerns | Major concerns | No concerns | Some concerns | Very low | ["Within-study bias","Imprecision","Incoherence"] |
| B:C | 0 | Some concerns | Low risk | No concerns | Major concerns | No concerns | Some concerns | Very low | ["Within-study bias","Imprecision","Incoherence"] |
| B:D | 0 | Some concerns | Low risk | No concerns | Major concerns | No concerns | Some concerns | Very low | ["Within-study bias","Imprecision","Incoherence"] |
| B:E | 0 | Some concerns | Low risk | No concerns | Major concerns | No concerns | Some concerns | Very low | ["Within-study bias","Imprecision","Incoherence"] |
| B:F | 0 | Some concerns | Low risk | No concerns | Major concerns | No concerns | Some concerns | Very low | ["Within-study bias","Imprecision","Incoherence"] |
| B:G | 0 | Some concerns | Low risk | No concerns | Major concerns | No concerns | Some concerns | Very low | ["Within-study bias","Imprecision","Incoherence"] |
| B:H | 0 | Some concerns | Low risk | No concerns | No concerns | Major concerns | Some concerns | Very low | ["Within-study bias","Heterogeneity","Incoherence"] |
| B:I | 0 | No concerns | Low risk | No concerns | Major concerns | No concerns | Some concerns | Low | ["Imprecision","Incoherence"] |
| B:J | 0 | Some concerns | Low risk | No concerns | Major concerns | No concerns | Some concerns | Very low | ["Within-study bias","Imprecision","Incoherence"] |
| C:D | 0 | Some concerns | Low risk | No concerns | Major concerns | No concerns | Some concerns | Very low | ["Within-study bias","Imprecision","Incoherence"] |
| C:E | 0 | No concerns | Low risk | No concerns | No concerns | Major concerns | Some concerns | Low | ["Heterogeneity","Incoherence"] |
| C:F | 0 | No concerns | Low risk | No concerns | No concerns | Major concerns | Some concerns | Low | ["Heterogeneity","Incoherence"] |
| C:G | 0 | Some concerns | Low risk | No concerns | Major concerns | No concerns | Some concerns | Very low | ["Within-study bias","Imprecision","Incoherence"] |
| C:H | 0 | No concerns | Low risk | No concerns | No concerns | Major concerns | Some concerns | Low | ["Heterogeneity","Incoherence"] |
| C:I | 0 | No concerns | Low risk | No concerns | Major concerns | No concerns | Some concerns | Low | ["Imprecision","Incoherence"] |
| C:J | 0 | Some concerns | Low risk | No concerns | Major concerns | No concerns | Some concerns | Very low | ["Within-study bias","Imprecision","Incoherence"] |
| D:E | 0 | No concerns | Low risk | No concerns | Major concerns | No concerns | Some concerns | Low | ["Imprecision","Incoherence"] |
| D:F | 0 | No concerns | Low risk | No concerns | Major concerns | No concerns | Some concerns | Low | ["Imprecision","Incoherence"] |
| D:G | 0 | Some concerns | Low risk | No concerns | Major concerns | No concerns | Some concerns | Very low | ["Within-study bias","Imprecision","Incoherence"] |
| D:H | 0 | No concerns | Low risk | No concerns | No concerns | Major concerns | Some concerns | Low | ["Heterogeneity","Incoherence"] |
| D:I | 0 | No concerns | Low risk | No concerns | Major concerns | No concerns | Some concerns | Low | ["Imprecision","Incoherence"] |
| D:J | 0 | Some concerns | Low risk | No concerns | Major concerns | No concerns | Some concerns | Very low | ["Within-study bias","Imprecision","Incoherence"] |
| E:F | 0 | No concerns | Low risk | No concerns | Major concerns | No concerns | Some concerns | Low | ["Imprecision","Incoherence"] |
| E:G | 0 | Some concerns | Low risk | No concerns | Major concerns | No concerns | Some concerns | Very low | ["Within-study bias","Imprecision","Incoherence"] |
| E:H | 0 | No concerns | Low risk | No concerns | Major concerns | No concerns | Some concerns | Low | ["Imprecision","Incoherence"] |
| E:I | 0 | No concerns | Low risk | No concerns | Major concerns | No concerns | Some concerns | Low | ["Imprecision","Incoherence"] |
| E:J | 0 | Some concerns | Low risk | No concerns | Major concerns | No concerns | Some concerns | Very low | ["Within-study bias","Imprecision","Incoherence"] |
| F:G | 0 | Some concerns | Low risk | No concerns | Major concerns | No concerns | Some concerns | Very low | ["Within-study bias","Imprecision","Incoherence"] |
| F:H | 0 | No concerns | Low risk | No concerns | Major concerns | No concerns | Some concerns | Low | ["Imprecision","Incoherence"] |
| F:I | 0 | No concerns | Low risk | No concerns | Major concerns | No concerns | Some concerns | Low | ["Imprecision","Incoherence"] |
| F:J | 0 | Some concerns | Low risk | No concerns | Major concerns | No concerns | Some concerns | Very low | ["Within-study bias","Imprecision","Incoherence"] |
| G:H | 0 | Some concerns | Low risk | No concerns | No concerns | Major concerns | Some concerns | Very low | ["Within-study bias","Heterogeneity","Incoherence"] |
| G:I | 0 | Some concerns | Low risk | No concerns | Major concerns | No concerns | Some concerns | Very low | ["Within-study bias","Imprecision","Incoherence"] |
| G:J | 0 | Some concerns | Low risk | No concerns | Major concerns | No concerns | Some concerns | Very low | ["Within-study bias","Imprecision","Incoherence"] |
| H:I | 0 | No concerns | Low risk | No concerns | Major concerns | No concerns | Some concerns | Low | ["Imprecision","Incoherence"] |
| H:J | 0 | Some concerns | Low risk | No concerns | Major concerns | No concerns | Some concerns | Very low | ["Within-study bias","Imprecision","Incoherence"] |
| I:J | 0 | Some concerns | Low risk | No concerns | Major concerns | No concerns | Some concerns | Very low | ["Within-study bias","Imprecision","Incoherence"] |

Table A.19: GRADE assessment: BUN

| Comparison | Number of studies | Within-study bias | Reporting bias | Indirectness | Imprecision | Heterogeneity | Incoherence | Confidence rating | Reason(s) for downgrading |
| --- | --- | --- | --- | --- | --- | --- | --- | --- | --- |
| A:B | 22 | Some concerns | Low risk | No concerns | No concerns | Major concerns | Some concerns | Very low | ["Within-study bias","Heterogeneity","Incoherence"] |
| A:C | 10 | Some concerns | Low risk | No concerns | Major concerns | No concerns | Some concerns | Very low | ["Within-study bias","Imprecision","Incoherence"] |
| A:D | 3 | Some concerns | Low risk | No concerns | No concerns | Major concerns | Some concerns | Very low | ["Within-study bias","Heterogeneity","Incoherence"] |
| A:E | 6 | No concerns | Low risk | No concerns | Major concerns | No concerns | Some concerns | Low | ["Imprecision","Incoherence"] |
| A:F | 2 | Some concerns | Low risk | No concerns | No concerns | No concerns | Some concerns | Low | ["Within-study bias","Incoherence"] |
| A:G | 5 | Some concerns | Low risk | No concerns | Major concerns | No concerns | Some concerns | Very low | ["Within-study bias","Imprecision","Incoherence"] |
| A:H | 6 | No concerns | Low risk | No concerns | No concerns | Major concerns | Some concerns | Low | ["Heterogeneity","Incoherence"] |
| A:I | 3 | No concerns | Low risk | No concerns | Major concerns | No concerns | Some concerns | Low | ["Imprecision","Incoherence"] |
| A:J | 2 | Some concerns | Low risk | No concerns | Major concerns | No concerns | Some concerns | Very low | ["Within-study bias","Imprecision","Incoherence"] |
| A:K | 3 | Some concerns | Low risk | No concerns | Major concerns | No concerns | Some concerns | Very low | ["Within-study bias","Imprecision","Incoherence"] |
| B:C | 0 | Some concerns | Low risk | No concerns | Major concerns | No concerns | Some concerns | Very low | ["Within-study bias","Imprecision","Incoherence"] |
| B:D | 0 | Some concerns | Low risk | No concerns | Major concerns | No concerns | Some concerns | Very low | ["Within-study bias","Imprecision","Incoherence"] |
| B:E | 0 | Some concerns | Low risk | No concerns | Major concerns | No concerns | Some concerns | Very low | ["Within-study bias","Imprecision","Incoherence"] |
| B:F | 0 | Some concerns | Low risk | No concerns | No concerns | Major concerns | Some concerns | Very low | ["Within-study bias","Heterogeneity","Incoherence"] |
| B:G | 0 | Some concerns | Low risk | No concerns | Major concerns | No concerns | Some concerns | Very low | ["Within-study bias","Imprecision","Incoherence"] |
| B:H | 0 | Some concerns | Low risk | No concerns | No concerns | Major concerns | Some concerns | Very low | ["Heterogeneity","Incoherence"] |
| B:I | 0 | No concerns | Low risk | No concerns | Major concerns | No concerns | Some concerns | Low | ["Imprecision","Incoherence"] |
| B:J | 0 | Some concerns | Low risk | No concerns | Major concerns | No concerns | Some concerns | Very low | ["Within-study bias","Imprecision","Incoherence"] |
| B:K | 0 | Some concerns | Low risk | No concerns | Major concerns | No concerns | Some concerns | Very low | ["Within-study bias","Imprecision","Incoherence"] |
| C:D | 0 | Some concerns | Low risk | No concerns | Major concerns | No concerns | Some concerns | Very low | ["Within-study bias","Imprecision","Incoherence"] |
| C:E | 0 | No concerns | Low risk | No concerns | Major concerns | No concerns | Some concerns | Low | ["Imprecision","Incoherence"] |
| C:F | 0 | Some concerns | Low risk | No concerns | No concerns | Major concerns | Some concerns | Very low | ["Within-study bias","Heterogeneity","Incoherence"] |
| C:G | 0 | Some concerns | Low risk | No concerns | Major concerns | No concerns | Some concerns | Very low | ["Within-study bias","Imprecision","Incoherence"] |
| C:H | 0 | Some concerns | Low risk | No concerns | No concerns | Major concerns | Some concerns | Very low | ["Within-study bias","Heterogeneity","Incoherence"] |
| C:I | 0 | No concerns | Low risk | No concerns | Major concerns | No concerns | Some concerns | Low | ["Imprecision","Incoherence"] |
| C:J | 0 | Some concerns | Low risk | No concerns | Major concerns | No concerns | Some concerns | Very low | ["Within-study bias","Imprecision","Incoherence"] |
| C:K | 0 | Some concerns | Low risk | No concerns | Major concerns | No concerns | Some concerns | Very low | ["Within-study bias","Imprecision","Incoherence"] |
| D:E | 0 | No concerns | Low risk | No concerns | Major concerns | No concerns | Some concerns | Low | ["Imprecision","Incoherence"] |
| D:F | 0 | Some concerns | Low risk | No concerns | Major concerns | No concerns | Some concerns | Very low | ["Within-study bias","Imprecision","Incoherence"] |
| D:G | 0 | Some concerns | Low risk | No concerns | Major concerns | No concerns | Some concerns | Very low | ["Within-study bias","Imprecision","Incoherence"] |
| D:H | 0 | Some concerns | Low risk | No concerns | Major concerns | No concerns | Some concerns | Very low | ["Within-study bias","Imprecision","Incoherence"] |
| D:I | 0 | No concerns | Low risk | No concerns | Major concerns | No concerns | Some concerns | Low | ["Imprecision","Incoherence"] |
| D:J | 0 | Some concerns | Low risk | No concerns | Major concerns | No concerns | Some concerns | Very low | ["Within-study bias","Imprecision","Incoherence"] |
| D:K | 0 | Some concerns | Low risk | No concerns | Major concerns | No concerns | Some concerns | Very low | ["Within-study bias","Imprecision","Incoherence"] |
| E:F | 0 | No concerns | Low risk | No concerns | No concerns | Major concerns | Some concerns | Low | ["Heterogeneity","Incoherence"] |
| E:G | 0 | Some concerns | Low risk | No concerns | Major concerns | No concerns | Some concerns | Very low | ["Within-study bias","Imprecision","Incoherence"] |
| E:H | 0 | No concerns | Low risk | No concerns | No concerns | Major concerns | Some concerns | Low | ["Heterogeneity","Incoherence"] |
| E:I | 0 | No concerns | Low risk | No concerns | Major concerns | No concerns | Some concerns | Low | ["Imprecision","Incoherence"] |
| E:J | 0 | Some concerns | Low risk | No concerns | Major concerns | No concerns | Some concerns | Very low | ["Within-study bias","Imprecision","Incoherence"] |
| E:K | 0 | Some concerns | Low risk | No concerns | Major concerns | No concerns | Some concerns | Very low | ["Within-study bias","Imprecision","Incoherence"] |
| F:G | 0 | Some concerns | Low risk | No concerns | No concerns | Major concerns | Some concerns | Very low | ["Within-study bias","Heterogeneity","Incoherence"] |
| F:H | 0 | No concerns | Low risk | No concerns | Major concerns | No concerns | Some concerns | Low | ["Imprecision","Incoherence"] |
| F:I | 0 | No concerns | Low risk | No concerns | No concerns | Major concerns | Some concerns | Low | ["Heterogeneity","Incoherence"] |
| F:J | 0 | Some concerns | Low risk | No concerns | Major concerns | No concerns | Some concerns | Very low | ["Within-study bias","Imprecision","Incoherence"] |
| F:K | 0 | Some concerns | Low risk | No concerns | Major concerns | No concerns | Some concerns | Very low | ["Within-study bias","Imprecision","Incoherence"] |
| G:H | 0 | Some concerns | Low risk | No concerns | Major concerns | No concerns | Some concerns | Very low | ["Within-study bias","Imprecision","Incoherence"] |
| G:I | 0 | No concerns | Low risk | No concerns | Major concerns | No concerns | Some concerns | Low | ["Imprecision","Incoherence"] |
| G:J | 0 | Some concerns | Low risk | No concerns | Major concerns | No concerns | Some concerns | Very low | ["Within-study bias","Imprecision","Incoherence"] |
| G:K | 0 | Some concerns | Low risk | No concerns | Major concerns | No concerns | Some concerns | Very low | ["Within-study bias","Imprecision","Incoherence"] |
| H:I | 0 | No concerns | Low risk | No concerns | Major concerns | No concerns | Some concerns | Low | ["Imprecision","Incoherence"] |
| H:J | 0 | Some concerns | Low risk | No concerns | Major concerns | No concerns | Some concerns | Very low | ["Within-study bias","Imprecision","Incoherence"] |
| H:K | 0 | Some concerns | Low risk | No concerns | Major concerns | No concerns | Some concerns | Very low | ["Within-study bias","Imprecision","Incoherence"] |
| I:J | 0 | Some concerns | Low risk | No concerns | Major concerns | No concerns | Some concerns | Very low | ["Within-study bias","Imprecision","Incoherence"] |
| I:K | 0 | Some concerns | Low risk | No concerns | Major concerns | No concerns | Some concerns | Very low | ["Within-study bias","Imprecision","Incoherence"] |
| J:K | 0 | Some concerns | Low risk | No concerns | Major concerns | No concerns | Some concerns | Very low | ["Within-study bias","Imprecision","Incoherence"] |

Table A.20: GRADE assessment: β_2_-MG

| Comparison | Number of studies | Within-study bias | Reporting bias | Indirectness | Imprecision | Heterogeneity | Incoherence | Confidence rating | Reason(s) for downgrading |
| --- | --- | --- | --- | --- | --- | --- | --- | --- | --- |
| A:B | 10 | Some concerns | Low risk | No concerns | No concerns | Major concerns | Some concerns | Very low | ["Within-study bias","Heterogeneity","Incoherence"] |
| A:C | 3 | Some concerns | Low risk | No concerns | No concerns | Major concerns | Some concerns | Very low | ["Within-study bias","Heterogeneity","Incoherence"] |
| A:D | 1 | No concerns | Low risk | No concerns | Major concerns | No concerns | Some concerns | Low | ["Imprecision","Incoherence"] |
| A:E | 2 | Some concerns | Low risk | No concerns | Major concerns | No concerns | Some concerns | Very low | ["Within-study bias","Imprecision","Incoherence"] |
| A:F | 1 | Some concerns | Low risk | No concerns | Major concerns | No concerns | Some concerns | Very low | ["Within-study bias","Imprecision","Incoherence"] |
| A:G | 3 | Some concerns | Low risk | No concerns | No concerns | Major concerns | Some concerns | Very low | ["Within-study bias","Heterogeneity","Incoherence"] |
| A:H | 4 | No concerns | Low risk | No concerns | No concerns | Major concerns | Some concerns | Low | ["Heterogeneity","Incoherence"] |
| A:I | 3 | No concerns | Low risk | No concerns | No concerns | Major concerns | Some concerns | Low | ["Heterogeneity","Incoherence"] |
| A:J | 1 | Some concerns | Low risk | No concerns | Major concerns | No concerns | Some concerns | Very low | ["Within-study bias","Imprecision","Incoherence"] |
| B:C | 0 | Some concerns | Low risk | No concerns | Major concerns | No concerns | Some concerns | Very low | ["Within-study bias","Imprecision","Incoherence"] |
| B:D | 0 | No concerns | Low risk | No concerns | Major concerns | No concerns | Some concerns | Low | ["Imprecision","Incoherence"] |
| B:E | 0 | Some concerns | Low risk | No concerns | Major concerns | No concerns | Some concerns | Very low | ["Within-study bias","Imprecision","Incoherence"] |
| B:F | 0 | Some concerns | Low risk | No concerns | Major concerns | No concerns | Some concerns | Very low | ["Within-study bias","Imprecision","Incoherence"] |
| B:G | 0 | Some concerns | Low risk | No concerns | Major concerns | No concerns | Some concerns | Very low | ["Within-study bias","Imprecision","Incoherence"] |
| B:H | 0 | No concerns | Low risk | No concerns | Major concerns | No concerns | Some concerns | Low | ["Imprecision","Incoherence"] |
| B:I | 0 | No concerns | Low risk | No concerns | Major concerns | No concerns | Some concerns | Low | ["Incoherence"] |
| B:J | 0 | Some concerns | Low risk | No concerns | Major concerns | No concerns | Some concerns | Very low | ["Within-study bias","Imprecision","Incoherence"] |
| C:D | 0 | Some concerns | Low risk | No concerns | Major concerns | No concerns | Some concerns | Very low | ["Within-study bias","Imprecision","Incoherence"] |
| C:E | 0 | Some concerns | Low risk | No concerns | Major concerns | No concerns | Some concerns | Very low | ["Within-study bias","Imprecision","Incoherence"] |
| C:F | 0 | Some concerns | Low risk | No concerns | Major concerns | No concerns | Some concerns | Very low | ["Within-study bias","Imprecision","Incoherence"] |
| C:G | 0 | Some concerns | Low risk | No concerns | Major concerns | No concerns | Some concerns | Very low | ["Within-study bias","Imprecision","Incoherence"] |
| C:H | 0 | Some concerns | Low risk | No concerns | Major concerns | No concerns | Some concerns | Very low | ["Within-study bias","Imprecision","Incoherence"] |
| C:I | 0 | Some concerns | Low risk | No concerns | Major concerns | No concerns | Some concerns | Very low | ["Within-study bias","Imprecision","Incoherence"] |
| C:J | 0 | Some concerns | Low risk | No concerns | Major concerns | No concerns | Some concerns | Very low | ["Within-study bias","Imprecision","Incoherence"] |
| D:E | 0 | Some concerns | Low risk | No concerns | Major concerns | No concerns | Some concerns | Very low | ["Within-study bias","Imprecision","Incoherence"] |
| D:F | 0 | Some concerns | Low risk | No concerns | Major concerns | No concerns | Some concerns | Very low | ["Within-study bias","Imprecision","Incoherence"] |
| D:G | 0 | Some concerns | Low risk | No concerns | Major concerns | No concerns | Some concerns | Very low | ["Within-study bias","Imprecision","Incoherence"] |
| D:H | 0 | No concerns | Low risk | No concerns | Major concerns | No concerns | Some concerns | Low | ["Imprecision","Incoherence"] |
| D:I | 0 | No concerns | Low risk | No concerns | Major concerns | No concerns | Some concerns | Low | ["Imprecision","Incoherence"] |
| D:J | 0 | Some concerns | Low risk | No concerns | Major concerns | No concerns | Some concerns | Very low | ["Within-study bias","Imprecision","Incoherence"] |
| E:F | 0 | Some concerns | Low risk | No concerns | Major concerns | No concerns | Some concerns | Very low | ["Within-study bias","Imprecision","Incoherence"] |
| E:G | 0 | Some concerns | Low risk | No concerns | Major concerns | No concerns | Some concerns | Very low | ["Within-study bias","Imprecision","Incoherence"] |
| E:H | 0 | Some concerns | Low risk | No concerns | Major concerns | No concerns | Some concerns | Very low | ["Within-study bias","Imprecision","Incoherence"] |
| E:I | 0 | Some concerns | Low risk | No concerns | Major concerns | No concerns | Some concerns | Very low | ["Within-study bias","Imprecision","Incoherence"] |
| E:J | 0 | Some concerns | Low risk | No concerns | Major concerns | No concerns | Some concerns | Very low | ["Within-study bias","Imprecision","Incoherence"] |
| F:G | 0 | Some concerns | Low risk | No concerns | Major concerns | No concerns | Some concerns | Very low | ["Within-study bias","Imprecision","Incoherence"] |
| F:H | 0 | Some concerns | Low risk | No concerns | Major concerns | No concerns | Some concerns | Very low | ["Within-study bias","Imprecision","Incoherence"] |
| F:I | 0 | Some concerns | Low risk | No concerns | Major concerns | No concerns | Some concerns | Very low | ["Within-study bias","Imprecision","Incoherence"] |
| F:J | 0 | Some concerns | Low risk | No concerns | Major concerns | No concerns | Some concerns | Very low | ["Within-study bias","Imprecision","Incoherence"] |
| G:H | 0 | Some concerns | Low risk | No concerns | Major concerns | No concerns | Some concerns | Very low | ["Within-study bias","Imprecision","Incoherence"] |
| G:I | 0 | Some concerns | Low risk | No concerns | Major concerns | No concerns | Some concerns | Very low | ["Within-study bias","Imprecision","Incoherence"] |
| G:J | 0 | Some concerns | Low risk | No concerns | Major concerns | No concerns | Some concerns | Very low | ["Within-study bias","Imprecision","Incoherence"] |
| H:I | 0 | No concerns | Low risk | No concerns | Major concerns | No concerns | Some concerns | Low | ["Imprecision","Incoherence"] |
| H:J | 0 | Some concerns | Low risk | No concerns | Major concerns | No concerns | Some concerns | Very low | ["Within-study bias","Imprecision","Incoherence"] |
| I:J | 0 | Some concerns | Low risk | No concerns | Major concerns | No concerns | Some concerns | Very low | ["Within-study bias","Imprecision","Incoherence"] |

Table A.21: GRADE assessment: TC

| Comparison | Number of studies | Within-study bias | Reporting bias | Indirectness | Imprecision | Heterogeneity | Incoherence | Confidence rating | Reason(s) for downgrading |
| --- | --- | --- | --- | --- | --- | --- | --- | --- | --- |
| A:B | 6 | Some concerns | Low risk | No concerns | No concerns | Major concerns | Some concerns | Very low | ["Within-study bias","Heterogeneity","Incoherence"] |
| A:C | 4 | Some concerns | Low risk | No concerns | No concerns | No concerns | Some concerns | Very low | ["Within-study bias","Incoherence"] |
| A:D | 1 | Some concerns | Low risk | No concerns | Major concerns | No concerns | Some concerns | Very low | ["Within-study bias","Imprecision","Incoherence"] |
| A:E | 2 | No concerns | Low risk | No concerns | No concerns | Major concerns | Some concerns | Low | ["Heterogeneity","Incoherence"] |
| A:F | 1 | No concerns | Low risk | No concerns | Major concerns | No concerns | Some concerns | Low | ["Imprecision","Incoherence"] |
| A:G | 1 | Some concerns | Low risk | No concerns | Major concerns | No concerns | Some concerns | Very low | ["Within-study bias","Imprecision","Incoherence"] |
| A:H | 4 | Some concerns | Low risk | No concerns | Major concerns | No concerns | Some concerns | Very low | ["Within-study bias","Imprecision","Incoherence"] |
| A:I | 1 | Some concerns | Low risk | No concerns | No concerns | No concerns | Some concerns | Very low | ["Within-study bias","Incoherence"] |
| A:J | 1 | Some concerns | Low risk | No concerns | No concerns | Major concerns | Some concerns | Very low | ["Within-study bias","Heterogeneity","Incoherence"] |
| B:C | 0 | Some concerns | Low risk | No concerns | Major concerns | No concerns | Some concerns | Very low | ["Within-study bias","Imprecision","Incoherence"] |
| B:D | 0 | Some concerns | Low risk | No concerns | Major concerns | No concerns | Some concerns | Very low | ["Within-study bias","Imprecision","Incoherence"] |
| B:E | 0 | No concerns | Low risk | No concerns | Major concerns | No concerns | Some concerns | Low | ["Imprecision","Incoherence"] |
| B:F | 0 | No concerns | Low risk | No concerns | Major concerns | No concerns | Some concerns | Low | ["Imprecision","Incoherence"] |
| B:G | 0 | Some concerns | Low risk | No concerns | Major concerns | No concerns | Some concerns | Very low | ["Within-study bias","Imprecision","Incoherence"] |
| B:H | 0 | Some concerns | Low risk | No concerns | Major concerns | No concerns | Some concerns | Very low | ["Within-study bias","Imprecision","Incoherence"] |
| B:I | 0 | Some concerns | Low risk | No concerns | Major concerns | No concerns | Some concerns | Very low | ["Within-study bias","Imprecision","Incoherence"] |
| B:J | 0 | Some concerns | Low risk | No concerns | Major concerns | No concerns | Some concerns | Very low | ["Within-study bias","Imprecision","Incoherence"] |
| C:D | 0 | Some concerns | Low risk | No concerns | Major concerns | No concerns | Some concerns | Very low | ["Within-study bias","Imprecision","Incoherence"] |
| C:E | 0 | Some concerns | Low risk | No concerns | Major concerns | No concerns | Some concerns | Very low | ["Within-study bias","Imprecision","Incoherence"] |
| C:F | 0 | No concerns | Low risk | No concerns | Major concerns | No concerns | Some concerns | Low | ["Imprecision","Incoherence"] |
| C:G | 0 | Some concerns | Low risk | No concerns | Major concerns | No concerns | Some concerns | Very low | ["Within-study bias","Imprecision","Incoherence"] |
| C:H | 0 | Some concerns | Low risk | No concerns | No concerns | Major concerns | Some concerns | Very low | ["Within-study bias","Heterogeneity","Incoherence"] |
| C:I | 0 | Some concerns | Low risk | No concerns | Major concerns | No concerns | Some concerns | Very low | ["Within-study bias","Imprecision","Incoherence"] |
| C:J | 0 | Some concerns | Low risk | No concerns | Major concerns | No concerns | Some concerns | Very low | ["Within-study bias","Imprecision","Incoherence"] |
| D:E | 0 | Some concerns | Low risk | No concerns | Major concerns | No concerns | Some concerns | Very low | ["Within-study bias","Imprecision","Incoherence"] |
| D:F | 0 | Some concerns | Low risk | No concerns | Major concerns | No concerns | Some concerns | Very low | ["Within-study bias","Imprecision","Incoherence"] |
| D:G | 0 | Some concerns | Low risk | No concerns | Major concerns | No concerns | Some concerns | Very low | ["Within-study bias","Imprecision","Incoherence"] |
| D:H | 0 | Some concerns | Low risk | No concerns | Major concerns | No concerns | Some concerns | Very low | ["Within-study bias","Imprecision","Incoherence"] |
| D:I | 0 | Some concerns | Low risk | No concerns | Major concerns | No concerns | Some concerns | Very low | ["Within-study bias","Imprecision","Incoherence"] |
| D:J | 0 | Some concerns | Low risk | No concerns | Major concerns | No concerns | Some concerns | Very low | ["Within-study bias","Imprecision","Incoherence"] |
| E:F | 0 | No concerns | Low risk | No concerns | Major concerns | No concerns | Some concerns | Low | ["Imprecision","Incoherence"] |
| E:G | 0 | Some concerns | Low risk | No concerns | Major concerns | No concerns | Some concerns | Very low | ["Within-study bias","Imprecision","Incoherence"] |
| E:H | 0 | Some concerns | Low risk | No concerns | Major concerns | No concerns | Some concerns | Very low | ["Within-study bias","Imprecision","Incoherence"] |
| E:I | 0 | Some concerns | Low risk | No concerns | Major concerns | No concerns | Some concerns | Very low | ["Within-study bias","Imprecision","Incoherence"] |
| E:J | 0 | Some concerns | Low risk | No concerns | Major concerns | No concerns | Some concerns | Very low | ["Within-study bias","Imprecision","Incoherence"] |
| F:G | 0 | Some concerns | Low risk | No concerns | Major concerns | No concerns | Some concerns | Very low | ["Within-study bias","Imprecision","Incoherence"] |
| F:H | 0 | No concerns | Low risk | No concerns | Major concerns | No concerns | Some concerns | Low | ["Imprecision","Incoherence"] |
| F:I | 0 | Some concerns | Low risk | No concerns | Major concerns | No concerns | Some concerns | Very low | ["Within-study bias","Imprecision","Incoherence"] |
| F:J | 0 | Some concerns | Low risk | No concerns | Major concerns | No concerns | Some concerns | Very low | ["Within-study bias","Imprecision","Incoherence"] |
| G:H | 0 | Some concerns | Low risk | No concerns | Major concerns | No concerns | Some concerns | Very low | ["Within-study bias","Imprecision","Incoherence"] |
| G:I | 0 | Some concerns | Low risk | No concerns | Major concerns | No concerns | Some concerns | Very low | ["Within-study bias","Imprecision","Incoherence"] |
| G:J | 0 | Some concerns | Low risk | No concerns | Major concerns | No concerns | Some concerns | Very low | ["Within-study bias","Imprecision","Incoherence"] |
| H:I | 0 | Some concerns | Low risk | No concerns | No concerns | Major concerns | Some concerns | Very low | ["Within-study bias","Heterogeneity","Incoherence"] |
| H:J | 0 | Some concerns | Low risk | No concerns | Major concerns | No concerns | Some concerns | Very low | ["Within-study bias","Imprecision","Incoherence"] |
| I:J | 0 | Some concerns | Low risk | No concerns | Major concerns | No concerns | Some concerns | Very low | ["Within-study bias","Imprecision","Incoherence"] |

Table A.22: GRADE assessment: TG

| Comparison | Number of studies | Within-study bias | Reporting bias | Indirectness | Imprecision | Heterogeneity | Incoherence | Confidence rating | Reason(s) for downgrading |
| --- | --- | --- | --- | --- | --- | --- | --- | --- | --- |
| A:B | 7 | Some concerns | Low risk | No concerns | No concerns | No concerns | Some concerns | Low | ["Within-study bias","Incoherence"] |
| A:C | 4 | Some concerns | Low risk | No concerns | No concerns | Major concerns | Some concerns | Very low | ["Within-study bias","Heterogeneity","Incoherence"] |
| A:D | 1 | Some concerns | Low risk | No concerns | Major concerns | No concerns | Some concerns | Very low | ["Within-study bias","Imprecision","Incoherence"] |
| A:E | 3 | No concerns | Low risk | No concerns | No concerns | No concerns | Some concerns | Moderate | ["Incoherence"] |
| A:F | 1 | No concerns | Low risk | No concerns | Major concerns | No concerns | Some concerns | Low | ["Imprecision","Incoherence"] |
| A:G | 2 | Some concerns | Low risk | No concerns | Major concerns | No concerns | Some concerns | Very low | ["Within-study bias","Imprecision","Incoherence"] |
| A:H | 4 | Some concerns | Low risk | No concerns | Major concerns | No concerns | Some concerns | Very low | ["Within-study bias","Imprecision","Incoherence"] |
| A:I | 1 | Some concerns | Low risk | No concerns | Major concerns | No concerns | Some concerns | Very low | ["Within-study bias","Imprecision","Incoherence"] |
| A:J | 1 | Some concerns | Low risk | No concerns | Major concerns | No concerns | Some concerns | Very low | ["Within-study bias","Imprecision","Incoherence"] |
| B:C | 0 | Some concerns | Low risk | No concerns | Major concerns | No concerns | Some concerns | Very low | ["Within-study bias","Imprecision","Incoherence"] |
| B:D | 0 | Some concerns | Low risk | No concerns | Major concerns | No concerns | Some concerns | Very low | ["Within-study bias","Imprecision","Incoherence"] |
| B:E | 0 | No concerns | Low risk | No concerns | Major concerns | No concerns | Some concerns | Low | ["Imprecision","Incoherence"] |
| B:F | 0 | No concerns | Low risk | No concerns | Major concerns | No concerns | Some concerns | Low | ["Imprecision","Incoherence"] |
| B:G | 0 | Some concerns | Low risk | No concerns | No concerns | Major concerns | Some concerns | Very low | ["Within-study bias","Heterogeneity","Incoherence"] |
| B:H | 0 | Some concerns | Low risk | No concerns | No concerns | Major concerns | Some concerns | Very low | ["Within-study bias","Heterogeneity","Incoherence"] |
| B:I | 0 | Some concerns | Low risk | No concerns | Major concerns | No concerns | Some concerns | Very low | ["Within-study bias","Imprecision","Incoherence"] |
| B:J | 0 | Some concerns | Low risk | No concerns | Major concerns | No concerns | Some concerns | Very low | ["Within-study bias","Imprecision","Incoherence"] |
| C:D | 0 | Some concerns | Low risk | No concerns | Major concerns | No concerns | Some concerns | Very low | ["Within-study bias","Imprecision","Incoherence"] |
| C:E | 0 | Some concerns | Low risk | No concerns | Major concerns | No concerns | Some concerns | Very low | ["Within-study bias","Imprecision","Incoherence"] |
| C:F | 0 | Some concerns | Low risk | No concerns | Major concerns | No concerns | Some concerns | Very low | ["Within-study bias","Imprecision","Incoherence"] |
| C:G | 0 | Some concerns | Low risk | No concerns | Major concerns | No concerns | Some concerns | Very low | ["Within-study bias","Imprecision","Incoherence"] |
| C:H | 0 | Some concerns | Low risk | No concerns | Major concerns | No concerns | Some concerns | Very low | ["Within-study bias","Imprecision","Incoherence"] |
| C:I | 0 | Some concerns | Low risk | No concerns | Major concerns | No concerns | Some concerns | Very low | ["Within-study bias","Imprecision","Incoherence"] |
| C:J | 0 | Some concerns | Low risk | No concerns | Major concerns | No concerns | Some concerns | Very low | ["Within-study bias","Imprecision","Incoherence"] |
| D:E | 0 | Some concerns | Low risk | No concerns | Major concerns | No concerns | Some concerns | Very low | ["Within-study bias","Imprecision","Incoherence"] |
| D:F | 0 | Some concerns | Low risk | No concerns | Major concerns | No concerns | Some concerns | Very low | ["Within-study bias","Imprecision","Incoherence"] |
| D:G | 0 | Some concerns | Low risk | No concerns | Major concerns | No concerns | Some concerns | Very low | ["Within-study bias","Imprecision","Incoherence"] |
| D:H | 0 | Some concerns | Low risk | No concerns | Major concerns | No concerns | Some concerns | Very low | ["Within-study bias","Imprecision","Incoherence"] |
| D:I | 0 | Some concerns | Low risk | No concerns | Major concerns | No concerns | Some concerns | Very low | ["Within-study bias","Imprecision","Incoherence"] |
| D:J | 0 | Some concerns | Low risk | No concerns | Major concerns | No concerns | Some concerns | Very low | ["Within-study bias","Imprecision","Incoherence"] |
| E:F | 0 | No concerns | Low risk | No concerns | Major concerns | No concerns | Some concerns | Low | ["Imprecision","Incoherence"] |
| E:G | 0 | Some concerns | Low risk | No concerns | No concerns | Major concerns | Some concerns | Very low | ["Within-study bias","Heterogeneity","Incoherence"] |
| E:H | 0 | Some concerns | Low risk | No concerns | No concerns | Major concerns | Some concerns | Very low | ["Within-study bias","Heterogeneity","Incoherence"] |
| E:I | 0 | Some concerns | Low risk | No concerns | Major concerns | No concerns | Some concerns | Very low | ["Within-study bias","Imprecision","Incoherence"] |
| E:J | 0 | Some concerns | Low risk | No concerns | Major concerns | No concerns | Some concerns | Very low | ["Within-study bias","Imprecision","Incoherence"] |
| F:G | 0 | Some concerns | Low risk | No concerns | Major concerns | No concerns | Some concerns | Very low | ["Within-study bias","Imprecision","Incoherence"] |
| F:H | 0 | No concerns | Low risk | No concerns | Major concerns | No concerns | Some concerns | Low | ["Imprecision","Incoherence"] |
| F:I | 0 | Some concerns | Low risk | No concerns | Major concerns | No concerns | Some concerns | Very low | ["Within-study bias","Imprecision","Incoherence"] |
| F:J | 0 | Some concerns | Low risk | No concerns | Major concerns | No concerns | Some concerns | Very low | ["Within-study bias","Imprecision","Incoherence"] |
| G:H | 0 | Some concerns | Low risk | No concerns | Major concerns | No concerns | Some concerns | Very low | ["Within-study bias","Imprecision","Incoherence"] |
| G:I | 0 | Some concerns | Low risk | No concerns | Major concerns | No concerns | Some concerns | Very low | ["Within-study bias","Imprecision","Incoherence"] |
| G:J | 0 | Some concerns | Low risk | No concerns | Major concerns | No concerns | Some concerns | Very low | ["Within-study bias","Imprecision","Incoherence"] |
| H:I | 0 | Some concerns | Low risk | No concerns | Major concerns | No concerns | Some concerns | Very low | ["Within-study bias","Imprecision","Incoherence"] |
| H:J | 0 | Some concerns | Low risk | No concerns | Major concerns | No concerns | Some concerns | Very low | ["Within-study bias","Imprecision","Incoherence"] |
| I:J | 0 | Some concerns | Low risk | No concerns | Major concerns | No concerns | Some concerns | Very low | ["Within-study bias","Imprecision","Incoherence"] |

Table A.23: GRADE assessment: SBP

| Comparison | Number of studies | Within-study bias | Reporting bias | Indirectness | Imprecision | Heterogeneity | Incoherence | Confidence rating | Reason(s) for downgrading |
| --- | --- | --- | --- | --- | --- | --- | --- | --- | --- |
| A:B | 9 | Some concerns | Low risk | No concerns | Major concerns | No concerns | Some concerns | Very low | ["Within-study bias","Imprecision","Incoherence"] |
| A:C | 3 | Some concerns | Low risk | No concerns | Major concerns | No concerns | Some concerns | Very low | ["Within-study bias","Imprecision","Incoherence"] |
| A:D | 1 | Some concerns | Low risk | No concerns | No concerns | No concerns | Some concerns | Low | ["Within-study bias","Incoherence"] |
| A:E | 2 | No concerns | Low risk | No concerns | Major concerns | No concerns | Some concerns | Very low | ["Imprecision","Incoherence"] |
| A:F | 2 | Some concerns | Low risk | No concerns | Major concerns | No concerns | Some concerns | Very low | ["Within-study bias","Imprecision","Incoherence"] |
| A:G | 1 | Some concerns | Low risk | No concerns | Major concerns | No concerns | Some concerns | Very low | ["Within-study bias","Imprecision","Incoherence"] |
| B:C | 0 | Some concerns | Low risk | No concerns | Major concerns | No concerns | Some concerns | Very low | ["Within-study bias","Imprecision","Incoherence"] |
| B:D | 0 | Some concerns | Low risk | No concerns | No concerns | No concerns | Some concerns | Low | ["Within-study bias","Incoherence"] |
| B:E | 0 | Some concerns | Low risk | No concerns | Major concerns | No concerns | Some concerns | Very low | ["Within-study bias","Imprecision","Incoherence"] |
| B:F | 0 | Some concerns | Low risk | No concerns | Major concerns | No concerns | Some concerns | Very low | ["Within-study bias","Imprecision","Incoherence"] |
| B:G | 0 | Some concerns | Low risk | No concerns | Major concerns | No concerns | Some concerns | Very low | ["Within-study bias","Imprecision","Incoherence"] |
| C:D | 0 | Some concerns | Low risk | No concerns | Major concerns | No concerns | Some concerns | Very low | ["Within-study bias","Imprecision","Incoherence"] |
| C:E | 0 | Some concerns | Low risk | No concerns | Major concerns | No concerns | Some concerns | Very low | ["Within-study bias","Imprecision","Incoherence"] |
| C:F | 0 | Some concerns | Low risk | No concerns | No concerns | Major concerns | Some concerns | Very low | ["Within-study bias","Heterogeneity","Incoherence"] |
| C:G | 0 | Some concerns | Low risk | No concerns | Major concerns | No concerns | Some concerns | Very low | ["Within-study bias","Imprecision","Incoherence"] |
| D:E | 0 | Some concerns | Low risk | No concerns | No concerns | No concerns | Some concerns | Low | ["Within-study bias","Incoherence"] |
| D:F | 0 | Some concerns | Low risk | No concerns | No concerns | No concerns | Some concerns | Low | ["Within-study bias","Incoherence"] |
| D:G | 0 | Some concerns | Low risk | No concerns | Major concerns | No concerns | Some concerns | Very low | ["Within-study bias","Imprecision","Incoherence"] |
| E:F | 0 | No concerns | Low risk | No concerns | Major concerns | No concerns | Some concerns | Low | ["Imprecision","Incoherence"] |
| E:G | 0 | Some concerns | Low risk | No concerns | Major concerns | No concerns | Some concerns | Very low | ["Within-study bias","Imprecision","Incoherence"] |
| F:G | 0 | Some concerns | Low risk | No concerns | Major concerns | No concerns | Some concerns | Very low | ["Within-study bias","Imprecision","Incoherence"] |

Table A.24: GRADE assessment: TER

| Comparison | Number of studies | Within-study bias | Reporting bias | Indirectness | Imprecision | Heterogeneity | Incoherence | Confidence rating | Reason(s) for downgrading |
| --- | --- | --- | --- | --- | --- | --- | --- | --- | --- |
| A:B | 21 | Some concerns | Low risk | No concerns | No concerns | Major concerns | Some concerns | Very low | ["Within-study bias","Heterogeneity","Incoherence"] |
| A:C | 11 | Some concerns | Low risk | No concerns | No concerns | Major concerns | Some concerns | Very low | ["Within-study bias","Heterogeneity","Incoherence"] |
| A:D | 1 | Some concerns | Low risk | No concerns | Major concerns | No concerns | Some concerns | Very low | ["Within-study bias","Imprecision","Incoherence"] |
| A:E | 2 | Some concerns | Low risk | No concerns | Major concerns | No concerns | Some concerns | Very low | ["Within-study bias","Imprecision","Incoherence"] |
| A:F | 1 | Some concerns | Low risk | No concerns | No concerns | Major concerns | Some concerns | Very low | ["Within-study bias","Heterogeneity","Incoherence"] |
| A:G | 3 | Some concerns | Low risk | No concerns | No concerns | Major concerns | Some concerns | Very low | ["Within-study bias","Heterogeneity","Incoherence"] |
| A:H | 2 | No concerns | Low risk | No concerns | Major concerns | No concerns | Some concerns | Low | ["Imprecision","Incoherence"] |
| A:I | 2 | No concerns | Low risk | No concerns | Major concerns | No concerns | Some concerns | Low | ["Imprecision","Incoherence"] |
| A:J | 3 | Some concerns | Low risk | No concerns | No concerns | No concerns | Some concerns | Very low | ["Within-study bias","Incoherence"] |
| A:K | 3 | Some concerns | Low risk | No concerns | Major concerns | No concerns | Some concerns | Very low | ["Within-study bias","Imprecision","Incoherence"] |
| B:C | 0 | Some concerns | Low risk | No concerns | Major concerns | No concerns | Some concerns | Very low | ["Within-study bias","Imprecision","Incoherence"] |
| B:D | 0 | Some concerns | Low risk | No concerns | Major concerns | No concerns | Some concerns | Very low | ["Within-study bias","Imprecision","Incoherence"] |
| B:E | 0 | Some concerns | Low risk | No concerns | Major concerns | No concerns | Some concerns | Very low | ["Within-study bias","Imprecision","Incoherence"] |
| B:F | 0 | Some concerns | Low risk | No concerns | Major concerns | No concerns | Some concerns | Very low | ["Within-study bias","Imprecision","Incoherence"] |
| B:G | 0 | Some concerns | Low risk | No concerns | Major concerns | No concerns | Some concerns | Very low | ["Within-study bias","Imprecision","Incoherence"] |
| B:H | 0 | Some concerns | Low risk | No concerns | Major concerns | No concerns | Some concerns | Very low | ["Within-study bias","Imprecision","Incoherence"] |
| B:I | 0 | No concerns | Low risk | No concerns | Major concerns | No concerns | Some concerns | Low | ["Imprecision","Incoherence"] |
| B:J | 0 | Some concerns | Low risk | No concerns | Major concerns | No concerns | Some concerns | Very low | ["Within-study bias","Imprecision","Incoherence"] |
| B:K | 0 | Some concerns | Low risk | No concerns | Major concerns | No concerns | Some concerns | Very low | ["Within-study bias","Imprecision","Incoherence"] |
| C:D | 0 | Some concerns | Low risk | No concerns | Major concerns | No concerns | Some concerns | Very low | ["Within-study bias","Imprecision","Incoherence"] |
| C:E | 0 | Some concerns | Low risk | No concerns | Major concerns | No concerns | Some concerns | Very low | ["Within-study bias","Imprecision","Incoherence"] |
| C:F | 0 | Some concerns | Low risk | No concerns | Major concerns | No concerns | Some concerns | Very low | ["Within-study bias","Imprecision","Incoherence"] |
| C:G | 0 | Some concerns | Low risk | No concerns | Major concerns | No concerns | Some concerns | Very low | ["Within-study bias","Imprecision","Incoherence"] |
| C:H | 0 | Some concerns | Low risk | No concerns | Major concerns | No concerns | Some concerns | Very low | ["Within-study bias","Imprecision","Incoherence"] |
| C:I | 0 | No concerns | Low risk | No concerns | Major concerns | No concerns | Some concerns | Low | ["Imprecision","Incoherence"] |
| C:J | 0 | Some concerns | Low risk | No concerns | Major concerns | No concerns | Some concerns | Very low | ["Within-study bias","Imprecision","Incoherence"] |
| C:K | 0 | Some concerns | Low risk | No concerns | Major concerns | No concerns | Some concerns | Very low | ["Within-study bias","Imprecision","Incoherence"] |
| D:E | 0 | Some concerns | Low risk | No concerns | Major concerns | No concerns | Some concerns | Very low | ["Within-study bias","Imprecision","Incoherence"] |
| D:F | 0 | Some concerns | Low risk | No concerns | Major concerns | No concerns | Some concerns | Very low | ["Within-study bias","Imprecision","Incoherence"] |
| D:G | 0 | Some concerns | Low risk | No concerns | Major concerns | No concerns | Some concerns | Very low | ["Within-study bias","Imprecision","Incoherence"] |
| D:H | 0 | Some concerns | Low risk | No concerns | Major concerns | No concerns | Some concerns | Very low | ["Within-study bias","Imprecision","Incoherence"] |
| D:I | 0 | Some concerns | Low risk | No concerns | Major concerns | No concerns | Some concerns | Very low | ["Within-study bias","Imprecision","Incoherence"] |
| D:J | 0 | Some concerns | Low risk | No concerns | No concerns | Major concerns | Some concerns | Very low | ["Within-study bias","Incoherence"] |
| D:K | 0 | Some concerns | Low risk | No concerns | Major concerns | No concerns | Some concerns | Very low | ["Within-study bias","Imprecision","Incoherence"] |
| E:F | 0 | Some concerns | Low risk | No concerns | Major concerns | No concerns | Some concerns | Very low | ["Within-study bias","Imprecision","Incoherence"] |
| E:G | 0 | Some concerns | Low risk | No concerns | Major concerns | No concerns | Some concerns | Very low | ["Within-study bias","Imprecision","Incoherence"] |
| E:H | 0 | Some concerns | Low risk | No concerns | Major concerns | No concerns | Some concerns | Very low | ["Within-study bias","Imprecision","Incoherence"] |
| E:I | 0 | Some concerns | Low risk | No concerns | Major concerns | No concerns | Some concerns | Very low | ["Within-study bias","Imprecision","Incoherence"] |
| E:J | 0 | Some concerns | Low risk | No concerns | No concerns | Major concerns | Some concerns | Very low | ["Within-study bias","Heterogeneity","Incoherence"] |
| E:K | 0 | Some concerns | Low risk | No concerns | Major concerns | No concerns | Some concerns | Very low | ["Within-study bias","Imprecision","Incoherence"] |
| F:G | 0 | Some concerns | Low risk | No concerns | Major concerns | No concerns | Some concerns | Very low | ["Within-study bias","Imprecision","Incoherence"] |
| F:H | 0 | Some concerns | Low risk | No concerns | Major concerns | No concerns | Some concerns | Very low | ["Within-study bias","Imprecision","Incoherence"] |
| F:I | 0 | Some concerns | Low risk | No concerns | Major concerns | No concerns | Some concerns | Very low | ["Within-study bias","Imprecision","Incoherence"] |
| F:J | 0 | Some concerns | Low risk | No concerns | Major concerns | No concerns | Some concerns | Very low | ["Within-study bias","Imprecision","Incoherence"] |
| F:K | 0 | Some concerns | Low risk | No concerns | Major concerns | No concerns | Some concerns | Very low | ["Within-study bias","Imprecision","Incoherence"] |
| G:H | 0 | Some concerns | Low risk | No concerns | Major concerns | No concerns | Some concerns | Very low | ["Within-study bias","Imprecision","Incoherence"] |
| G:I | 0 | Some concerns | Low risk | No concerns | Major concerns | No concerns | Some concerns | Very low | ["Within-study bias","Imprecision","Incoherence"] |
| G:J | 0 | Some concerns | Low risk | No concerns | Major concerns | No concerns | Some concerns | Very low | ["Within-study bias","Imprecision","Incoherence"] |
| G:K | 0 | Some concerns | Low risk | No concerns | Major concerns | No concerns | Some concerns | Very low | ["Within-study bias","Imprecision","Incoherence"] |
| H:I | 0 | No concerns | Low risk | No concerns | Major concerns | No concerns | Some concerns | Low | ["Imprecision","Incoherence"] |
| H:J | 0 | Some concerns | Low risk | No concerns | Major concerns | No concerns | Some concerns | Very low | ["Within-study bias","Imprecision","Incoherence"] |
| H:K | 0 | Some concerns | Low risk | No concerns | Major concerns | No concerns | Some concerns | Very low | ["Within-study bias","Imprecision","Incoherence"] |
| I:J | 0 | Some concerns | Low risk | No concerns | Major concerns | No concerns | Some concerns | Very low | ["Within-study bias","Imprecision","Incoherence"] |
| I:K | 0 | Some concerns | Low risk | No concerns | Major concerns | No concerns | Some concerns | Very low | ["Within-study bias","Imprecision","Incoherence"] |
| J:K | 0 | Some concerns | Low risk | No concerns | Major concerns | No concerns | Some concerns | Very low | ["Within-study bias","Imprecision","Incoherence"] |

Appendix Table A.25: SUCRA values

|  | CG | HQ | YX | SX | DC | DS | DH | SK | KD | ST | GG |
| --- | --- | --- | --- | --- | --- | --- | --- | --- | --- | --- | --- |
| UAER | 11.5 | 57.3 | 57.2 | 29.0 | 31.2 | 77.1 | 49.1 | 46.8 | 52.8 | **90.2** | 47.8 |
| BUN | 7.8 | 48.1 | 27.8 | 71.2 | 39.7 | **92.3** | 45.0 | 84.7 | 39.0 | 36.1 | 58.3 |
| Scr | 9.4 | 52.7 | 23.0 | 43.7 | 69.4 | 81.2 | 32.9 | **89.7** | 58.3 | 45.0 | 44.7 |
| β_2_-MG | 9.8 | 51.7 | **84.4** | 49.9 | 39.2 | 35.5 | 61.1 | 66.1 | 57.3 | 44.9 | — |
| TC | 14.8 | 59.4 | 74.5 | 31.5 | 65.2 | 40.7 | 31.6 | 39.4 | — | **85.3** | 57.6 |
| TG | 13.2 | 84.7 | 62.1 | 47.1 | **89.1** | 49.5 | 24.4 | 34.3 | — | 43.3 | 52.3 |
| SBP | 40.1 | 35.9 | 74.5 | **98.9** | 28.6 | 6.5 | — | 65.4 | — | — | — |
| TER | 5.6 | 57.7 | 40.6 | 68.6 | 68.8 | **77.0** | 6.4 | 46.1 | 67.1 | 61.9 | — |

CG, control group; TER, total effective rate; UAER, urinary albumin excretion rate; BUN, blood urea nitrogen; Scr, serum creatinine; β_2_-MG, β_2_-microglobulin; TC, total cholesterol; TG, triglyceride; SBP, systolic blood pressure; ACEI, angiotensin-converting enzyme inhibitor; ARB, angiotensin Ⅱ receptor blockers;

Appendix Table A.26: Adverse reaction

| Number | Study | TCMI | Experimental group | Control group |
| --- | --- | --- | --- | --- |
| 1 | Liu XX 2015 | HQ | None | None |
| 2 | Li SB 2015 | HQ | None | None |
| 3 | Liu XX 2013 | HQ | None | None |
| 4 | Huang SL 2013 | HQ | None | None |
| 5 | Zhang Y 2012 | HQ | None | None |
| 6 | Huan HM 2010 | HQ | None | None |
| 7 | Tian PW 2009 | HQ | None | None |
| 8 | Cui BS 2009 | HQ | None | None |
| 9 | Xu XL 2008 | HQ | None | None |
| 10 | Jiang Lan 2005 | HQ | None | None |
| 11 | Zhang CJ 2013 | YX | 1 case of dizziness; 1 case of cough | 2 cases of dizziness; 2 cases of cough; 1 case of headache |
| 12 | Wang GC 2012 | YX | None | None |
| 13 | Chen JS 2015 | YX | 1 case of rash;  1 case of nausea | 1 case of dizziness; 1 case of cough |
| 14 | Huang JY 2017 | YX | None | None |
| 15 | Li L 2012 | YX | 2 cases of dizziness; 1 case of elevated potassium in blood | 2 cases of elevated potassium in blood |
| 16 | Geng GM 2013 | DC | 2 cases of headache; 1 case of tinnitus; 3 cases of dizziness; 2 cases of postural hypotension | 3 cases of headache; 3 cases of dizziness; 2 cases of postural hypotension; 1 case of tachycardia |
| 17 | Tang N 2019 | DC | 1 case of cough; 2 cases of itchiness | 2 cases of cough; 2 cases of itchiness |
| 18 | Zhang Y 2014 | DC | None | None |
| 19 | Tao Y 2016 | DC | 4 cases of cough | 5 cases of cough |
| 20 | Zhang ZM 2014 | DC | 3 cases of headache, fatigue and postural hypotension | 6 cases of headache, fatigue and postural hypotension |
| 21 | Hu B 2016 | DS | 6 cases of elevated potassium in blood | 19 cases of elevated potassium in blood |
| 22 | Hao JL 2022 | SK | 2 cases of cough; 3 cases of gastrointestinal discomfort | 16 cases of cough; 17 cases of gastrointestinal discomfort; 5 cases of hypotension |
| 23 | Hu YL 2015 | SK | 1 case of dizziness; 4 cases of cough | 1 case of cough |
| 24 | Song LH 2012 | SK | 2 cases of itchiness | None |
| 25 | Rao GF 2013 | KD | 1 case of dizziness; 1 case of elevated potassium in blood | 1 case of dizziness |
| 26 | Jin QJ 2016 | KD | 1 case of dizziness; 1 case of elevated potassium in blood | 1 case of dizziness |
| 27 | Zhang SJ 2023 | KD | 3 cases of nausea; 2 cases of fatigue; 1 case of shortness of breath | 1 case of dizziness; 1 cases of nausea; 1 case of shortness of breath |
| 28 | Qin WG 2010 | ST | 3 cases of cough | 3 cases of cough |
| 29 | Zhu YX 2003 | GG | None | None |
| 30 | Yuan DY 2009 | GG | None | None |

Appendix Table A.27: Egger’s regression test

| **UAER**: Number of studies = 49 | | | | Root MSE = 3.86 | | |
| --- | --- | --- | --- | --- | --- | --- |
| Std_Eff | Coef. | Std. Err. | t | | P>\|t\| | 95% CI |
| Slope bias | 0.70 | 0.53 | 1.32 | | 0.19 | -0.37, 1.77 |
|  | -8.31 | 2.05 | -0.46 | | <0.01 | -12.43, -4.20 |
| **Scr**: Number of studies = 78 | | | | Root MSE = 4.04 | | |
| Std_Eff | Coef. | Std. Err. | t | | P>\|t\| | 95% CI |
| Slope bias | -0.46 | 0.35 | -1.31 | | 0.19 | -1.16, 0.24 |
|  | -1.52 | 1.63 | -0.93 | | 0.36 | -4.76, 1.77 |
| **BUN**: Number of studies = 62 | | | | Root MSE = 4.17 | | |
| Std_Eff | Coef. | Std. Err. | t | | P>\|t\| | 95% CI |
| Slope bias | -0.21 | 0.48 | -0.44 | | 0.66 | -1.16, 0.75 |
|  | -2.37 | 2.19 | -1.08 | | 0.29 | -6.75, 2.02 |
| **β_2_-MG**: Number of studies = 28 | | | | Root MSE = 4.41 | | |
| Std_Eff | Coef. | Std. Err. | t | | P>\|t\| | 95% CI |
| Slope bias | -0.65 | 0.66 | -0.99 | | 0.33 | -2.00, 0.70 |
|  | -2.77 | 3.01 | -0.92 | | 0.37 | -8.96, 3.43 |
| **TC**: Number of studies =21 | | | | Root MSE = 4.10 | | |
| Std_Eff | Coef. | Std. Err. | t | | P>\|t\| | 95% CI |
| Slope bias | -0.19 | 0.60 | -0.31 | | 0.76 | -1.45, 1.07 |
|  | -5.31 | 2.72 | -1.95 | | 0.07 | -11.00, 0.38 |
| **TG**: Number of studies =24 | | | | Root MSE =4.23 | | |
| Std_Eff | Coef. | Std. Err. | t | | P>\|t\| | 95% CI |
| Slope bias | -0.72 | 0.74 | -0.98 | | 0.34 | -2.25, 0.80 |
|  | -1.93 | 3.24 | -0.60 | | 0.56 | -8.66, 4.79 |
| **SBP**: Number of studies = 18 | | | | Root MSE = 3.26 | | |
| Std_Eff | Coef. | Std. Err. | t | | P>\|t\| | 95% CI |
| Slope bias | 1.18 | 0.55 | 2.15 | | 0.05 | 0.02, 2.33 |
|  | -6.00 | 2.60 | -2.31 | | 0.04 | -11.50, -0.49 |
| **TER**: Number of studies = 35 | | | | Root MSE = 0.81 | | |
| Std_Eff | Coef. | Std. Err. | t | | P>\|t\| | 95% CI |
| Slope bias | 0.04 | 0.03 | 1.46 | | 0.15 | -0.02, 0.09 |
|  | 1.75 | 0.35 | 4.95 | | <0.01 | 1.03, 2.48 |

Appendix Table A.28-A.35: Heterogeneity analysis

Table A.28: Heterogeneity analysis: UAER

| Study | SMD (95% CI) | Weight (%) |
| --- | --- | --- |
| Zhang YX (2021) | -2.794 (-3.348, -2.24) | 2.07 |
| Zhang X (2017) | -0.606 (-0.907, -0.306) | 2.16 |
| Ren YJ (2016) | -1.635 (-2.114, -1.157) | 2.10 |
| Cai R (2016) | -2.333 (-2.904, -1.762) | 2.06 |
| Liu XX (2015) | -0.953 (-1.442, -0.465) | 2.10 |
| Liu S (2015) | -0.623 (-1.16, -0.086) | 2.08 |
| Li SB (2015) | -2.752 (-3.465, -2.04) | 1.99 |
| Zhang Q (2014) | -6.507 (-7.842, -5.173) | 1.61 |
| Sun YH (2014) | -1.768 (-2.301, -1.236) | 2.08 |
| Liu XX (2013) | -1.324 (-1.819, -0.83) | 2.10 |
| Chen XX (2012) | -0.628 (-1.083, -0.173) | 2.11 |
| Zhu LY (2011) | -3.758 (-4.639, -2.876) | 1.90 |
| Zeng J (2010) | -0.197 (-0.742, 0.348) | 2.07 |
| Li ZQ (2010) | -2.119 (-2.715, -1.523) | 2.05 |
| Xu ZX (2009) | -0.67 (-1.209, -0.132) | 2.08 |
| Tian PW (2009) | -1.37 (-1.858, -0.882) | 2.10 |
| Cui BS (2009) | -2.001 (-2.57, -1.432) | 2.06 |
| Xu XL (2008) | -2.246 (-2.809, -1.683) | 2.07 |
| Jiang Lan (2005) | -0.559 (-1.067, -0.051) | 2.09 |
| Li HJ (2007) | -0.598 (-1.061, -0.136) | 2.11 |
| Ma CH (2006) | -3.237 (-3.698, -2.776) | 2.11 |
| Shi XH (2010) | -1.598 (-2.172, -1.024) | 2.06 |
| Zhang CJ (2013) | -4.562 (-5.653, -3.472) | 1.77 |
| Chen JS (2015) | 2.978 (2.356, 3.599) | 2.04 |
| Wang Qz (2011) | -1.535 (-2.078, -0.993) | 2.08 |
| Xing LY (2022) | -3.445 (-3.888, -3.002) | 2.12 |
| Shen SL (2017) | -1.41 (-1.978, -0.843) | 2.06 |
| Zhang YM (2015) | -1.711 (-2.104, -1.317) | 2.13 |
| Liu YX (2019) | -4.133 (-5.024, -3.242) | 1.89 |
| Cheng H (2018) | -2.637 (-3.12, -2.155) | 2.10 |
| Li L (2012) | -1.4 (-1.967, -0.833) | 2.07 |
| Li SH (2009) | -0.743 (-1.063, -0.422) | 2.16 |
| Zhen D (2012) | -0.392 (-0.858, 0.075) | 2.11 |
| Xian LH (2015) | -0.524 (-0.982, -0.067) | 2.11 |
| Tao L (2015) | -0.867 (-1.372, -0.361) | 2.09 |
| Zhou AJ (2009) | -3.152 (-3.917, -2.387) | 1.96 |
| Wang CQ (2012) | -0.755 (-1.279, -0.23) | 2.08 |
| Ye XA (2016) | -2.614 (-3.145, -2.083) | 2.08 |
| Li JJ (2009) | -1.158 (-1.627, -0.689) | 2.11 |
| Li HF (2022) | -1.378 (-1.843, -0.912) | 2.11 |
| Hu YL (2015) | -1.406 (-1.923, -0.888) | 2.09 |
| Rao GF (2013) | -1.283 (-1.604, -0.962) | 2.16 |
| Zhang SJ (2023) | -2.072 (-2.559, -1.584) | 2.10 |
| Li HF (2010) | -10.441 (-12.407, -8.474) | 1.22 |
| Sun XP (2015) | -1.632 (-2.175, -1.089) | 2.08 |
| Qin WG (2010) | -0.533 (-0.979, -0.087) | 2.12 |
| Zhu YX (2003) | -0.448 (-0.96, 0.065) | 2.09 |
| Xu JQ (2011) | -3.79 (-4.845, -2.734) | 1.79 |
| Jiao FZ (2011) | -0.282 (-0.687, 0.123) | 2.13 |
| D+L pooled SMD | -1.747 (-2.074, -1.421) | 100 |
| Heterogeneity chi-squared = 946.45 (d.f. = 48) P <0.001 | | |
| I-squared (variation in SMD attributable to heterogeneity) = 94.9% | | |
| Estimate of between-study variance Tau-squared = 1.2615 | | |
| Test of SMD=0 : z= 10.48 P<0.001 | | |
| Notes：Weights are from random effects analysis | | |

Table A.29: Heterogeneity analysis: Scr

| Study | SMD (95% CI) | Weight (%) |
| --- | --- | --- |
| Zhang SB (2020) | -4.46 (-5.286, -3.634) | 1.11 |
| Qiao MF (2018) | -0.548 (-0.994, -0.101) | 1.3 |
| Zhang X (2017) | -0.713 (-1.016, -0.41) | 1.35 |
| Li H (2017) | -1.971 (-2.428, -1.514) | 1.29 |
| Tang H (2016) | -0.864 (-1.206, -0.522) | 1.33 |
| Ren YJ (2016) | -0.63 (-1.054, -0.207) | 1.31 |
| Liu SY (2016) | -0.686 (-1.155, -0.216) | 1.29 |
| Wang XH (2015) | -2.384 (-2.879, -1.888) | 1.28 |
| Liu XX (2015) | -0.07 (-0.532, 0.392) | 1.29 |
| Liu S (2015) | -0.833 (-1.38, -0.286) | 1.26 |
| Zhang Q (2014) | -2.106 (-2.764, -1.449) | 1.2 |
| Sun YH (2014) | -0.314 (-0.767, 0.138) | 1.3 |
| Huang SL (2013) | -0.399 (-0.795, -0.003) | 1.32 |
| Zhang Y (2012) | -0.262 (-0.77, 0.246) | 1.27 |
| Chen XX (2012) | -0.499 (-0.95, -0.048) | 1.3 |
| Zhu LY (2011) | 0.563 (0.029, 1.098) | 1.26 |
| Zeng J (2010) | -0.017 (-0.56, 0.527) | 1.26 |
| Li ZQ (2010) | -0.155 (-0.633, 0.324) | 1.28 |
| Huan HM (2010) | 0.399 (-0.227, 1.025) | 1.22 |
| Geng F (2010) | -3.53 (-4.348, -2.712) | 1.12 |
| Xu ZX (2009) | 0.053 (-0.471, 0.576) | 1.27 |
| Cui BS (2009) | -0.166 (-0.629, 0.296) | 1.29 |
| Xu XL (2008) | -0.033 (-0.472, 0.406) | 1.3 |
| Chen JN (2010) | -0.352 (-0.725, 0.022) | 1.32 |
| Jiang Lan (2005) | -0.62 (-1.13, -0.109) | 1.27 |
| Li HJ (2007) | -0.268 (-0.722, 0.186) | 1.29 |
| Huang W (2004) | -0.184 (-0.7, 0.333) | 1.27 |
| Ma CH (2006) | -2.562 (-2.971, -2.153) | 1.31 |
| Shi XH (2010) | 0.24 (-0.26, 0.739) | 1.28 |
| Zhang CJ (2013) | -0.149 (-0.715, 0.418) | 1.25 |
| Chen JS (2015) | 4.148 (3.387, 4.909) | 1.15 |
| Shen SL (2017) | -0.351 (-0.861, 0.159) | 1.27 |
| Zhang YM (2015) | -0.456 (-0.797, -0.116) | 1.34 |
| Li Hong (2014) | -0.855 (-1.694, -0.016) | 1.11 |
| Liu YX (2019) | -1.51 (-2.076, -0.943) | 1.25 |
| Huang JY (2017) | -1.581 (-1.873, -1.288) | 1.35 |
| Cheng H (2018) | 0.179 (-0.174, 0.532) | 1.33 |
| Zhu J (2020) | -0.893 (-1.425, -0.362) | 1.26 |
| You Wen (2014) | -0.643 (-1.178, -0.108) | 1.26 |
| Li L (2012) | -0.773 (-1.298, -0.247) | 1.26 |
| Li SH (2009) | -0.592 (-0.909, -0.275) | 1.34 |
| Li ZY (2013) | 0.082 (-0.317, 0.48) | 1.32 |
| Wu GY (2017) | -0.447 (-0.959, 0.066) | 1.27 |
| Yang QH (2007) | -0.335 (-0.851, 0.182) | 1.27 |
| Xu YY (2020) | -0.43 (-0.858, -0.003) | 1.3 |
| Zhen D (2012) | -1.368 (-1.883, -0.854) | 1.27 |
| Geng GM (2013) | -0.934 (-1.409, -0.46) | 1.29 |
| Tang N (2019) | -2.009 (-2.368, -1.649) | 1.33 |
| Gong J (2022) | -1.748 (-2.16, -1.337) | 1.31 |
| Zhang Y (2014) | -1.482 (-1.979, -0.986) | 1.28 |
| Fan WK (2017) | -0.035 (-0.355, 0.285) | 1.34 |
| Wei HB (2016) | -0.85 (-1.256, -0.445) | 1.31 |
| Hu B (2016) | -0.292 (-0.497, -0.086) | 1.37 |
| Xian XW (2014) | -3.895 (-4.566, -3.223) | 1.2 |
| Li QY (2011) | -0.685 (-1.136, -0.234) | 1.3 |
| Huang YJ (2016) | -0.051 (-0.451, 0.349) | 1.31 |
| Ye XA (2016) | 0 (-0.388, 0.388) | 1.32 |
| Li JJ (2009) | -0.292 (-0.727, 0.143) | 1.3 |
| Liu W (2009) | -0.53 (-0.886, -0.175) | 1.33 |
| Jing XN (2014) | -1.037 (-1.415, -0.658) | 1.32 |
| Tao GL (2019) | -0.454 (-0.967, 0.059) | 1.27 |
| He LF (2020) | -6.062 (-6.799, -5.325) | 1.16 |
| Hao JL (2022) | -1.153 (-1.39, -0.917) | 1.36 |
| Ren LL (2018) | -1.537 (-1.984, -1.089) | 1.3 |
| Li HF (2022) | -1.459 (-1.93, -0.987) | 1.29 |
| Deng ZH (2021) | -1.42 (-1.745, -1.094) | 1.34 |
| Hu YL (2015) | -0.319 (-0.783, 0.146) | 1.29 |
| Ma YQ (2019) | -1.105 (-1.549, -0.661) | 1.3 |
| Chen P (2021) | -2.752 (-3.42, -2.084) | 1.2 |
| Rao GF (2013) | -0.58 (-0.878, -0.281) | 1.35 |
| Jin QJ (2016) | -1.141 (-1.364, -0.917) | 1.37 |
| Zhang SJ (2023) | -1.307 (-1.74, -0.875) | 1.3 |
| Li HF (2010) | -0.178 (-0.685, 0.329) | 1.27 |
| Sun XP (2015) | -1.092 (-1.595, -0.589) | 1.27 |
| Qin WG (2010) | -0.78 (-1.235, -0.325) | 1.29 |
| Xu JQ (2011) | 0.123 (-0.5, 0.747) | 1.22 |
| Yuan DY (2009) | -0.228 (-0.657, 0.202) | 1.3 |
| Jiao FZ (2011) | -1.904 (-2.39, -1.417) | 1.28 |
| D+L pooled SMD | -0.839 (-1.034, -0.643) | 100 |
| Heterogeneity chi-squared = 1254.78 (d.f. = 77) P<0.001 | | |
| I-squared (variation in SMD attributable to heterogeneity) = 93.9% | | |
| Estimate of between-study variance Tau-squared = 0.7154 | | |
| Test of SMD=0 : z= 8.41 P<0.001 | | |
| Notes：Weights are from random effects analysis | | |

Table A.30: Heterogeneity analysis: BUN

| Study | MD (95% CI) | Weight (%) |
| --- | --- | --- |
| Zhang SB (2020) | -3.094 (-3.748, -2.44) | 1.52 |
| Qiao MF (2018) | -0.522 (-0.968, -0.076) | 1.62 |
| Zhang X (2017) | -1.963 (-2.321, -1.605) | 1.66 |
| Li H (2017) | -0.93 (-1.323, -0.536) | 1.65 |
| Tang H (2016) | -0.705 (-1.042, -0.368) | 1.67 |
| Wang XH (2015) | -0.756 (-1.147, -0.366) | 1.65 |
| Zhang Q (2014) | -1.297 (-1.875, -0.719) | 1.56 |
| Sun YH (2014) | -0.2 (-0.65, 0.251) | 1.62 |
| Huang SL (2013) | -0.438 (-0.835, -0.041) | 1.65 |
| Chen XX (2012) | 0.539 (0.087, 0.991) | 1.62 |
| Zhu LY (2011) | -0.073 (-0.597, 0.451) | 1.59 |
| Zeng J (2010) | -0.043 (-0.586, 0.501) | 1.58 |
| Huan HM (2010) | -0.059 (-0.679, 0.561) | 1.54 |
| Geng F (2010) | -0.313 (-0.822, 0.197) | 1.59 |
| Xu ZX (2009) | 0.157 (-0.367, 0.682) | 1.59 |
| Cui BS (2009) | -0.574 (-1.046, -0.103) | 1.61 |
| Xu XL (2008) | -0.09 (-0.529, 0.349) | 1.63 |
| Chen JN (2010) | -0.402 (-0.777, -0.028) | 1.65 |
| Jiang Lan (2005) | -0.298 (-0.799, 0.203) | 1.60 |
| Li HJ (2007) | -0.132 (-0.584, 0.321) | 1.62 |
| Huang W (2004) | -0.611 (-1.138, -0.083) | 1.59 |
| Ma CH (2006) | -2.568 (-2.978, -2.158) | 1.64 |
| Shi XH (2010) | -0.121 (-0.619, 0.377) | 1.60 |
| Zhang CJ (2013) | -0.303 (-0.873, 0.266) | 1.56 |
| Chen JS (2015) | 2.101 (1.569, 2.633) | 1.58 |
| Shen SL (2017) | -0.204 (-0.712, 0.303) | 1.60 |
| Huang JY (2017) | -1.213 (-1.491, -0.936) | 1.69 |
| Zhu J (2020) | -0.788 (-1.314, -0.262) | 1.59 |
| You Wen (2014) | 0.223 (-0.3, 0.746) | 1.59 |
| Li L (2012) | -0.265 (-0.774, 0.243) | 1.59 |
| Li SH (2009) | -0.827 (-1.15, -0.504) | 1.67 |
| Li ZY (2013) | 0 (-0.398, 0.398) | 1.65 |
| Yang QH (2007) | -0.1 (-0.614, 0.413) | 1.59 |
| Xu YY (2020) | -2.79 (-3.387, -2.193) | 1.55 |
| Zhen D (2012) | -1.657 (-2.195, -1.12) | 1.58 |
| Geng GM (2013) | -0.159 (-0.61, 0.291) | 1.62 |
| Tang N (2019) | -1.782 (-2.128, -1.435) | 1.67 |
| Gong J (2022) | -1.777 (-2.19, -1.363) | 1.64 |
| Zhang Y (2014) | -1.573 (-2.076, -1.07) | 1.60 |
| Tao Y (2016) | 0 (-0.31, 0.31) | 1.68 |
| Fan WK (2017) | -0.042 (-0.362, 0.278) | 1.67 |
| Wei HB (2016) | -0.683 (-1.083, -0.284) | 1.64 |
| Xian XW (2014) | -2.82 (-3.376, -2.264) | 1.57 |
| Huang YJ (2016) | -0.153 (-0.553, 0.248) | 1.64 |
| Ye XA (2016) | -0.069 (-0.457, 0.32) | 1.65 |
| Li JJ (2009) | -0.058 (-0.491, 0.375) | 1.63 |
| Liu W (2009) | -1.584 (-1.985, -1.182) | 1.64 |
| Jing XN (2014) | -1.055 (-1.434, -0.676) | 1.65 |
| Tao GL (2019) | 0.482 (-0.032, 0.996) | 1.59 |
| Ren LL (2018) | -2.827 (-3.384, -2.27) | 1.57 |
| Li HF (2022) | -1.207 (-1.662, -0.752) | 1.62 |
| Deng ZH (2021) | -3.809 (-4.298, -3.32) | 1.60 |
| Hu YL (2015) | -0.356 (-0.822, 0.109) | 1.62 |
| Chen P (2021) | -4.367 (-5.251, -3.483) | 1.38 |
| Rao GF (2013) | -0.414 (-0.71, -0.119) | 1.68 |
| Jin QJ (2016) | -0.356 (-0.565, -0.147) | 1.71 |
| Zhang SJ (2023) | -1.385 (-1.822, -0.948) | 1.63 |
| Li HF (2010) | -0.174 (-0.681, 0.333) | 1.60 |
| Qin WG (2010) | -0.253 (-0.693, 0.187) | 1.63 |
| Xu JQ (2011) | -0.193 (-0.818, 0.431) | 1.53 |
| Yuan DY (2009) | -0.225 (-0.654, 0.204) | 1.63 |
| Jiao FZ (2011) | -1.481 (-1.937, -1.026) | 1.62 |
| D+L pooled SMD | -0.773 (-1.001, -0.545) | 100 |
| Heterogeneity chi-squared = 1063.57 (d.f. = 61) P<0.001 | | |
| I-squared (variation in SMD attributable to heterogeneity) = 94.3% | | |
| Estimate of between-study variance Tau-squared = 0.7832 | | |
| Test of SMD=0 : z= 6.64 P<0.001 | | |
| Notes：Weights are from random effects analysis | | |

Table A.31: Heterogeneity analysis: β_2_-MG

| Study | SMD (95% CI) | Weight (%) |
| --- | --- | --- |
| Zhang YX (2021) | -2.00 (-2.482, -1.518) | 3.57 |
| Zhang X (2017) | -1.561 (-1.897, -1.225) | 3.69 |
| Tang H (2016) | -1.093 (-1.444, -0.743) | 3.68 |
| Ren YJ (2016) | -2.683 (-3.256, -2.111) | 3.48 |
| Cai R (2016) | -0.962 (-1.425, -0.498) | 3.59 |
| Liu XX (2013) | -0.752 (-1.214, -0.289) | 3.59 |
| Chen XX (2012) | 0.104 (-0.34, 0.548) | 3.61 |
| Zeng J (2010) | -1.839 (-2.492, -1.187) | 3.39 |
| Xu ZX (2009) | -0.227 (-0.753, 0.298) | 3.53 |
| Tian PW (2009) | -0.907 (-1.368, -0.446) | 3.59 |
| Wang QZ (2011) | -0.392 (-0.872, 0.088) | 3.57 |
| Shen SL (2017) | -2.017 (-2.642, -1.392) | 3.42 |
| Zhang YM (2015) | -4.111 (-4.707, -3.515) | 3.45 |
| Wu GY (2017) | -1.104 (-1.649, -0.56) | 3.51 |
| Zhang Y (2014) | -1.033 (-1.501, -0.566) | 3.58 |
| Tao L (2015) | -0.579 (-1.072, -0.086) | 3.56 |
| Zhou AJ (2009) | -0.583 (-1.1, -0.066) | 3.54 |
| Wang CQ (2012) | -0.519 (-1.034, -0.004) | 3.54 |
| Huang YJ (2016) | -3.53 (-4.174, -2.886) | 3.40 |
| Ye XA (2016) | -0.337 (-0.728, 0.054) | 3.65 |
| Hao JL (2022) | -3.153 (-3.482, -2.824) | 3.70 |
| Wang AY (2013) | -0.975 (-1.487, -0.464) | 3.54 |
| Ren LL (2018) | -1.94 (-2.417, -1.463) | 3.58 |
| Xiao L (2013) | -0.118 (-0.594, 0.358) | 3.58 |
| Rao GF (2013) | -0.993 (-1.303, -0.683) | 3.71 |
| Jin QJ (2016) | -0.67 (-0.883, -0.457) | 3.76 |
| Zheng JJ (2013) | -2.316 (-2.682, -1.95) | 3.67 |
| Li HF (2010) | -0.965 (-1.501, -0.429) | 3.52 |
| D+L pooled SMD | -1.322 (-1.683, -0.961) | 100 |
| Heterogeneity chi-squared = 521.02 (d.f. = 27) P <0.001 | | |
| I-squared (variation in SMD attributable to heterogeneity) = 94.8% | | |
| Estimate of between-study variance Tau-squared = 0.8878 | | |
| Test of SMD=0 : z= 7.18 P <0.001 | | |
| Notes：Weights are from random effects analysis | | |

Table A.32: Heterogeneity analysis: TC

| Study | MD (95% CI) | Weight (%) |
| --- | --- | --- |
| Qiao MF (2018) | -1.609 (-2.115, -1.103) | 4.79 |
| Ren YJ (2016) | -5.83 (-6.786, -4.875) | 4.09 |
| Liu XX (2015) | -0.503 (-0.973, -0.034) | 4.83 |
| Geng F (2010) | -1.071 (-1.613, -0.529) | 4.74 |
| Xu ZX (2009) | -0.029 (-0.553, 0.495) | 4.77 |
| Xu XL (2008) | -1.392 (-1.882, -0.902) | 4.81 |
| Zhang YM (2015) | -4.353 (-4.973, -3.733) | 4.63 |
| Li Hong (2014) | -1.569 (-2.494, -0.644) | 4.14 |
| Huang JY (2017) | -1.509 (-1.799, -1.22) | 5.02 |
| Li SH (2009) | -1.77 (-2.137, -1.404) | 4.95 |
| Zhen D (2012) | -0.422 (-0.889, 0.045) | 4.84 |
| Geng GM (2013) | -2.092 (-2.654, -1.53) | 4.72 |
| Gong J (2022) | -1.856 (-2.275, -1.438) | 4.89 |
| Hu B (2016) | -0.9 (-1.114, -0.685) | 5.07 |
| Jing XN (2014) | -0.481 (-0.841, -0.121) | 4.95 |
| Tao GL (2019) | -0.174 (-0.681, 0.334) | 4.79 |
| Li HF (2022) | -1.475 (-1.947, -1.002) | 4.83 |
| Xiao L (2013) | -1.761 (-2.323, -1.198) | 4.72 |
| Song LH (2012) | -0.298 (-0.747, 0.15) | 4.86 |
| Li B (2013) | -3.25 (-3.769, -2.732) | 4.77 |
| Yuan DY (2009) | -1.7 (-2.2, -1.199) | 4.8 |
| D+L pooled SMD | -1.582 (-2.009, -1.155) | 100 |
| Heterogeneity chi-squared = 383.39 (d.f. = 20) P <0.001 | | |
| I-squared (variation in SMD attributable to heterogeneity) = 94.8% | | |
| Estimate of between-study variance Tau-squared = 0.9257 | | |
| Test of SMD=0 : z= 7.26 P <0.001 | | |
| Notes：Weights are from random effects analysis | | |

Table A.33: Heterogeneity analysis: TG

| Study | MD (95% CI) | Weight (%) |
| --- | --- | --- |
| Qiao MF (2018) | -1.663 (-2.173, -1.153) | 4.16 |
| Ren YJ (2016) | -2.439 (-2.988, -1.891) | 4.11 |
| Liu XX (2015) | -0.473 (-0.941, -0.004) | 4.21 |
| Huang SL (2013) | -0.883 (-1.294, -0.472) | 4.27 |
| Geng F (2010) | -2.18 (-2.823, -1.537) | 3.99 |
| Xu ZX (2009) | -0.059 (-0.583, 0.465) | 4.14 |
| Xu XL (2008) | -1.317 (-1.802, -0.832) | 4.19 |
| Zhang CJ (2013) | -0.138 (-0.704, 0.429) | 4.09 |
| Zhang YM (2015) | -0.496 (-0.837, -0.154) | 4.34 |
| Li Hong (2014) | -1.486 (-2.398, -0.573) | 3.59 |
| Li SH (2009) | -1.966 (-2.345, -1.588) | 4.3 |
| Zhen D (2012) | -0.682 (-1.158, -0.207) | 4.2 |
| Geng GM (2013) | -1.824 (-2.361, -1.287) | 4.13 |
| Tang N (2019) | -3.053 (-3.485, -2.621) | 4.25 |
| Gong J (2022) | -5.332 (-6.081, -4.582) | 3.84 |
| Hu B (2016) | -1.578 (-1.812, -1.344) | 4.42 |
| Wang CQ (2012) | -0.271 (-0.779, 0.238) | 4.16 |
| Jing XN (2014) | -0.078 (-0.433, 0.277) | 4.32 |
| Tao GL (2019) | -0.518 (-1.033, -0.004) | 4.16 |
| Li HF (2022) | -1.019 (-1.464, -0.574) | 4.24 |
| Xiao L (2013) | -0.527 (-1.011, -0.043) | 4.19 |
| Song LH (2012) | -0.226 (-0.673, 0.222) | 4.23 |
| Li B (2013) | -0.887 (-1.317, -0.458) | 4.25 |
| Yuan DY (2009) | -1 (-1.454, -0.546) | 4.22 |
| D+L pooled SMD | -1.236 (-1.621, -0.85) | 100 |
| Heterogeneity chi-squared = 400.45 (d.f. = 23) P <0.001 | | |
| I-squared (variation in SMD attributable to heterogeneity) = 94.3% | | |
| Estimate of between-study variance Tau-squared = 0.8606 | | |
| Test of SMD=0 : z= 6.29 P <0.001 | | |
| Notes：Weights are from random effects analysis | | |

Table A.34: Heterogeneity analysis: SBP

| Study | SMD (95% CI) | Weight (%) |
| --- | --- | --- |
| Chen XX (2012) | 0.225 (-0.22, 0.67) | 5.61 |
| Zeng J (2010) | -0.131 (-0.676, 0.413) | 5.38 |
| Huan HM (2010) | -0.108 (-0.728, 0.512) | 5.2 |
| Xu ZX (2009) | 0.434 (-0.096, 0.965) | 5.42 |
| Cui BS (2009) | 0 (-0.462, 0.462) | 5.57 |
| Jiang L (2005) | 0.108 (-0.391, 0.606) | 5.49 |
| Li HJ (2007) | 0.091 (-0.361, 0.544) | 5.59 |
| Huang W (2004) | -0.156 (-0.672, 0.36) | 5.45 |
| Ma CH (2006) | -1.233 (-1.563, -0.903) | 5.83 |
| Zhang CJ (2013) | -1.027 (-1.631, -0.424) | 5.24 |
| Cheng H (2018) | -0.44 (-0.796, -0.083) | 5.78 |
| Li ZY (2013) | -0.248 (-0.648, 0.151) | 5.7 |
| Zhen D (2012) | -2.085 (-2.662, -1.509) | 5.3 |
| Geng GM (2013) | -0.543 (-1.001, -0.085) | 5.58 |
| Tao Y (2016) | 1.091 (0.758, 1.423) | 5.82 |
| Zhou AJ (2009) | -0.242 (-0.75, 0.266) | 5.47 |
| Hu B (2016) | 0.731 (0.52, 0.942) | 6 |
| Song LH (2012) | -0.558 (-1.013, -0.103) | 5.59 |
| D+L pooled SMD | -0.217 (-0.581, 0.146) | 100 |
| Heterogeneity chi-squared = 225.94 (d.f. = 17) P <0.001 | | |
| I-squared (variation in SMD attributable to heterogeneity) = 92.5% | | |
| Estimate of between-study variance Tau-squared = 0.5625 | | |
| Test of SMD=0 : z= 1.17 P = 0.242 | | |
| Notes：Weights are from random effects analysis | | |

Table A.35: Heterogeneity analysis: TER

| Study | OR (95%CI) | Weight (%) |
| --- | --- | --- |
| Zhang SB (2020) | 1.194 (0.988, 1.442) | 1.94 |
| Qiao MF (2018) | 1.267 (1.045, 1.536) | 1.87 |
| Zhang X (2017) | 1.258 (1.074, 1.473) | 2.70 |
| Liu SY (2016) | 1.333 (1.019, 1.745) | 0.99 |
| Cai R (2016) | 1.147 (0.998, 1.319) | 3.37 |
| Liu S (2015) | 1.238 (0.977, 1.57) | 1.26 |
| Zhang Y (2012) | 1.389 (0.995, 1.938) | 0.65 |
| Zhang CJ (2013) | 1.1 (0.886, 1.365) | 1.51 |
| Wang GC (2012) | 1.139 (1.012, 1.281) | 4.5 |
| Chen JS (2015) | 1.229 (1.002, 1.507) | 1.68 |
| Xing LY (2022) | 1.093 (1.004, 1.189) | 7.60 |
| Zhang YM (2015) | 1.216 (1.04, 1.421) | 2.75 |
| Li Hong (2014) | 1.375 (0.89, 2.124) | 0.39 |
| Huang JY (2017) | 1.102 (0.999, 1.216) | 6.04 |
| Zhu J (2020) | 1.35 (1.021, 1.786) | 0.92 |
| Wu GY (2017) | 1.227 (0.959, 1.57) | 1.17 |
| Yang QH (2007) | 1.429 (1.047, 1.95) | 0.75 |
| Tang N (2019) | 1.137 (1.027, 1.258) | 5.74 |
| Tao Y (2016) | 1.154 (1.024, 1.3) | 4.39 |
| Zhang ZM (2014) | 1.44 (1.109, 1.87) | 1.05 |
| Fan WK (2017) | 1.34 (1.121, 1.601) | 2.16 |
| Wei HB (2016) | 1.406 (1.113, 1.777) | 1.29 |
| Xian XW (2014) | 1.195 (1.043, 1.369) | 3.52 |
| Tian YH (2015) | 1.163 (0.983, 1.375) | 2.41 |
| Liu W (2009) | 1.002 (0.776, 1.295) | 1.09 |
| Tao GL (2019) | 1.3 (0.973, 1.736) | 0.86 |
| Hao JL (2022) | 1.176 (1.07, 1.292) | 6.47 |
| Ren LL (2018) | 1.303 (1.037, 1.637) | 1.35 |
| Deng ZH (2021) | 1.101 (1.005, 1.207) | 6.76 |
| Ma YQ (2019) | 1.171 (0.978, 1.404) | 2.10 |
| Rao GF (2013) | 1.137 (1.012, 1.277) | 4.59 |
| Jin QJ (2016) | 1.188 (1.085, 1.302) | 6.77 |
| Zheng JJ (2013) | 1.766 (1.419, 2.198) | 1.46 |
| Zhang SJ (2023) | 1.171 (1.016, 1.349) | 3.27 |
| Li B (2013) | 1.13 (1.007, 1.268) | 4.67 |
| D+L pooled RR | 1.18 (1.148, 1.212) | 100 |
| Heterogeneity chi-squared = 37.99 (d.f. = 34) P = 0.292 | | |
| I-squared (variation in RR attributable to heterogeneity) = 10.5% | | |
| Estimate of between-study variance Tau-squared = 0.0007 | | |
| Test of RR=1 : z= 11.89 P<0.001 | | |
| Notes：Weights are from random effects analysis | | |

Appendix Table A.36-A.38: Subgroup analysis

Table A.36: Subgroup analysis: UAER

League table--Disease Staging: III

| ST | 1.95 (-0.34,4.23) | 2.10 (-0.45,4.65) | 2.14 (-0.78,5.06) | 2.25 (-1.83,6.32) | 2.36 (-1.70,6.42) | 3.12 (-0.95,7.19) | 3.36 (-0.71,7.42) | 3.64 (1.52,5.75) |
| --- | --- | --- | --- | --- | --- | --- | --- | --- |
| -1.95 (-4.23,0.34) | HQ | 0.15 (-1.52,1.82) | 0.19 (-2.00,2.39) | 0.30 (-3.30,3.89) | 0.41 (-3.16,3.98) | 1.17 (-2.42,4.76) | 1.41 (-2.17,4.99) | 1.69 (0.81,2.56) |
| -2.10 (-4.65,0.45) | -0.15 (-1.82,1.52) | YX | 0.04 (-2.42,2.51) | 0.15 (-3.62,3.91) | 0.26 (-3.49,4.01) | 1.02 (-2.74,4.78) | 1.26 (-2.50,5.01) | 1.54 (0.11,2.96) |
| -2.14 (-5.06,0.78) | -0.19 (-2.39,2.00) | -0.04 (-2.51,2.42) | DH | 0.10 (-3.92,4.13) | 0.22 (-3.79,4.22) | 0.98 (-3.04,5.00) | 1.21 (-2.80,5.23) | 1.49 (-0.52,3.51) |
| -2.25 (-6.32,1.83) | -0.30 (-3.89,3.30) | -0.15 (-3.91,3.62) | -0.10 (-4.13,3.92) | SK | 0.11 (-4.80,5.03) | 0.87 (-4.06,5.80) | 1.11 (-3.81,6.03) | 1.39 (-2.10,4.88) |
| -2.36 (-6.42,1.70) | -0.41 (-3.98,3.16) | -0.26 (-4.01,3.49) | -0.22 (-4.22,3.79) | -0.11 (-5.03,4.80) | KD | 0.76 (-4.15,5.67) | 1.00 (-3.91,5.90) | 1.28 (-2.19,4.74) |
| -3.12 (-7.19,0.95) | -1.17 (-4.76,2.42) | -1.02 (-4.78,2.74) | -0.98 (-5.00,3.04) | -0.87 (-5.80,4.06) | -0.76 (-5.67,4.15) | DC | 0.24 (-4.68,5.16) | 0.52 (-2.96,4.00) |
| -3.36 (-7.42,0.71) | -1.41 (-4.99,2.17) | -1.26 (-5.01,2.50) | -1.21 (-5.23,2.80) | -1.11 (-6.03,3.81) | -1.00 (-5.90,3.91) | -0.24 (-5.16,4.68) | GG | 0.28 (-3.19,3.75) |
| -3.64 (-5.75,-1.52) | -1.69 (-2.56,-0.81) | -1.54 (-2.96,-0.11) | -1.49 (-3.51,0.52) | -1.39 (-4.88,2.10) | -1.28 (-4.74,2.19) | -0.52 (-4.00,2.96) | -0.28 (-3.75,3.19) | CG |

League table--Disease Staging: NA

| GG | 0.60 (-6.12,7.33) | 1.74 (-3.59,7.07) | 2.16 (-3.18,7.49) | 2.35 (-4.35,9.05) | 2.86 (-3.84,9.56) | 3.33 (-3.37,10.03) | 3.71 (-1.07,8.50) |
| --- | --- | --- | --- | --- | --- | --- | --- |
| -0.60 (-7.33,6.12) | DS | 1.14 (-4.14,6.41) | 1.55 (-3.73,6.83) | 1.75 (-4.91,8.40) | 2.25 (-4.41,8.92) | 2.72 (-3.94,9.38) | 3.11 (-1.62,7.84) |
| -1.74 (-7.07,3.59) | -1.14 (-6.41,4.14) | HQ | 0.42 (-2.91,3.74) | 0.61 (-4.63,5.85) | 1.12 (-4.13,6.37) | 1.59 (-3.66,6.83) | 1.98 (-0.37,4.32) |
| -2.16 (-7.49,3.18) | -1.55 (-6.83,3.73) | -0.42 (-3.74,2.91) | YX | 0.19 (-5.05,5.44) | 0.70 (-4.55,5.95) | 1.17 (-4.08,6.42) | 1.56 (-0.79,3.91) |
| -2.35 (-9.05,4.35) | -1.75 (-8.40,4.91) | -0.61 (-5.85,4.63) | -0.19 (-5.44,5.05) | SK | 0.51 (-6.13,7.14) | 0.98 (-5.65,7.61) | 1.37 (-3.32,6.05) |
| -2.86 (-9.56,3.84) | -2.25 (-8.92,4.41) | -1.12 (-6.37,4.13) | -0.70 (-5.95,4.55) | -0.51 (-7.14,6.13) | DC | 0.47 (-6.17,7.10) | 0.86 (-3.84,5.55) |
| -3.33 (-10.03,3.37) | -2.72 (-9.38,3.94) | -1.59 (-6.83,3.66) | -1.17 (-6.42,4.08) | -0.98 (-7.61,5.65) | -0.47 (-7.10,6.17) | SX | 0.39 (-4.30,5.08) |
| -3.71 (-8.50,1.07) | -3.11 (-7.84,1.62) | -1.98 (-4.32,0.37) | -1.56 (-3.91,0.79) | -1.37 (-6.05,3.32) | -0.86 (-5.55,3.84) | -0.39 (-5.08,4.30) | CG |

Table A.37: Subgroup analysis: Scr

League table--Disease Staging: III

| GG | 0.20 (-1.59,2.00) | 0.32 (-1.15,1.79) | 0.37 (-0.98,1.72) | 0.51 (-0.59,1.60) | 0.47 (-1.30,2.24) | 0.67 (-0.81,2.15) | 0.79 (-0.44,2.03) | 0.93 (-0.41,2.28) | 1.05 (0.00,2.09) |
| --- | --- | --- | --- | --- | --- | --- | --- | --- | --- |
| -0.20 (-2.00,1.59) | DS | 0.12 (-1.67,1.91) | 0.17 (-1.53,1.86) | 0.30 (-1.19,1.80) | 0.27 (-1.78,2.31) | 0.46 (-1.34,2.26) | 0.59 (-1.01,2.19) | 0.73 (-0.95,2.42) | 0.84 (-0.62,2.30) |
| -0.32 (-1.79,1.15) | -0.12 (-1.91,1.67) | DC | 0.05 (-1.29,1.39) | 0.19 (-0.90,1.27) | 0.15 (-1.62,1.92) | 0.35 (-1.13,1.82) | 0.47 (-0.75,1.70) | 0.61 (-0.72,1.95) | 0.73 (-0.31,1.76) |
| -0.37 (-1.72,0.98) | -0.17 (-1.86,1.53) | -0.05 (-1.39,1.29) | ST | 0.14 (-0.78,1.05) | 0.10 (-1.57,1.77) | 0.30 (-1.06,1.65) | 0.42 (-0.66,1.50) | 0.56 (-0.64,1.77) | 0.68 (-0.18,1.53) |
| -0.51 (-1.60,0.59) | -0.30 (-1.80,1.19) | -0.19 (-1.27,0.90) | -0.14 (-1.05,0.78) | HQ | -0.04 (-1.51,1.43) | 0.16 (-0.94,1.26) | 0.29 (-0.45,1.02) | 0.43 (-0.48,1.33) | 0.54 (0.22,0.87) |
| -0.47 (-2.24,1.30) | -0.27 (-2.31,1.78) | -0.15 (-1.92,1.62) | -0.10 (-1.77,1.57) | 0.04 (-1.43,1.51) | KD | 0.20 (-1.58,1.97) | 0.32 (-1.26,1.90) | 0.46 (-1.20,2.13) | 0.58 (-0.86,2.01) |
| -0.67 (-2.15,0.81) | -0.46 (-2.26,1.34) | -0.35 (-1.82,1.13) | -0.30 (-1.65,1.06) | -0.16 (-1.26,0.94) | -0.20 (-1.97,1.58) | SK | 0.13 (-1.11,1.37) | 0.27 (-1.08,1.62) | 0.38 (-0.67,1.43) |
| -0.79 (-2.03,0.44) | -0.59 (-2.19,1.01) | -0.47 (-1.70,0.75) | -0.42 (-1.50,0.66) | -0.29 (-1.02,0.45) | -0.32 (-1.90,1.26) | -0.13 (-1.37,1.11) | YX | 0.14 (-0.93,1.21) | 0.26 (-0.40,0.91) |
| -0.93 (-2.28,0.41) | -0.73 (-2.42,0.95) | -0.61 (-1.95,0.72) | -0.56 (-1.77,0.64) | -0.43 (-1.33,0.48) | -0.46 (-2.13,1.20) | -0.27 (-1.62,1.08) | -0.14 (-1.21,0.93) | DH | 0.11 (-0.73,0.96) |
| -1.05 (-2.09,-0.00) | -0.84 (-2.30,0.62) | -0.73 (-1.76,0.31) | -0.68 (-1.53,0.18) | -0.54 (-0.87,-0.22) | -0.58 (-2.01,0.86) | -0.38 (-1.43,0.67) | -0.26 (-0.91,0.40) | -0.11 (-0.96,0.73) | CG |

League table--Disease Staging: NA

| DS | 1.98 (-0.81,4.77) | 1.86 (-1.52,5.25) | 2.67 (0.06,5.29) | 2.73 (-0.65,6.10) | 3.12 (0.34,5.90) | 3.23 (0.52,5.93) | 3.63 (1.06,6.20) | 3.99 (0.56,7.41) | 3.86 (1.44,6.29) |
| --- | --- | --- | --- | --- | --- | --- | --- | --- | --- |
| -1.98 (-4.77,0.81) | SK | -0.11 (-2.85,2.62) | 0.70 (-0.99,2.38) | 0.75 (-1.97,3.47) | 1.14 (-0.80,3.08) | 1.25 (-0.58,3.07) | 1.65 (0.03,3.27) | 2.01 (-0.77,4.79) | 1.89 (0.50,3.27) |
| -1.86 (-5.25,1.52) | 0.11 (-2.62,2.85) | DC | 0.81 (-1.74,3.36) | 0.86 (-2.46,4.19) | 1.25 (-1.47,3.98) | 1.36 (-1.28,4.01) | 1.76 (-0.74,4.27) | 2.12 (-1.25,5.50) | 2.00 (-0.36,4.36) |
| -2.67 (-5.29,-0.06) | -0.70 (-2.38,0.99) | -0.81 (-3.36,1.74) | HQ | 0.05 (-2.48,2.59) | 0.45 (-1.23,2.12) | 0.55 (-0.98,2.09) | 0.96 (-0.33,2.24) | 1.31 (-1.29,3.91) | 1.19 (0.22,2.16) |
| -2.73 (-6.10,0.65) | -0.75 (-3.47,1.97) | -0.86 (-4.19,2.46) | -0.05 (-2.59,2.48) | KD | 0.39 (-2.32,3.10) | 0.50 (-2.13,3.13) | 0.90 (-1.59,3.39) | 1.26 (-2.10,4.62) | 1.14 (-1.20,3.48) |
| -3.12 (-5.90,-0.34) | -1.14 (-3.08,0.80) | -1.25 (-3.98,1.47) | -0.45 (-2.12,1.23) | -0.39 (-3.10,2.32) | DH | 0.11 (-1.70,1.92) | 0.51 (-1.10,2.12) | 0.87 (-1.91,3.64) | 0.75 (-0.62,2.11) |
| -3.23 (-5.93,-0.52) | -1.25 (-3.07,0.58) | -1.36 (-4.01,1.28) | -0.55 (-2.09,0.98) | -0.50 (-3.13,2.13) | -0.11 (-1.92,1.70) | SX | 0.40 (-1.06,1.86) | 0.76 (-1.93,3.45) | 0.64 (-0.55,1.83) |
| -3.63 (-6.20,-1.06) | -1.65 (-3.27,-0.03) | -1.76 (-4.27,0.74) | -0.96 (-2.24,0.33) | -0.90 (-3.39,1.59) | -0.51 (-2.12,1.10) | -0.40 (-1.86,1.06) | YX | 0.36 (-2.20,2.91) | 0.24 (-0.61,1.08) |
| -3.99 (-7.41,-0.56) | -2.01 (-4.79,0.77) | -2.12 (-5.50,1.25) | -1.31 (-3.91,1.29) | -1.26 (-4.62,2.10) | -0.87 (-3.64,1.91) | -0.76 (-3.45,1.93) | -0.36 (-2.91,2.20) | GG | -0.12 (-2.53,2.29) |
| -3.86 (-6.29,-1.44) | -1.89 (-3.27,-0.50) | -2.00 (-4.36,0.36) | -1.19 (-2.16,-0.22) | -1.14 (-3.48,1.20) | -0.75 (-2.11,0.62) | -0.64 (-1.83,0.55) | -0.24 (-1.08,0.61) | 0.12 (-2.29,2.53) | CG |

Table A.38: Subgroup analysis: SUCRA values

|  | CG | HQ | YX | SX | DC | DS | DH | SK | KD | ST | GG |
| --- | --- | --- | --- | --- | --- | --- | --- | --- | --- | --- | --- |
| Disease Staging: III | | | | | | | | | | | |
| UAER | 17.2 | 60.7 | 56.8 | — | 36.5 | — | 54.5 | 51.5 | 49.7 | **92.6** | 30.5 |
| Scr | 15.0 | 55.3 | 35.2 | — | 63.4 | 65.1 | 27.7 | 43.8 | 54.0 | 61.6 | **78.8** |
| Disease Staging: NA | | | | | | | | | | | |
| UAER | 19.9 | 58.6 | 51.5 | 33.7 | 40.8 | 71.2 | — | 46.4 | — | — | **77.8** |
| Scr | 14.9 | 58.5 | 25.5 | 39.8 | 72.0 | **96.1** | 43.1 | 74.5 | 53.5 | — | 21.9 |

Appendix Table A.39-A.40: Sensitivity analysis

Table A.39: Sensitivity analysis: League table

| ST | 0.55 (-3.70,4.80) | 1.07 (-3.14,5.28) | 1.43 (-1.09,3.95) | 1.61 (-2.60,5.82) | 1.73 (-1.65,5.10) | 1.85 (-0.48,4.19) | 2.29 (-1.06,5.64) | 3.14 (-1.06,7.35) | 3.28 (-0.93,7.48) | 3.66 (1.49,5.84) |
| --- | --- | --- | --- | --- | --- | --- | --- | --- | --- | --- |
| -0.55 (-4.80,3.70) | DS | 0.52 (-4.62,5.65) | 0.88 (-2.99,4.75) | 1.06 (-4.07,6.19) | 1.18 (-3.30,5.65) | 1.30 (-2.45,5.05) | 1.73 (-2.72,6.19) | 2.59 (-2.53,7.72) | 2.72 (-2.40,7.85) | 3.11 (-0.54,6.76) |
| -1.07 (-5.28,3.14) | -0.52 (-5.65,4.62) | DH | 0.36 (-3.47,4.19) | 0.54 (-4.56,5.64) | 0.66 (-3.78,5.10) | 0.79 (-2.92,4.49) | 1.22 (-3.20,5.63) | 2.08 (-3.02,7.17) | 2.21 (-2.89,7.30) | 2.59 (-1.01,6.20) |
| -1.43 (-3.95,1.09) | -0.88 (-4.75,2.99) | -0.36 (-4.19,3.47) | YX | 0.18 (-3.65,4.00) | 0.30 (-2.59,3.18) | 0.42 (-1.12,1.97) | 0.86 (-2.00,3.71) | 1.71 (-2.11,5.54) | 1.85 (-1.98,5.67) | 2.23 (0.95,3.52) |
| -1.61 (-5.82,2.60) | -1.06 (-6.19,4.07) | -0.54 (-5.64,4.56) | -0.18 (-4.00,3.65) | KD | 0.12 (-4.32,4.55) | 0.25 (-3.46,3.95) | 0.68 (-3.74,5.09) | 1.54 (-3.56,6.63) | 1.67 (-3.43,6.76) | 2.06 (-1.55,5.66) |
| -1.73 (-5.10,1.65) | -1.18 (-5.65,3.30) | -0.66 (-5.10,3.78) | -0.30 (-3.18,2.59) | -0.12 (-4.55,4.32) | GG | 0.13 (-2.60,2.85) | 0.56 (-3.07,4.19) | 1.42 (-3.01,5.85) | 1.55 (-2.88,5.98) | 1.94 (-0.65,4.52) |
| -1.85 (-4.19,0.48) | -1.30 (-5.05,2.45) | -0.79 (-4.49,2.92) | -0.42 (-1.97,1.12) | -0.25 (-3.95,3.46) | -0.13 (-2.85,2.60) | HQ | 0.43 (-2.26,3.12) | 1.29 (-2.41,4.99) | 1.42 (-2.28,5.12) | 1.81 (0.95,2.66) |
| -2.29 (-5.64,1.06) | -1.73 (-6.19,2.72) | -1.22 (-5.63,3.20) | -0.86 (-3.71,2.00) | -0.68 (-5.09,3.74) | -0.56 (-4.19,3.07) | -0.43 (-3.12,2.26) | SK | 0.86 (-3.55,5.27) | 0.99 (-3.42,5.40) | 1.38 (-1.17,3.93) |
| -3.14 (-7.35,1.06) | -2.59 (-7.72,2.53) | -2.08 (-7.17,3.02) | -1.71 (-5.54,2.11) | -1.54 (-6.63,3.56) | -1.42 (-5.85,3.01) | -1.29 (-4.99,2.41) | -0.86 (-5.27,3.55) | DC | 0.13 (-4.96,5.22) | 0.52 (-3.08,4.12) |
| -3.28 (-7.48,0.93) | -2.72 (-7.85,2.40) | -2.21 (-7.30,2.89) | -1.85 (-5.67,1.98) | -1.67 (-6.76,3.43) | -1.55 (-5.98,2.88) | -1.42 (-5.12,2.28) | -0.99 (-5.40,3.42) | -0.13 (-5.22,4.96) | SX | 0.39 (-3.21,3.99) |
| -3.66 (-5.84,-1.49) | -3.11 (-6.76,0.54) | -2.59 (-6.20,1.01) | -2.23 (-3.52,-0.95) | -2.06 (-5.66,1.55) | -1.94 (-4.52,0.65) | -1.81 (-2.66,-0.95) | -1.38 (-3.93,1.17) | -0.52 (-4.12,3.08) | -0.39 (-3.99,3.21) | CG |

Table A.40: Sensitivity analysis: SUCRA values

|  | CG | HQ | YX | SX | DC | DS | DH | SK | KD | ST | GG |
| --- | --- | --- | --- | --- | --- | --- | --- | --- | --- | --- | --- |
| UAER | 12.8 | 50.4 | 60.4 | 26.9 | 28.8 | 72.0 | 64.2 | 42.3 | 54.1 | 85.4 | 52.6 |
| Scr | 9.9 | 54.6 | 36.9 | 42.4 | 47.1 | 81.0 | 38.8 | 89.4 | 51.6 | 44.5 | 53.8 |

Appendix Fig A.1: INPLASY Protocol


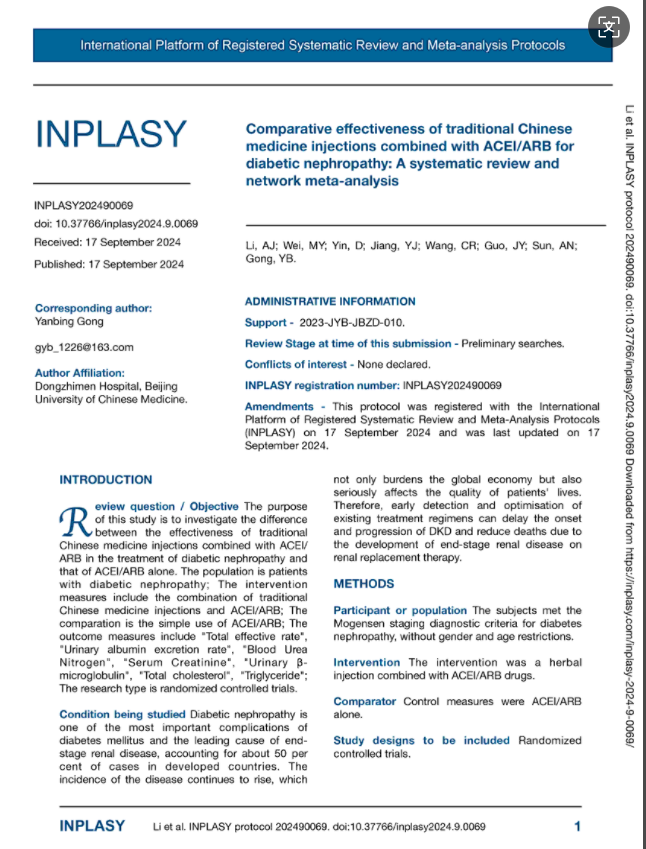


Appendix Fig A.2: Funnel plots


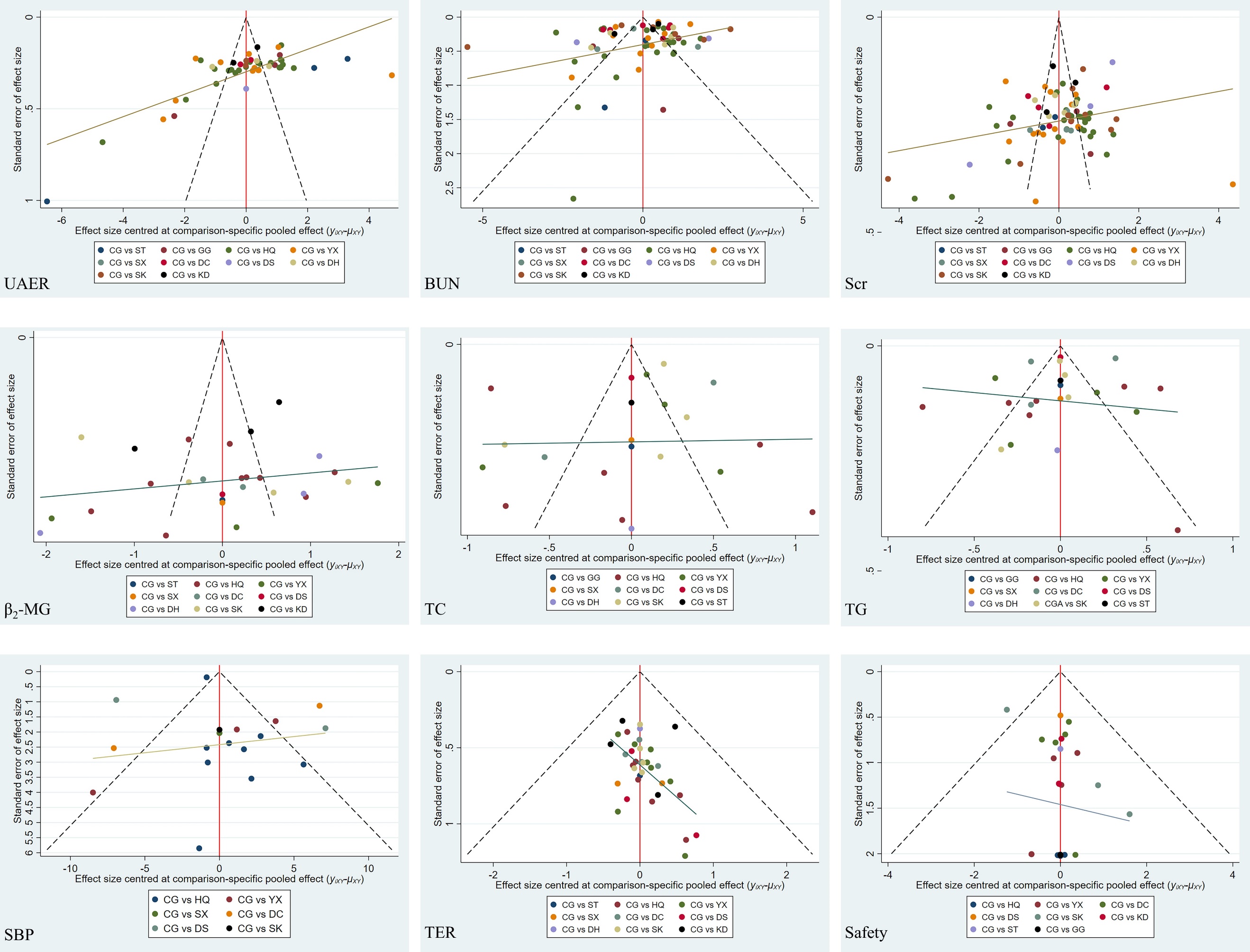


CG, control group. TER, total effective rate; UAER, urinary albumin excretion rate; BUN, blood urea nitrogen; Scr, serum creatinine; β2-MG, β2-microglobulin; TC, total cholesterol; TG, triglyceride; SBP, systolic blood pressure.
